# Synthesis of Vicinal *anti*-Amino Alcohols from *N*-*tert*-Butanesulfinyl Aldimines and Cyclopropanols

**DOI:** 10.1021/acs.joc.4c00198

**Published:** 2024-04-13

**Authors:** Sandra Hernández-Ibáñez, Juan F. Ortuño, Ana Sirvent, Carmen Nájera, José Miguel Sansano, Miguel Yus, Francisco Foubelo

**Affiliations:** † Departamento de Química Orgánica, Facultad de Ciencias, Universidad de Alicante, Apdo. 99, 03080 Alicante, Spain; ‡ Instituto de Síntesis Orgánica (ISO), Universidad de Alicante, Apdo. 99, 03080 Alicante, Spain; § Centro de Innovación en Química Avanzada (ORFEO−CINQA), Universidad de Alicante, Apdo. 99, 03080 Alicante, Spain

## Abstract

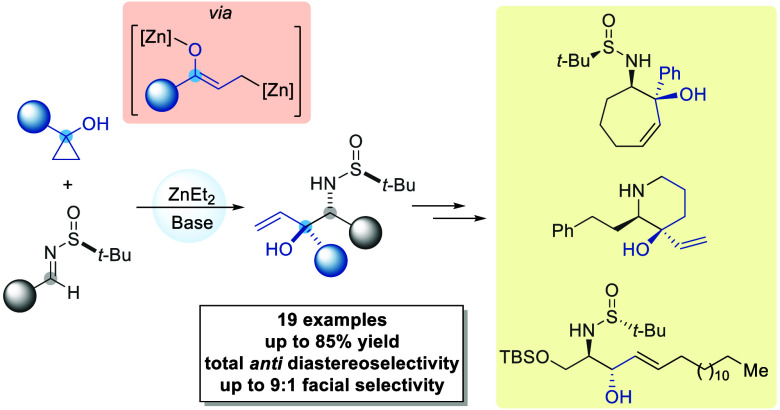

The stereoselective synthesis of vicinal amino alcohols derivatives from 1-substituted cyclopropanols and chiral *N*-*tert*-butanesulfinyl imines is described. Cyclopropanols are easily prepared from carboxylic esters upon reaction with ethylmagnesium bromide in the presence of titanium tetraisopropoxide and undergo carbon–carbon bond cleavage by means of diethylzinc to produce, upon base deprotonation, enolized zinc homoenolates, which react with chiral sulfinyl imines in a highly regio- and stereoselective manner.

## 1 Introduction

**Scheme 1:**
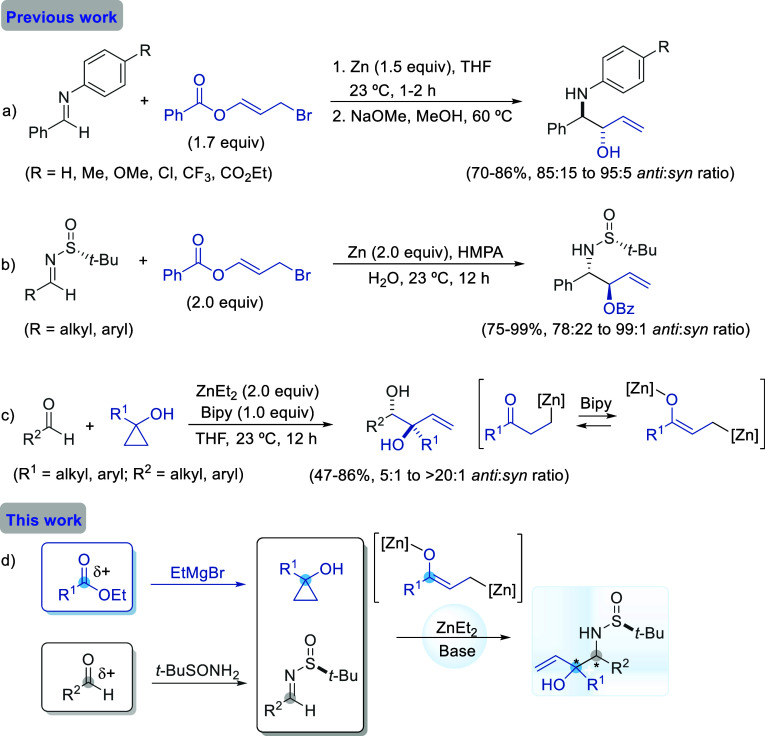
Hydroxyallylation of Imines and Carbonyl Compounds

The allylation of imines is a matter of significant synthetic interest. When allylation is executed in a stereoselective fashion, it provides access to enantioenriched homoallyl amines, which serve as invaluable building blocks.^1^ These compounds frequently feature as intermediates in numerous synthetic methodologies. The catalytic enantioselective allylation has been accomplished by employing substoichiometric quantities of chiral Lewis acids and/or bases.^2^ However, stereoselective allylations are more commonly conducted using stoichiometric amounts of chiral reagents, particularly when operating on a larger scale. Stereoselectivity in these processes can be attributed to either the chiral imine (substrate diastereocontrol) or chiral allylating reagents (reagent diastereocontrol).^3^ Diastereoselective allylations of imines necessitate consideration of two critical factors: the face selectivity, influenced by the chiral substrate or reagent, and regioselectivity, which comes into play when substituted allylic reagents are involved. In the latter case, the formation of a contiguous stereocenter with a relative *anti*- or *syn*-configuration, facilitated through an ordered acyclic or cyclic transition state, is also feasible, with the metal playing a pivotal role in the governing transition state. Hydroxyallylation of imines holds special significance as it leads to the formation of vicinal allylic amino alcohols. This chemical motif is highly versatile and intriguing, garnering significant attention in the realms of natural product chemistry and drug discovery. Its unique structural features bestow compounds bearing this motif with a wide array of biological activities, rendering them promising candidates for pharmaceutical and medicinal applications. The most straightforward method for achieving hydroxy allylation of imines involves the use of hydroxy allyl organometallic compounds.^4^ Among these, 3-acyloxyallyl bromides have proven to be easily manageable and highly efficient precursors of these oxidofunctionalized allyl organometallic compounds.^5^ In this context, Norrby and Madsen established a protocol for the synthesis of vicinal amino alcohols. This method utilized a Barbier-type reaction between an imine and 3-benzoyloxyallyl bromide in the presence of zinc metal. The resulting addition products underwent debenzylation to yield amino alcohols in good yields, with diastereomeric ratios favoring the *anti*-isomer at greater than 85:15 (Scheme 1a).^6^ Similarly, Xu and Lin successfully developed a diastereoselective α-hydroxyallylation approach for the asymmetric synthesis of various β-amino-α-vinyl alcohols. They achieved this by employing highly diastereoselective Zn-promoted benzoyloxyallylation of chiral *N-tert*-butanesulfinyl imines with 3-bromopropenyl benzoate at room temperature, resulting in a wide range of vinylic amino alcohol derivatives in excellent yields. The diastereomeric ratios reached up to 99:1 in favor of the *anti*-isomers (Scheme 1b).^7^ This methodology was applied in a key step of the synthesis of the marine alkaloid ecteinascidin 743, a compound known for its potent cytostatic properties and antitumor activity. Ecteinascidin 743 is currently utilized in the treatment of soft-tissue sarcoma and ovarian cancer.^8^

Concurrently, cyclopropanols have gained significant attention in organic synthesis as precursors of three-carbon building blocks.^9^ These easily accessible compounds^10^ contain strained three-membered rings that readily undergo carbon–carbon bond cleavage to release energy. Depending on the conditions used to promote ring opening, intermediates such as organometallic homoenolates, β-keto radicals, and *O-*protonated ketones are formed. Homoenolates are of particular interest due to the presence of two closely located carbon atoms with nucleophilic (carbon–metal bond) and electrophilic (carbonyl group) character. Transition metal derivatives of homoenolates have been employed in cross-coupling reactions.^11^ On the other hand, the direct diastereoselective synthesis of *anti*-1,2-diols, with oxygen atoms bonded to secondary and allylic tertiary carbon atoms, as reported by Sekiguchi and Yoshikai, is of significant importance. The zinc homoenolate, formed upon the opening of the cyclopropanol, is in equilibrium under relatively strong basic conditions with an enolized homoenolate. The enolized homoenolate acts as an oxyallyl nucleophile, reacting with the aldehyde to serve as an oxygen-substituted allylating reagent. The resulting vicinal diols exhibit high diastereoselectivity, favoring the *anti*-isomers (Scheme 1c).^12^ Importantly, enolized homoenolates can also function as enolates, depending on the reaction conditions and the electrophilic partner.^13^

Given our research group’s expertise in the diastereoselective allylation of *N-tert*-butanesulfinyl imines,^14^ and considering the bibliographic antecedents previously commented, we deemed it worthwhile to investigate the allylation of these chiral imines using zinc enolized homoenolates formed by the cleavage of 1-substituted cyclopropanols. We aimed to determine the influence of the *tert*-butanesulfinyl group on the stereoselectivity of the process. Starting cyclopropanols can be synthesized from carboxylic esters and ethylmagnesium bromide using the Kulinkovich protocol.^15^ Since sulfinyl imines are typically prepared from a carbonyl compound and *tert*-butanesulfinamide, the expected outcome is the formation of vicinal amino alcohol derivatives resulting from the coupling of two carbon atoms, ostensibly with the same polarity if considering their precursors. This transformation can be viewed as an umpolung reaction with respect to the enolized homoenolate (Scheme 1d).

## Results and Discussion

The study of the allylation of *N-tert*-butanesulfinyl imines began with the optimization of the reaction conditions. For this purpose, we selected the (*R*s)-*tert*-butanesulfinamide **1a** derived from 3-phenylpropanal and 1-phenylcyclopropanol (**2b**) as the model substrates. Initially, we tested the conditions described by Sekiguchi and Yoshikai for selectively obtaining *anti*-1,2-diols (Scheme 1c).^12^ The reaction was conducted with 2 equiv of Et_2_Zn and 1 equiv of bipyridine as a base in THF at 23 °C for 1 h. Unfortunately, the reaction did not proceed under these conditions, and our analysis using ^1^H NMR indicated the presence of only the starting imine **1a** (Table 1, entry 1). Conversely, we repeated the same conditions but raised the temperature to 60 °C. Fortunately, we obtained allylation products **3ab** and **4ab** in a 4:1 ratio, with complete consumption of the starting imine **1a** (Table 1, entry 2). Similar results were obtained when Et_3_N was used as the base (Table 1, entry 3). However, when the reaction was carried out at 40 °C, we did not obtain the desired reaction products **3ab** and **4ab**. Instead, we observed the starting imine **1a** and what appeared to be decomposition products of the starting materials (Table 1, entry 4). We noted that, apparently, the autocondensation product of imine **1a** was the sole reaction product when EtONa in EtOH was used as the base (Table 1, entry 5). Complete decomposition of the starting materials occurred when DBU and Cs_2_CO_3_ were used as bases (Table 1, entries 6 and 7). When the reaction was conducted using pyridine or DIPEA as bases, we obtained the allylation products **3ab** and **4ab** with excellent conversions but poorer diastereoselectivity (Table 1, entries 8 and 9). Subsequently, we performed the reaction similarly to entry 3 but with the addition of 3 equiv of CuCN·2LiCl. Surprisingly, under these conditions, we obtained a diastereomeric mixture of **3ab** and **4ab**, with a reversed diastereoselectivity, where diastereoisomer **4ab** became the major component in an almost 1:3 ratio (Table 1, entry 10). Lastly, we conducted the reaction under the same conditions as in entry 10 but in the absence of a base. Unfortunately, we observed only decomposition products (Table 1, entry 11).

**Table 1:**
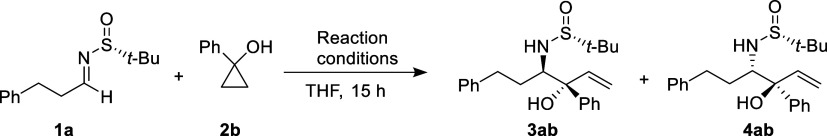
Optimization of the Reaction Conditions^a^

Entry	Reaction conditions	**1a**/**3ab**/**4ab** ratio^b^
1	Bpy (1 equiv), Et_2_Zn (2 equiv), 23 °C	100/0/0
2	Bpy (1 equiv), Et_2_Zn (2 equiv), 60 °C	0/82/18
3	Et_3_N (1 equiv), Et_2_Zn (2 equiv), 60 °C	0/83/17
4	Et_3_N (1 equiv), Et_2_Zn (2 equiv), 40 °C	100/0/0^c^
5	EtONa (2M, EtOH, 1 equiv), Et_2_Zn (2 equiv), 60 °C	--d
6	DBU (1 equiv), Et_2_Zn (2 equiv), 60 °C	--c
7	Cs_2_CO_3_ (1 equiv), Et_2_Zn (2 equiv), 60 °C	--c
8	Pyridine (1 equiv), Et_2_Zn (2 equiv), 60 °C	0/74/26
9	DIPEA (1 equiv), Et_2_Zn (2 equiv), 60 °C	0/60/40
10	Et_3_N (1 equiv), CuCN·2LiCl (0.5M, 3 equiv), Et_2_Zn (2 equiv), 60 °C	0/26/74
11	CuCN·2LiCl (0.5M, 3 equiv), Et_2_Zn (2 equiv), 60 °C	--c

a Reactions were carried out with 0.2 mmol of **1a** and **2b**.

b Ratio determined by analysis of the ^1^H NMR spectrum of the crude reaction mixture.

c Total decomposition of the starting material **1a** took place, and the expected products **3/4** were not observed.

d Autocondensation product of imine **1a** seems to be the reaction product.

**Scheme 2:**
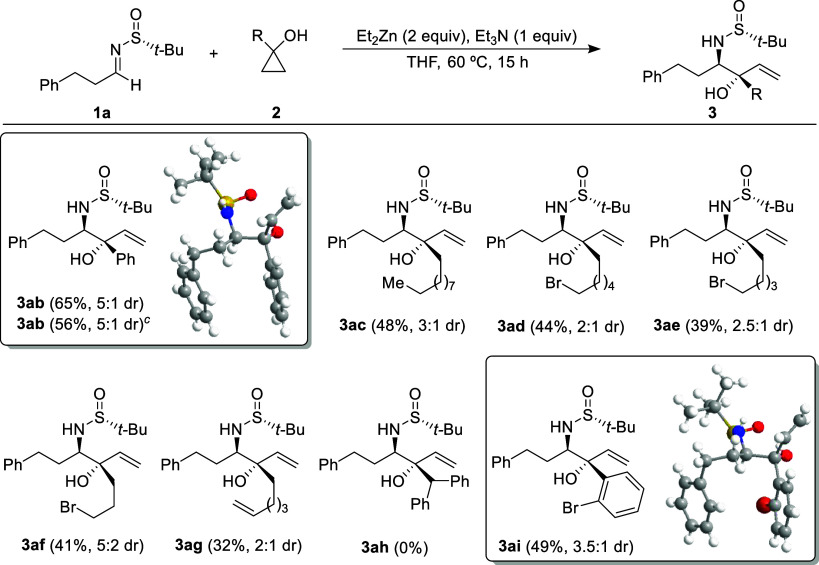
Hydroxyallylation of Imine **1a** with Different 1-Substituted Cyclopropanols **2**^,^^,^ Reactions were carried out with 0.3 mmol of **1a** and **2**. Ratio determined by analysis of the ^1^H NMR spectrum of the crude reaction mixture. Reaction was carried out with 1.0 mmol of **1a** and **2**.

With the optimized conditions in hand (Table 1, entry 3), in order to obtain diastereoisomer **3** as the major reaction product, we initially explored the reaction of various cyclopropanols **2** with sulfinyl imine **1a** (Scheme 2). Alkyl and aryl cyclopropanol derivatives **2** participated in the hydroxyallylation with imine **1a**, yielding the corresponding products **3ab**–**ai** in moderately isolated yields. The scope of the reaction was explored at a 0.3 mmol scale, with the exception of 1-phenylcyclopropanol (**2b**), for which the reaction was also conducted on a 1.0 mmol-scale. This resulted in the production of the amino alcohol derivative **3ab** with an 56% isolated yield, a little bit lower to that achieved at the 0.3 mmol scale. In Scheme 2, diastereomeric ratios of diastereoisomers **3** (always the major isomer) and **4** are provided in parentheses. Unfortunately, the reactions with 1-benzhydrylcyclopropan-1-ol (**2h**) did not yield the expected 1,2-aminoalcohol **3ah**. Instead, only decomposition products were observed. Regarding the configuration of compounds **3**, it was determined through crystal X-ray analysis (see the Supporting Information) of solid compounds **3ab** and **3ai**.^16^ The configurations of the remaining compounds **3** were assigned by analogy, assuming that they all were formed through the same stereochemical pathway. The allylation occurred via nucleophilic attack on the *Si* face of imines with *R*_S_ configuration, resulting in vicinal amino alcohols with a relative *anti*-configuration.

**Scheme 3:**
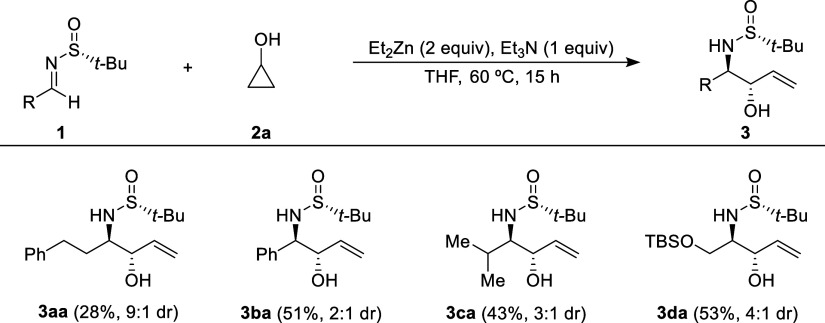
Hydroxyallylation of Imines **1** with Cyclopropanol **2a**^,^ Reactions were carried out with 0.3 mmol of **1** and **2a**. Ratio determined by analysis of the ^1^H NMR spectrum of the crude reaction mixture.

It is worth noting that hydroxyallylations can also be carried out under the same reaction conditions outlined in Scheme 2, using cyclopropanol (**2a**) as the hydroxyallylation agent. Interestingly, in the work of Sekiguchi and Yoshikai, all the examples presented involve substituted cyclopropanols.^12^ The reaction products obtained in these cases are secondary allylic alcohols with a sulfonamide group in the neighboring position. The isolated yields, indicated in parentheses in Scheme 3 for the major reaction product **3**, were moderate, as were the diastereomeric ratios in the case of imines derived from benzaldehyde (**1b**), isobutyraldehyde (**1c**), and *O*-TBS-protected hydroxyacetaldehyde (**1d**). In contrast, better diastereoselectivity, albeit with lower yield, was observed in the case of the imine derived from 3-phenylpropanal (**1a**) (Scheme 3).

**Scheme 4:**
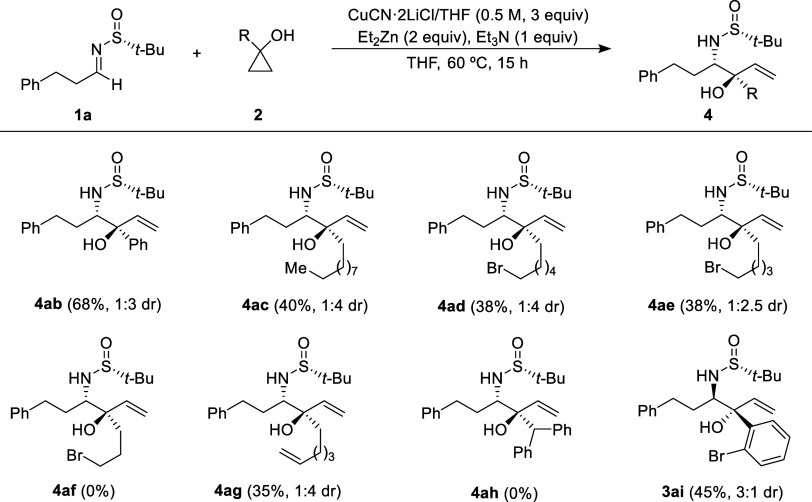
Hydroxyallylation of Imine **1a** with Different 1-Substituted Cyclopropanols 2 in the Presence of CuCN·2LiCl^,^ Reactions were carried out with 0.3 mmol of **1a** and **2**. Ratio determined by analysis of the ^1^H NMR spectrum of the crude reaction mixture.

We proceeded to investigate the reaction’s scope using the same cyclopropanols **2** and sulfinyl imine **1a**. However, we applied the reaction conditions outlined in entry 10 of Table 1, aiming to favor the formation of diastereoisomers **4** as the major reaction products. The only deviation from the conditions previously employed in Scheme 2 was the addition of 3 equiv of CuCN·2LiCl (0.5 M in THF) (Scheme 4). Consequently, we consistently obtained the corresponding products **4ab**–**ai** in moderately isolated yields as the primary components of the reaction products, with aminoalcohol derivatives **3** appearing as minor isomers (diastereomeric ratios are indicated in parentheses). Moreover, as observed with the prior conditions, the reaction failed to occur with 1-benzhydrylcyclopropan-1-ol (**2h**). Additionally, in the case of cyclopropanol **2f** (3-bromopropyl derivative), the reaction did not yield the expected product **4af**. An unexpected outcome arose in the hydroxyallylation involving 1-(2-bromophenyl)cyclopropanol (**2i**), as the predominant reaction product was the *anti*-isomer **3ai**, the same one produced when working without copper cyanide. The configuration of compounds **4** was established after a simple sulfur atom epimerization of the sulfinyl unit in compound **3ae** (vide infra). In this scenario, the nucleophilic attack of the allylic reagent occurred preferentially on the *Re* face of imines with *R*_S_ configuration, resulting in 1,2-aminoalcohol derivatives **4** with relative *anti*-configurations. The formation of *syn*-isomers resulting from an attack on the Si face of the imine could be an alternative possibility.

**Scheme 5:**
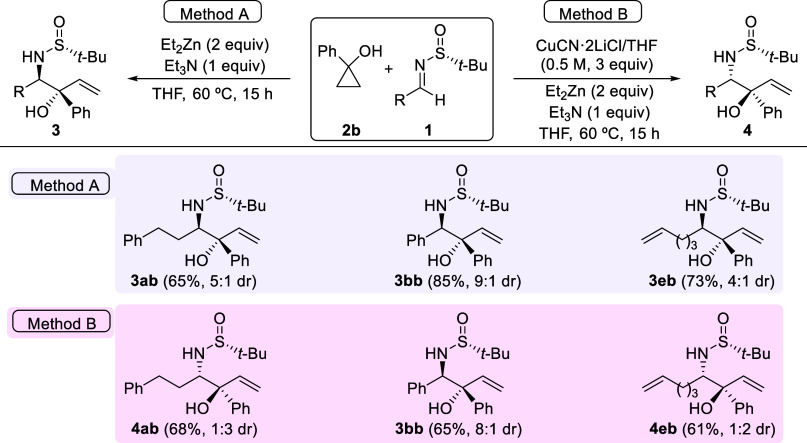
Hydroxyallylation of Imines **1a**, **1b**, and **1e** with Cyclopropanol **2b** in the Presence and Absence of CuCN·2LiCl^,^ Reactions were carried out with 0.3 mmol of **1** and **2b**. Ratio determined by analysis of the ^1^H NMR spectrum of the crude reaction mixture.

**Figure 1:**
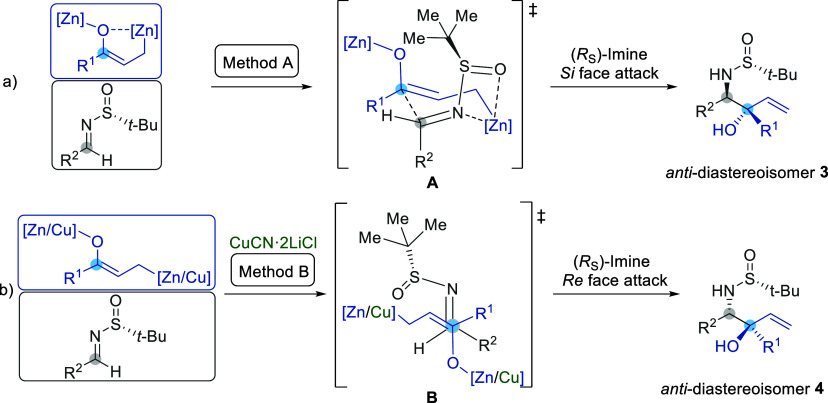
Speculative working models for explaining the stereochemical outcomes of the hydroxyallylations.

To expand the range of the reaction, we also investigated the reaction of 1-phenylcyclopropanol (**2b**) with various sulfinyl imines **1** under the reaction conditions outlined in Schemes 2 (Method A) and 4 (Method B). We observed slightly higher diastereoselectivities and yields when employing the reaction conditions of Method A. This consistently led to the formation of the *anti*-diastereoisomer **3** as the major component of the reaction mixture, resulting from the nucleophilic attack on the *Si* face of imines with *R*_S_ configuration (Scheme 5). In contrast, *anti*-diastereoisomers resulting from the nucleophilic attack on the *Re* face of imines with *R*_S_ configuration predominated when using the reaction conditions of Method B, producing compounds **4**. Surprisingly, there was an exception to this general rule. When 1-phenylcyclopropanol (**2b**) reacted with the imine derived from benzaldehyde **1b** under the conditions of Method B, it yielded the *anti*-isomer **3bb** in fairly good yield with an 8:1 diastereomeric ratio. This anomalous result may be elucidated by considering steric or π–π stacking interactions between the two adjacent phenyl groups, potentially directing the process predominantly through the primary operating mechanism under the reaction conditions of Method A (see Figure 1a).

**Scheme 6:**
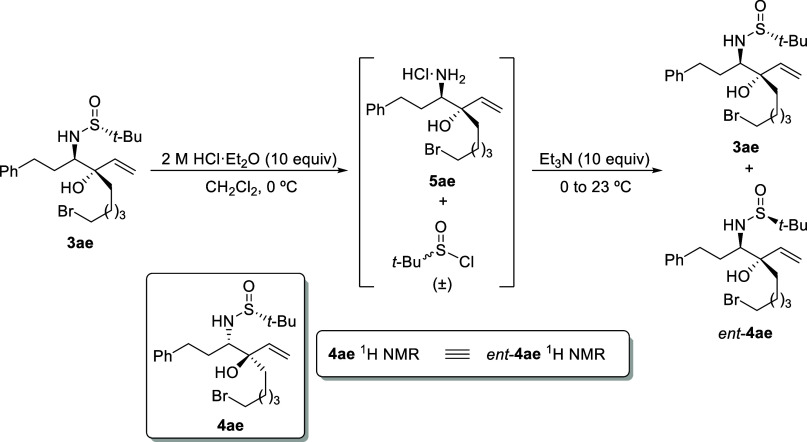
Epimerization of the Sulfur Atom of Amino Alcohol Derivative **3ae**

We determined the configuration of vicinal amino alcohol derivatives **4** by conducting a straightforward epimerization of the sulfur atom within the sulfinyl group under acidic conditions, employing a nonprotic solvent such as dichloromethane.^17^ We selected the *anti*-isomer **3ae** as our model substrate. Under these conditions, the sulfinyl group was removed from the sulfinamide, resulting in the formation of the hydrochloride derivative **5ae** and racemic *tert*-butanesulfinyl chloride. Subsequent addition of triethylamine led to the generation of sulfonamide derivatives as a mixture of diastereoisomers, **3ae** and *ent*-**4ae** (Scheme 6). We analyzed the ^1^H NMR spectrum of the crude reaction mixture and identified two distinct sets of signals: one corresponding to the starting *anti*-isomer **3ae** and another set perfectly matching the signals of compound **4ae**. This unequivocally confirmed the *anti*-relative configuration of amino alcohol derivatives **4**, as the ^1^H NMR spectra of *ent*-**4ae** and **4ae** were entirely identical (see Supporting Information). Furthermore, a simple TLC experiment revealed identical *R*_f_ values for **4ae** and the epimerized product of **3ae** (*ent*-**4ae**).

**Scheme 7:**
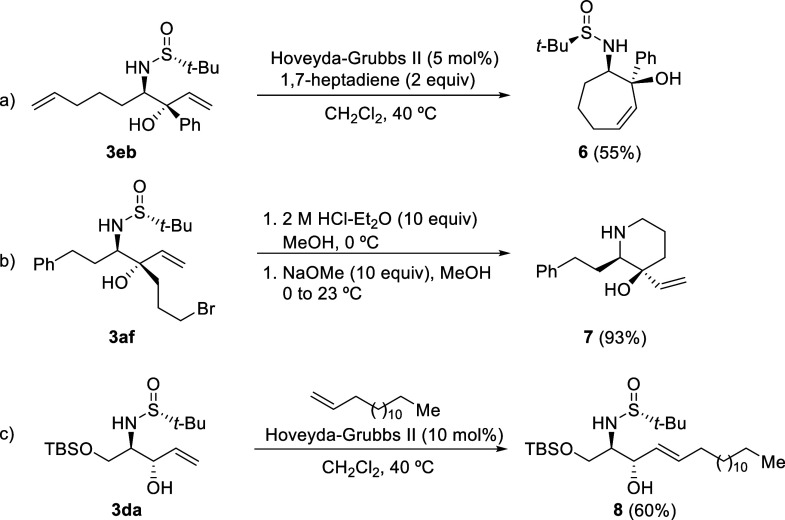
Synthetic Transformations of Amino Alcohol Derivatives **3**

It is important to emphasize that amino alcohol derivatives **3** and **4**, featuring various functionalities within their structures, hold significant potential for applications in synthesis as precursors to both carbo- and heterocyclic compounds, as well as others with potential biological activity. In this context, we present three examples of direct transformations of these amino alcohols in Scheme 7. To illustrate, the ring-closing metathesis of diene amino alcohol **3eb** yielded the aminocycloheptenol derivative **6** in 55% yield (Scheme 7a). Conversely, the bromo-substituted compound **3af** was converted into hydroxy vinyl piperidine **7**, nearly quantitatively, through the removal of the sulfinyl group under acidic conditions, followed by a basic workup (Scheme 7b). Lastly, cross-metathesis involving the selectively protected amino diol derivative **3da** and pentadec-1-ene resulted in *N-tert*-butanesulfinyl 1-*O*-TBS protected l-sphingosine **8** in 60% yield (Scheme 7c).

The stereochemical outcomes of these reactions were elucidated by considering the formation of an enolized zinc homoenolate with a *Z* configuration, featuring a stabilizing interaction between the oxygen of the enolate and the zinc-bound homoenolate, which interacts with the chiral sulfinyl imine **1**. The formation of the enolized zinc homoenolate is supported by DFT calculations.^12^ These calculations, conducted for the reaction of this organometallic intermediate with aldehydes, anticipate that the allylation proceeds through a chairlike Zimmerman–Traxler transition state. In this transition state, the larger alkyl or aryl group of the aldehyde occupies an axial position, while the R^1^ group of the enolate is positioned equatorially. In the case of *N-tert*-butanesulfinyl imines **1**, under the reaction conditions of Method A, we propose a working model **A** in which the zinc homoenolate coordinates with both the nitrogen of the imine and the oxygen of the sulfinyl group, forming a bicyclic environment composed of a 4-membered ring (N–S–O–Zn), and a chairlike 6-membered ring. In this configuration, the R^1^ group of the enolate assumes a pseudoequatorial position, while the R^2^ and sulfinyl groups of the imine are diaxially disposed. In this scenario, hydroxyallylation occurs at the *Si* face of the imine with *R*_S_ configuration, yielding the *anti*-diastereoisomer **3**, consistent with experimental observations (Figure 1a). The formation of other *anti*-diastereoisomers **4** under the reaction conditions of Method B could be explained by considering an acyclic model. When hydroxyallylation is conducted in the presence of a large excess of copper cyanide, the formation of cyclic intermediates could be avoided due to the formation of zinc–copper couple intermediates with saturated coordination spheres, avoiding the formation of cyclic intermediates. Consequently, the addition to the imine may occur through an open transition state. The most stable configuration of the imine assumes an *s-cis* conformation, with the *Re* face of *N-tert*-butanesulfinyl imines **1** (with *R*_S_ configuration) being the less hindered face. In this manner, the configuration of the stereogenic center bonded to the nitrogen in the hydroxyallylated product is the opposite of that obtained when using Method A. Regarding the relative *anti*-configuration, it can be explained by considering an open transition state (Transition State B) that minimizes destabilizing dipole interactions with the nitrogen of the imine and the oxygen of the enolate in an antiperiplanar disposition, thereby accounting for the preferential formation of *anti*-diastereoisomer **4** (Figure 1b). As a result, both reaction pathways illustrated in Figure 1 could be operating to varying extents under the reaction conditions for Methods A and B, which explains why mixtures of diastereoisomers **3** and **4** were consistently obtained.

## Conclusions

In conclusion, our investigation into the hydroxyallylation of *N-tert*-butanesulfinyl imines with cyclopropanols has provided valuable insights into the diastereoselective formation of vicinal amino alcohols. Notably, our research not only fine-tuned the reaction conditions for this transformation but also showcased the method’s versatility across a wide range of substrates. Furthermore, by unraveling the stereochemical outcomes of these reactions, we gained significant understanding of the mechanistic intricacies governing the preferential formation of *anti*-diastereoisomers as the predominant reaction products. These densely functionalized amino alcohol derivatives hold promise for diverse synthetic applications, exemplified by their direct conversion into various valuable carbo- and heterocyclic compounds. This work offers new avenues for the efficient synthesis of complex molecules with potential biological activities. As such, it holds great potential in the realms of medicinal chemistry and natural product synthesis.

## Experimental Section

### General Remarks

Reagents and solvents were purchased from commercial suppliers and used as received. (*R*)-*tert*-Butanesulfinamide was a gift of Medalchemy (>99% ee by chiral HPLC on a Chiracel AS column, 90:10 *n*-hexane/*i*-PrOH, 1.2 mL/min, λ = 222 nm). Optical rotations were measured using a Jasco P-1030 polarimeter with a thermally jacketed 5 cm cell at approximately 23 °C, and concentrations (*c*) are given in g/100 mL. Low-resolution mass spectra (EI) were obtained with an Agilent GC/MS5973N spectrometer at 70 eV, and fragment ions in *m*/*z* with relative intensities (%) in parentheses. High-resolution mass spectra (HRMS) were also carried out in the electron impact mode (EI) at 70 eV and on a Finnigan MAT95S spectrometer equipped with a time-of-flight (TOF) analyzer and the samples were ionized by ESI techniques and introduced through an ultrahigh pressure liquid chromatography (UPLC) model. NMR spectra were recorded at 300 or 400 MHz for ^1^H NMR and at 75 or 100 MHz for ^13^C NMR with a Bruker AV300 Oxford or a Bruker AV400 spectrometers, respectively, using CDCl_3_ as solvent, and TMS as internal standard (0.00 ppm). The data are reported as s = singlet, d = doublet, t = triplet, q = quartet, m = multiplet or unresolved, br s = broad signal, coupling constant(s) in Hz, integration. ^13^C NMR spectra were recorded with ^1^H-decoupling at 100 MHz and referenced to CDCl_3_ at 77.16 ppm. DEPT-135 experiments were performed to assign CH, CH_2_, and CH_3_. TLCs were performed on prefabricated Merck aluminum plates with silica gel 60 coated with fluorescent indicator F_254_ and were visualized with phosphomolybdic acid (PMA) stain. The *R*_f_ values were calculated under these conditions. Flash chromatography was carried out on handpacked columns of silica gel 60 (230–400 mesh). Compounds **1a** [R = Ph(CH_2_)_2_],^18^**1b** (R = Ph),^19^**1c** (R = *i*-Pr),^19^**1d** (R = TBSOCH_2_),^20^ and **1e** [R = CH_2_=CH(CH_2_)_3_]^21^ were prepared from the corresponding aldehyde and (*R*)-*tert*-butanesulfinamide according to previously published procedures. Compound **2a** was commercially available. Compounds **2b** (R = Ph),^22^**2c** [R = CH_3_(CH_2_)_9_],^23^**2d** [R = Br(CH_2_)_6_], **2e** [R = Br(CH_2_)_5_],^24^**2f** [R = Br(CH_2_)_3_],^25^**2g** [R = CH_2_=CH(CH_2_)_3_], **2h** (R = Ph_2_CH), and **2i** (R = 2-BrC_6_H_4_)^26^ were prepared from the corresponding ethyl ester and ethylmagnesium bromide in the presence of titanium tetraisopropoxide.^15^

#### General Procedure for the Reaction of Sulfinyl Imines **1** and Cyclopropanols **2** and Synthesis of Compounds **3** (Method A)

To a solution of corresponding cyclopropanol **2** (0.3 mmol) in dry THF (1.8 mL) was sequentially added Et_3_N (42 μL, 0.3 mmol), a 1 M solution of Et_2_Zn in toluene (0.6 mL, 0.6 mmol), and the corresponding sulfinyl imine imine **1** (0.3 mmol). The reaction mixture was stirred at 60 °C (oil bath) for 15 h. Then, the reaction was cooled to room temperature and hydrolyzed with a saturated aqueous solution of NH_4_Cl (5.0 mL), extracted with AcOEt (3 × 10 mL), the combined organic phases dried over anhydrous MgSO_4_, and the solvents evaporated (15 Torr). The residue was purified by column chromatography (silica gel, hexane/EtOAc) to yield pure compounds **3**.

##### (*R*_S_,3*S*,4*R*)-4-Amino-*N*-(*tert*-butanesulfinyl)-6-phenylhex-1-en-3-ol (**3aa**)

Following the general procedure, compound **3aa** (24.8 mg, 0.085 mmol, 28%) was obtained as a yellow solid; mp 66–68 °C (hexane/CH_2_Cl_2_); [α]_D_^23^ = +33.3 (*c* = 0.99, CH_2_Cl_2_); *R*_f_ = 0.30 (hexane/EtOAc, 1:1); ^1^H NMR (300 MHz, CDCl_3_) δ 7.40–7.19 (m, 5H), 5.89 (ddt, *J* = 15.8, 10.6, 4.6 Hz, 1H), 5.43–5.36 (m, 1H), 5.29 (dt, *J* = 10.6, 1.6 Hz, 1H), 4.34–4.20 (m, 1H), 3.47–3.34 (m, 1H), 2.90 (dddt, *J* = 18.9, 14.1, 9.3, 4.4 Hz, 2H), 2.79–2.62 (m, 2H), 1.32 (s, 9H); ^13^C{^1^H} NMR (75 MHz, CDCl_3_) δ 141.4 (C), 136.5 (CH), 128.5 (CH), 128.3 (CH), 126.0 (CH), 117.0 (CH_2_), 75.0 (CH), 60.8 (CH), 56.3 (C), 32.1 (CH_2_), 31.2 (CH_2_), 22.9 (CH_3_); LRMS (EI) *m*/*z* 295 (M^+^, < 1%), 150 (14), 134 (20), 117 (19), 104 (24), 91 (86), 70 (19), 57 (37), 43 (100); HRMS (EI-TOF) Calcd for C_16_H_25_NO_2_S [M^+^] 295.1606, found 295.1613.

##### (*R*_S_,3*R*,4*R*)-4-Amino-*N*-(*tert*-butanesulfinyl)-3,6-diphenylhex-1-en-3-ol (**3ab**)

Following the general procedure, compound **3ab** (68.5 mg, 0.19 mmol, 65%) was obtained as a white solid. The reaction was also performed with 1.0 mmol of cyclopropanol **2b** (134.2 mg, 1.0 mmol), 1.0 mmol of sulfinyl imine **1a** (237.4 mg), 1.0 mmol of Et_3_N (101.2 mg, 139 μL), and 2.0 mmol of a 1 M solution of Et_2_Zn in toluene (2.0 mL), in 6.0 mL of dry THF. After stirring the reaction at 60 °C (oil bath) for 15 h, it was cooled to room temperature and hydrolyzed with a saturated aqueous solution of NH_4_Cl (15.0 mL), extracted with AcOEt (3 × 15 mL), the combined organic phases dried over anhydrous MgSO_4_, and the solvents evaporated (15 Torr). The residue was purified by column chromatography (silica gel, hexane/EtOAc, 4:1) to yield pure compound **3ab** (208.1 mg, 0.56 mmol, 56%) as a white solid; mp 130–133 °C (hexane/CH_2_Cl_2_); [α]_D_^23^ = −7.9 (*c* = 1.24, CH_2_Cl_2_); *R*_f_ = 0.25 (hexane/EtOAc, 3:1); ^1^H NMR (400 MHz, CDCl_3_) δ 7.43–7.09 (m, 8H), 7.03–6.79 (m, 2H), 6.45 (dd, *J* = 17.1, 10.7 Hz, 1H), 5.83–5.54 (m, 2H), 5.21 (s, 1H), 3.66 (d, *J* = 10.3 Hz, 1H), 3.44 (td, *J* = 10.7, 2.0 Hz, 1H), 2.73 (ddd, *J* = 13.5, 8.8, 4.4 Hz, 1H), 2.51–2.33 (m, 1H), 1.71–1.54 (m, 2H), 1.34 (s, 9H); ^13^C{^1^H} NMR (100 MHz, CDCl_3_) δ 143.3 (C), 140.9 (C), 137.3 (CH), 128.5 (CH), 128.5 (CH), 127.7 (CH), 126.9 (CH), 126.2 (CH), 119.8 (CH_2_), 78.8 (C), 67.4 (CH), 56.8 (C), 34.4 (CH_2_), 32.7 (CH_2_), 23.1 (CH_3_); LRMS (EI) *m*/*z* 297 (M^+^–C_4_H_9_O, 2%), 239 (12), 238 (78), 234 (34), 164 (52), 143 (55), 133 (53), 117 (100), 91 (98), 57 (40), 55 (36); HRMS (EI-TOF) Calcd for C_18_H_20_NO_2_S [M^+^–C_4_H_9_] 314.1215, found 314.1212.

##### (*R*_S_,3*R*,4*S*)-3-Amino-*N*-(*tert*-butanesulfinyl)-1-phenyl-4-vinyltetradecan-4-ol (**3ac**)

Following the general procedure, compound **3ac** (60.7 mg, 0.14 mmol, 48%) was obtained as a yellow wax; [α]_D_^23^ = −2.1 (*c* = 1.80, CH_2_Cl_2_); *R*_f_ = 0.54 (hexane/EtOAc, 3:1); ^1^H NMR (400 MHz, CDCl_3_) δ 7.39–7.04 (m, 5H), 5.77 (dd, *J* = 17.2, 10.6 Hz, 1H), 5.46–5.33 (m, 2H), 4.67 (s, 1H), 3.56 (d, *J* = 10.2 Hz, 1H), 3.22–3.04 (m, 1H), 2.85 (ddd, *J* = 13.7, 9.1, 4.6 Hz, 1H), 2.62–2.47 (m, 1H), 1.55–1.41 (m, 4H), 1.31 (s, 9H), 1.29–1.16 (m, 16H), 0.88 (t, *J* = 6.9 Hz, 3H); ^13^C{^1^H} NMR (100 MHz, CDCl_3_) δ 141.45 (C), 140.1 (CH), 128.7 (CH), 128.65 (CH), 126.3 (CH), 118.0 (CH_2_), 76.7 (C), 65.4 (CH), 56.7 (C), 38.3 (CH_2_), 34.8 (CH_2_), 33.0 (CH_2_), 32.1 (CH_2_), 30.2 (CH_2_), 29.8 (CH_2_), 29.7 (CH_2_), 29.7 (CH_2_), 29.5 (CH_2_), 23.1 (CH_3_), 22.8 (CH_2_), 14.3 (CH_3_); LRMS (EI) *m*/*z* 379 (M^+^–C_4_H_8_, 1%), 361 (3), 298 (15), 238 (70), 197 (27), 182 (15), 164 (31), 157 (11), 134 (44), 117 (79), 91 (100), 57 (77), 55 (27), 43 (27), 41 (25); HRMS (EI-TOF) Calcd for C_26_H_45_NO_2_S [M^+^] 435.3171, found 435.3168.

##### (*R*_S_,3*R*,4*S*)-3-Amino-10-bromo-*N*-(*tert*-butanesulfinyl)-1-phenyl-4-vinyldecan-4-ol (**3ad**)

Following the general procedure, compound **3ad** (60.5 mg, 0.13 mmol, 44%) was obtained as a yellow oil; [α]_D_^23^ = +9.5 (*c* = 0.80, CH_2_Cl_2_); *R*_f_ = 0.29 (hexane/EtOAc, 3:1); ^1^H NMR (400 MHz, CDCl_3_) δ 7.38–7.07 (m, 5H), 5.77 (dd, *J* = 17.2, 10.6 Hz, 1H), 5.49–5.24 (m, 2H), 4.68 (s, 1H), 3.56 (d, *J* = 10.2 Hz, 1H), 3.38 (t, *J* = 6.9 Hz, 2H), 3.20–3.05 (m, 1H), 2.93–2.72 (m, 1H), 2.63–2.45 (m, 1H), 1.81 (dt, *J* = 14.5, 6.9 Hz, 2H), 1.60 (s, 4H), 1.55–1.44 (m, 3H), 1.31 (s, 9H), 1.37–1.26 (m, 3H); ^13^C{^1^H} NMR (100 MHz, CDCl_3_) δ 141.4 (C), 139.9 (CH), 128.7 (CH), 128.7 (CH), 128.6 (CH), 126.35 (CH), 118.1 (CH_2_), 76.7 (C), 65.4 (CH), 56.8 (C), 38.2 (CH_2_), 34.8 (CH_2_), 34.1 (CH_2_), 32.9 (CH_2_), 32.8 (CH_2_), 29.3 (CH_2_), 28.2 (CH_2_), 23.15 (CH_3_), 22.7 (CH_2_); LRMS (EI) *m*/*z* 322 [M^+^(^81^Br)–C_4_H_10_NO_2_S, 12%], 320 [M^+^(^79^Br)–C_4_H_10_NO_2_S, 12%], 239 (10), 238 (63), 221 (14), 219 (14), 182 (15), 164 (33), 157 (16), 134 (33), 117 (75), 91 (100), 67 (13), 57 (60), 55 (29), 41 (24); HRMS (EI-TOF) Calcd for C_18_H_27_NO_2_S [M^+^–C_4_H_9_Br] 321.1762, found 321.176.

##### (*R*_S_,3*R*,4*S*)-3-Amino-9-bromo-*N*-(*tert*-butanesulfinyl)-1-phenyl-4-vinylnonan-4-ol (**3ae**)

Following the general procedure, compound **3ae** (52.0 mg, 0.12 mmol, 39%) was obtained as a yellow oil; [α]_D_^23^ = +6.7 (*c* = 2.30, CH_2_Cl_2_); *R*_f_ = 0.35 (hexane/EtOAc, 3:1); ^1^H NMR (400 MHz, CDCl_3_) δ 7.39–7.08 (m, 5H), 5.78 (dd, *J* = 17.2, 10.6 Hz, 1H), 5.50–5.30 (m, 2H), 4.73 (s, 1H), 3.58 (d, *J* = 10.2 Hz, 1H), 3.38 (t, *J* = 6.8 Hz, 2H), 3.20–3.07 (m, 1H), 2.93–2.80 (m, 1H), 2.63–2.48 (m, 1H), 2.02–1.93 (m, 2H), 1.87–1.73 (m, 2H), 1.63–1.45 (m, 4H), 1.40–1.33 (m, 2H), 1.33 (s, 9H); ^13^C{^1^H} NMR (100 MHz, CDCl_3_) δ 141.3 (C), 139.8 (CH), 128.7 (CH), 128.7 (CH), 126.3 (CH), 118.2 (CH_2_), 76.6 (C), 65.3 (CH), 56.8 (C), 38.05 (CH_2_), 34.8 (CH_2_), 34.1 (CH_2_), 32.8 (CH_2_), 32.8 (CH_2_), 28.6 (CH_2_), 23.1 (CH_3_), 22.0 (CH_2_); LRMS (EI) *m*/*z* 308 [M^+^(^81^Br)–C_4_H_10_NO_2_S, 13%], 306 [M^+^(^79^Br)–C_4_H_10_NO_2_S, 13%], 238 (61), 207 (14), 205 (14), 182 (14), 164 (34), 157 (19), 134 (29), 117 (74), 91 (100), 67 (12), 57 (53), 55 (35), 41 (19); HRMS (EI-TOF): Calcd for C_17_H_26_NO_2_S [M^+^–C_4_H_8_Br] 308.1684, found 308.1695.

##### (*R*_S_,3*R*,4*S*)-3-Amino-7-bromo-*N*-(*tert*-butanesulfinyl)-1-phenyl-4-vinylheptan-4-ol (**3af**)

Following the general procedure, compound **3af** (51.2 mg, 0.12 mmol, 41%) was obtained as a yellow oil; [α]_D_^23^ = +6.1 (*c* = 1.20, CH_2_Cl_2_); *R*_f_ = 0.20 (hexane/EtOAc, 3:1); ^1^H NMR (400 MHz, CDCl_3_) δ 7.34–7.08 (m, 6H), 5.71 (dd, *J* = 17.1, 10.6 Hz, 1H), 5.35–5.00 (m, 2H), 3.99–3.76 (m, 3H), 3.28 (ddd, *J* = 9.6, 7.0, 2.7 Hz, 1H), 2.97–2.81 (m, 1H), 2.55 (ddd, *J* = 13.7, 10.2, 6.7 Hz, 1H), 2.30–2.13 (m, 1H), 1.96–1.79 (m, 3H), 1.79–1.61 (m, 2H), 1.29 (s, 9H); ^13^C{^1^H} NMR (100 MHz, CDCl_3_) δ 142.2 (C), 140.0 (CH), 128.6 (CH), 128.5 (CH), 126.0 (CH), 114.35 (CH_2_), 87.8 (C), 68.9 (CH), 61.1 (CH_2_), 56.5 (C) 35.45 (CH_2_), 33.9 (CH_2_), 32.95 (CH_2_), 25.2 (CH_2_), 23.2 (CH_3_); LRMS (EI) *m*/*z* 279 (M^+^–C_4_H_9_Br, 24%), 239 (12), 238 (73), 216 (12), 187 (19), 182 (26), 164 (67), 134 (46), 118 (11), 117 (100), 104 (17), 99 (18), 97 (92), 91 (92), 79 (13), 57 (46), 55 (65), 41 (21); HRMS (EI-TOF): Calcd for C_15_H_21_NOS [M^+^–C_4_H_9_BrO] 263.1344, found 263.1337.

##### (*R*_S_,3*R*,4*S*)-3-Amino-*N*-(*tert*-butanesulfinyl)-1-phenyl-4-vinyldec-9-en-4-ol (**3ag**)

Following the general procedure, compound **3ag** (36.2 mg, 0.09 mmol, 32%) was obtained as a yellow oil; [α]_D_^23^ = −4.6 (*c* = 1.13, CH_2_Cl_2_); *R*_f_ = 0.41 (hexane/EtOAc, 3:1); ^1^H NMR (400 MHz, CDCl_3_) δ 7.43–6.97 (m, 5H), 5.89–5.62 (m, 2H), 5.51–5.22 (m, 2H), 5.05–4.85 (m, 2H), 4.67 (s, 1H), 3.56 (d, *J* = 10.2 Hz, 1H), 3.13 (m, 1H), 2.85 (m, 1H), 2.55 (m, 1H), 1.98 (m, 4H), 1.57–1.39 (m, 4H), 1.31 (s, 9H), 1.33–1.25 (m, 2H); ^13^C{^1^H} NMR (100 MHz, CDCl_3_) δ 141.4 (C), 140.0 (CH), 139.15 (CH), 128.7 (CH), 128.65 (CH), 128.7 (CH), 128.6 (CH), 128.5 (CH), 128.4 (CH), 126.3 (CH), 118.1 (CH_2_), 114.4 (CH_2_), 76.7 (C), 65.4 (CH), 56.8 (C), 38.1 (CH_2_), 34.8 (CH_2_), 33.8 (CH_2_), 33.0 (CH_2_), 29.5 (CH_2_), 23.1 (CH_3_), 22.4 (CH_2_); LRMS (EI) *m*/*z* 321 (M^+^+1–C_4_H_8_, 1%), 320 (2), 238 (54), 182 (13), 164 (32), 139 (17), 134 (31), 117 (75), 91 (100), 83 (14), 57 (53), 55 (41), 41 (28); HRMS (EI-TOF) Calcd for C_18_H_27_NO_2_S [M^+^–C_4_H_8_] 321.1762, found 321.1759.

##### (*R*_S_,3*R*,4*R*)-4-Amino-3-(2-bromophenyl)-*N*-(*tert*-butanesulfinyl)-6-phenylhex-1-en-3-ol (**3ai**)

Following the general procedure, compound **3ai** (66.2 mg, 0.15 mmol, 49%) was obtained as a white solid; mp 38–40 °C (hexane/CH_2_Cl_2_); [α]_D_^23^ = −5.0 (*c* = 1.10, CH_2_Cl_2_); *R*_f_ = 0.19 (hexane/EtOAc, 3:1); ^1^H NMR (400 MHz, CDCl_3_) δ 7.58 (dd, *J* = 7.9, 1.4 Hz, 1H), 7.38–7.04 (m, 7H), 6.96 (dd, *J* = 7.9, 1.6 Hz, 1H), 6.52 (dd, *J* = 16.9, 10.6 Hz, 1H), 5.90–5.54 (m, 2H), 5.18 (s, 1H), 4.60 (td, *J* = 9.9, 3.2 Hz, 1H), 3.75 (d, *J* = 9.9 Hz, 1H), 2.83–2.69 (m, 1H), 2.54–2.40 (m, 1H), 1.63–1.45 (m, 2H), 1.32 (s, 9H); ^13^C{^1^H} NMR (100 MHz, CDCl_3_) δ 141.1 (C), 137.65 (CH), 136.25 (CH), 130.8 (CH), 129.2 (CH), 128.6 (CH), 128.5 (CH), 127.0 (CH), 126.2 (CH), 121.9 (C), 120.1 (CH_2_), 79.7 (C), 62.3 (CH), 56.6 (C), 35.1 (CH_2_), 32.9 (CH_2_), 23.1 (CH_3_); LRMS (EI) *m*/*z* 314 [M^+^(^81^Br)–C_4_H_9_NO_2_S, 16%], 312 [M^+^(^79^Br)–C_4_H_9_NO_2_S, 16%], 239 (12), 238 (74), 182 (22), 164 (55), 134 (18), 132 (49), 117 (100), 91 (99), 77 (13), 57 (40), 55 (13), 41 (11); HRMS (EI-TOF) Calcd for C_18_H_20_NO_2_S [M^+^–C_4_H_8_Br] 314.1215, found 314.1212.

##### (*R*_S_,1*R*,2*S*)-1-Amino-*N*-(*tert*-butanesulfinyl)-1-phenylbut-3-en-2-ol (**3ba**)

Following the general procedure, compound **3ba** (40.8 mg, 0.153 mmol, 51%) was obtained as a yellow solid; mp 52–54 °C (hexane/CH_2_Cl_2_); [α]_D_^23^ = +27.8 (*c* = 0.98, CH_2_Cl_2_); *R*_f_ = 0.31 (hexane/EtOAc, 1:1); ^1^H NMR (300 MHz, CDCl_3_) δ 7.34–7.18 (m, 6H), 5.55 (ddd, *J* = 17.2, 10.5, 4.8 Hz, 1H), 5.32 (dq, *J* = 17.3, 1.6 Hz, 1H), 5.23–5.14 (m, 1H), 4.57 (dd, *J* = 7.4, 4.2 Hz, 1H), 4.50–4.41 (m, 1H), 4.11 (d, *J* = 7.4 Hz, 1H), 1.12 (s, 9H); ^13^C{^1^H} NMR (75 MHz, CDCl_3_) δ 138.3 (C), 135.6 (CH), 128.5 (CH), 127.8 (CH) 127.1 (CH), 117.8 (CH_2_), 74.7 (CH), 60.7 (CH), 56.9 (C), 22.9 (CH_3_); LRMS (EI) *m*/*z* 267 (M^+^, < 1%), 210 (12), 154 (37), 130 (25), 104 (15), 77 (11), 57 (32), 43 (100); HRMS (EI-TOF) Calcd for C_14_H_21_NO_2_S (M^+^) 267.1293; found 267.1279.

##### (*R*_S_,1*R*,2*R*)-1-Amino-*N*-(*tert*-butanesulfinyl)-1,2-diphenylbut-3-en-2-ol (**3bb**)

Following the general procedure, compound **3bb** (87.6 mg, 0.26 mmol, 85%) was obtained as a white solid; mp 192–195 °C (hexane/CH_2_Cl_2_); [α]_D_^23^ = +25.9 (*c* = 2.44, CH_2_Cl_2_); *R*_f_ = 0.18 (hexane/EtOAc, 3:1); ^1^H NMR (400 MHz, CDCl_3_) δ 7.44–7.10 (m, 8H), 6.95–6.84 (m, 2H), 6.35 (dd, *J* = 17.1, 10.7 Hz, 1H), 5.55–5.33 (m, 2H), 4.66 (d, *J* = 5.6 Hz, 1H), 4.11 (d, *J* = 5.3 Hz, 1H), 3.87 (s, 1H), 1.20 (s, 9H); ^13^C{^1^H} NMR (100 MHz, CDCl_3_) δ 142.5 (C), 138.4 (CH), 137.5 (C), 129.0 (CH), 128.3 (CH), 128.0 (CH), 127.9 (CH), 127.8 (CH), 127.7 (CH), 126.7 (CH), 117.4 (CH_2_), 79.0 (C), 68.5 (CH), 22.8 (CH_3_); LRMS (EI) *m*/*z* 269 (M^+^–C_4_H_9_OH, 1%), 210 (31), 206 (40), 154 (100), 136 (30), 133 (47), 106 (41), 105 (27), 77 (23), 57 (29), 55 (30); HRMS (EI-TOF) Calcd for C_16_H_16_NO_2_S [M^+^–C_4_H_9_] 286.0902, found 286.0914.

##### (*R*_S_,3*S*,4*R*)-4-Amino-*N*-(*tert*-butanesulfinyl)-5-methylhex-1-en-3-ol (**3ca**)

Following the general procedure, compound **3ca** (30.0 mg, 0.13 mmol, 43%) was obtained as a yellow solid; mp 35–37 °C (hexane/CH_2_Cl_2_); [α]_D_^23^ = +31.6 (*c* = 1.05, CH_2_Cl_2_); *R*_f_ = 0.35 (hexane/EtOAc, 1:1); ^1^H NMR (300 MHz, CDCl_3_) δ 5.93 (ddd, *J* = 18.1, 10.3, 3.9 Hz, 1H), 5.49 (dt, *J* = 17.3, 1.9 Hz, 1H), 5.41 (dd, *J* = 10.9, 4.3 Hz, 1H), 4.70 (dt, *J* = 15.2, 7.1 Hz, 1H), 4.52 (br s, 1H), 1.28 (s, 9H), 1.05 (d, *J* = 6.7 Hz, 3H), 1.01 (d, *J* = 6.7 Hz, 3H); ^13^C{^1^H} NMR (75 MHz, CDCl_3_) δ 133.4 (CH), 119.8 (CH_2_), 71.10 (CH) 61.9 (CH), 29.7 (C), 27.6 (CH), 22.4 (CH_3_), 19.7 (CH_3_), 19.5 (CH_3_); LRMS (EI) *m*/*z* 233 (M^+^, < 1%), 207 (20), 183 (27), 152 (11), 108 (24), 77 (11), 57 (3), 43 (100); HRMS (EI-TOF) Calcd for C_7_H_13_NOS [M^+^–C_4_H_10_O] 159.0718, found 159.0713.

##### (*R*_S_,3*S*,4*R*)-4-Amino-*N*-(*tert*-butanesulfinyl)-5-[(*tert*-butyldimethylsilyl)oxy]pent-1-en-3-ol (**3da**)

Following the general procedure, compound **3da** (53.30 mg, 0.159 mmol, 53%) was obtained as a yellow oil; [α]_D_^23^ = +31.6 (*c* = 1.05, CH_2_Cl_2_); *R*_f_ = 0.21 (hexane/EtOAc, 2:1); ^1^H NMR (300 MHz, CDCl_3_) δ 5.91 (ddd, *J* = 17.2, 10.6, 5.0 Hz, 1H), 5.37 (dt, *J* = 17.2, 1.7 Hz, 1H), 5.24 (dt, *J* = 10.6, 1.6 Hz, 1H), 4.28 (br s, 1H), 3.99 (dd, *J* = 10.3, 3.5 Hz, 2H), 3.83 (dd, *J* = 10.2, 4.5 Hz, 1H), 3.33 (dt, *J* = 4.8, 3.9 Hz, 1H), 1.23 (s, 10H), 0.89 (s, 9H), 0.09 (s, 6H), 0.08 (s, 6H); ^13^C{^1^H} NMR (75 MHz, CDCl_3_) δ 137.5 (CH), 116.3 (CH_2_), 73.9 (CH), 63.8 (CH_2_), 59.9 (CH), 56.2 (C), 25.8 (CH_3_), 22.7 (CH_3_), 18.1 (C), −5.5 (CH_3_), −5.6 (CH_3_); LRMS (EI) *m*/*z* 335 (M^+^, < 1%), 279 (32), 261 (7), 204 (21), 173 (28), 156 (13), 141 (44), 116 (64), 100 (18), 83 (64), 73 (99), 57 (100), 41 (34); HRMS (EI-TOF) Calcd for C_7_H_13_NOS [M^+^–C_3_H_5_O] 278.1581, found 278.1576.

##### (*R*_S_,3*R*,4*R*)-4-Amino-*N*-(*tert*-butanesulfinyl)-3-phenylnona-1,8-dien-3-ol (**3eb**)

Following the general procedure, compound **3eb** (73.5 mg, 0.22 mmol, 73%) was obtained as a white solid; mp 106–109 °C (hexane/CH_2_Cl_2_); [α]_D_^23^ = −62.4 (*c* = 0.86, CH_2_Cl_2_); *R*_f_ = 0.29 (hexane/EtOAc, 3:1); ^1^H NMR (400 MHz, CDCl_3_) δ 7.54–7.18 (m, 5H), 6.48 (dd, *J* = 17.0, 10.7 Hz, 1H), 5.76–5.54 (m, 3H), 5.22 (s, 1H), 4.90–4.79 (m, 2H), 3.56 (d, *J* = 10.3 Hz, 1H), 3.48–3.37 (m, 1H), 1.99–1.84 (m, 1H), 1.85–1.71 (m, 1H), 1.54–1.35 (m, 1H), 1.28 (s, 9H), 1.25–1.05 (m, 3H); ^13^C{^1^H} NMR (100 MHz, CDCl_3_) δ 143.6 (C), 138.2 (CH), 137.3 (CH), 128.5 (CH), 127.65 (CH), 126.9 (CH_2_), 119.6 (CH), 114.85 (CH_2_), 78.8 (C), 68.5 (CH), 56.7 (C), 32.9 (CH_2_), 32.0 (CH_2_), 25.9 (CH_2_), 23.0 (CH_3_); LRMS (EI) *m*/*z* 261 (M^+^–C_4_H_9_OH, 2%), 203 (12), 202 (96), 198 (32), 169 (10), 156 (26), 146 (79), 133 (100), 130 (20), 128 (29), 105 (35), 98 (21), 94 (16), 81 (55), 77 (30), 57 (85), 55 (83), 41 (34); HRMS (EI-TOF) Calcd for C_15_H_21_NO_2_S [M^+^–C_4_H_8_] 279.1293, found 279.1295.

#### General Procedure for the Reaction of Sulfinyl Imines **1** and Cyclopropanols **2** in the Presence of CuCN·LiCl and Synthesis of Compounds **4** (Method B)

To a solution of corresponding cyclopropanol **2** (0.3 mmol) in dry THF (1.8 mL) was sequentially added Et_3_N (42 μL, 0.3 mmol), a 1 M solution of Et_2_Zn in toluene (0.6 mL, 0.6 mmol), and a 0.5 M solution of CuCN·LiCl in THF (1.8 mL, 0.9 mmol). The reaction mixture was stirred at 23 °C for 15 min. After that, the corresponding sulfinyl imine **1** (0.3 mmol) was added to the reaction mixture and continued stirring at 60 °C (oil bath) for 15 h. Then, the reaction was cooled to room temperature and hydrolyzed with a saturated aqueous solution of NH_4_Cl (5.0 mL), extracted with AcOEt (3 × 10 mL), the combined organic phases dried over anhydrous MgSO_4_, and the solvents evaporated (15 Torr). The residue was purified by column chromatography (silica gel, hexane/EtOAc) to yield pure compounds **4**.

##### (*R*_S_,3*S*,4*S*)-4-Amino-*N*-(*tert*-butanesulfinyl)-3,6-diphenylhex-1-en-3-ol (**4ab**)

Following the general procedure, compound **4ab** (72.1 mg, 0.20 mmol, 68%) was obtained as a yellow oil; [α]_D_^23^ = −24.2 (*c* = 1.70, CH_2_Cl_2_); *R*_f_ = 0.28 (hexane/EtOAc, 3:1); ^1^H NMR (400 MHz, CDCl_3_) δ 7.43–7.14 (m, 10H), 6.04 (dd, *J* = 17.0, 10.7 Hz, 1H), 5.43 (dd, *J* = 17.0, 1.6 Hz, 1H), 5.17 (dd, *J* = 10.7, 1.6 Hz, 1H), 4.82 (s, 1H), 3.69–3.61 (m, 1H), 3.48 (d, *J* = 3.2 Hz, 1H), 2.97–2.83 (m, 1H), 2.66–2.55 (m, 1H), 2.28–2.16 (m, 2H), 1.06 (s, 9H); ^13^C{^1^H} NMR (100 MHz, CDCl_3_) δ 144.5 (C), 141.5 (C), 140.3 (C), 137.3 (CH), 128.9 (CH), 128.7 (CH), 128.6 (CH), 127.2 (CH), 126.9 (CH), 126.15 (CH), 125.7 (CH), 114.8 (CH_2_), 79.1 (C), 64.2 (CH), 55.8 (C), 32.45 (CH_2_), 29.8 (CH_2_), 28.3 (CH_2_), 22.7 (CH_3_); LRMS (EI) *m*/*z* 297 (M^+^–C_4_H_9_O, 1%), 239 (11), 238 (69), 234 (33), 182 (16), 164 (56), 143 (49), 134 (34), 133 (63), 117 (97), 105 (22), 91 (100), 57 (44), 55 (39); HRMS (EI-TOF) Calcd for C_18_H_20_NO_2_S [M^+^–C_4_H_9_] 314.1215, found 314.1203.

##### (*R*_S_,3*S*,4*R*)-3-Amino-*N*-(*tert*-butanesulfinyl)-1-phenyl-4-vinyltetradecan-4-ol (**4ac**)

Following the general procedure, compound **4ac** (52.28 mg, 0.12 mmol, 40%) was obtained as a yellow wax; [α]_D_^23^ = −48.3 (*c* = 0.38, CH_2_Cl_2_); *R*_f_ = 0.29 (hexane/EtOAc, 3:1); ^1^H NMR (400 MHz, CDCl_3_) δ 7.40–7.03 (m, 5H), 5.71 (dd, *J* = 17.3, 10.8 Hz, 1H), 5.36–5.12 (m, 2H), 3.37 (d, *J* = 7.2 Hz, 1H), 3.11 (ddd, *J* = 10.4, 7.2, 2.2 Hz, 1H), 2.97 (ddd, *J* = 13.9, 9.1, 4.6 Hz, 1H), 2.88 (s, 1H), 2.70 (dt, *J* = 13.9, 8.4 Hz, 1H), 1.58–1.41 (m, 2H), 1.30 (s, 9H), 1.28–1.14 (m, 12H), 0.92–0.84 (m, 5H); ^13^C{^1^H} NMR (100 MHz, CDCl_3_) δ 141.75 (C), 140.1 (CH), 128.8 (CH), 128.6 (CH), 128.5 (CH), 126.8 (CH), 115.3 (CH_2_), 77.5 (C), 64.2 (CH), 56.7 (C), 38.3 (CH_2_), 32.5 (CH_2_), 32.4 (CH_2_), 32.05 (CH_2_), 30.2 (CH_2_), 29.7 (CH_2_), 29.7 (CH_2_), 29.45 (CH_2_), 23.2 (CH_2_), 23.2 (CH_3_), 22.8 (CH_2_), 14.3 (CH_3_); LRMS (EI) *m*/*z* 379 (M^+^–C_4_H_8_, 2%), 298 (13), 238 (57), 197 (18), 182 (16), 181 (22), 164 (34), 134 (38), 117 (100), 91 (99), 57 (75), 55 (28), 43 (33), 41 (27); HRMS (EI-TOF) Calcd for C_26_H_45_NO_2_S [M^+^] 435.3171, found 435.3181.

##### (*R*_S_,3*S*,4*R*)-3-Amino-10-bromo-*N*-(*tert*-butanesulfinyl)-1-phenyl-4-vinyldecan-4-ol (**4ad**)

Following the general procedure, compound **4ad** (52.3 mg, 0.11 mmol, 38%) was obtained as a yellow wax; [α]_D_^23^ = −29.5 (*c* = 0.90, CH_2_Cl_2_); *R*_f_ = 0.14 (hexane/EtOAc, 3:1); ^1^H NMR (400 MHz, CDCl_3_) δ 7.46–7.09 (m, 5H), 5.72 (dd, *J* = 17.2, 10.8 Hz, 1H), 5.39–5.14 (m, 2H), 3.47 (d, *J* = 7.1 Hz, 1H), 3.40 (t, *J* = 6.6 Hz, 2H), 3.17–3.06 (m, 1H), 3.06–2.87 (m, 3H), 2.82–2.59 (m, 3H), 1.90–1.72 (m, 5H), 1.32 (s, 9H), 1.44–1.01 (m, 4H); ^13^C{^1^H} NMR (100 MHz, CDCl_3_) δ 141.7 (C), 139.9 (CH), 128.8 (CH), 128.7 (CH), 128.6 (CH), 128.6 (CH), 128.4 (CH), 126.1 (CH), 115.5 (CH_2_), 77.4 (C), 64.4 (CH), 56.8 (C), 38.0 (CH_2_), 34.1 (CH_2_), 32.8 (CH_2_), 32.5 (CH_2_), 29.85 (CH_2_), 29.2 (CH_2_), 28.2 (CH_2_), 23.2 (CH_3_), 23.0 (CH_2_); LRMS (EI) *m*/*z* 322 [M^+^(^81^Br)–C_4_H_10_NO_2_S, 10%], 320 [M^+^(^79^Br)–C_4_H_10_NO_2_S, 10%], 238 (53), 221 (11), 219 (10), 182 (14), 164 (37), 157 (17), 134 (26), 117 (77), 91 (100), 67 (13), 57 (58), 55 (28), 41 (25); HRMS (EI-TOF) Calcd for C_18_H_28_NO_2_S [M^+^–C_4_H_8_Br] 322.1841, found 322.1838.

##### (*R*_S_,3*S*,4*R*)-3-Amino-9-bromo-*N*-(*tert*-butanesulfinyl)-1-phenyl-4-vinylnonan-4-ol (**4ae**)

Following the general procedure, compound **4ae** (50.7 mg, 0.11 mmol, 38%) was obtained as a colorless wax; [α]_D_^23^ = −40.3 (*c* = 2.10, CH_2_Cl_2_); *R*_f_ = 0.18 (hexane/EtOAc, 3:1); ^1^H NMR (400 MHz, CDCl_3_) δ 7.40–7.16 (m, 5H), 5.72 (dd, *J* = 17.3, 10.8 Hz, 1H), 5.38–5.15 (m, 2H), 3.47 (d, *J* = 7.1 Hz, 1H), 3.39 (t, *J* = 6.8 Hz, 2H), 3.11 (ddd, *J* = 10.5, 7.2, 2.1 Hz, 1H), 3.05–2.90 (m, 2H), 2.77–2.62 (m, 2H), 2.13–1.94 (m, 1H), 1.91–1.70 (m, 4H), 1.66–1.41 (m, 4H), 1.32 (s, 9H); ^13^C{^1^H} NMR (100 MHz, CDCl_3_) δ 141.7 (C), 139.8 (CH), 128.8 (CH), 128.7 (CH), 128.6 (CH), 128.4 (CH), 126.1 (CH), 115.6 (CH_2_), 77.3 (C), 64.3 (CH), 56.8 (C), 37.9 (CH_2_), 34.0 (CH_2_), 32.9 (CH_2_), 32.5 (CH_2_), 32.3 (CH_2_), 28.6 (CH_2_), 23.2 (CH_3_), 22.4 (CH_2_); LRMS (EI) *m*/*z* [M^+^(^81^Br)–C_4_H_10_NO_2_S, 12%], 306 [M^+^(^79^Br)–C_4_H_10_NO_2_S, 12%, 238 (53), 205 (10), 182 (14), 164 (39), 157 (21), 134 (22), 117 (83), 91 (100), 67 (13), 57 (55), 55 (32), 41 (19); HRMS (EI-TOF) Calcd for C_17_H_25_NO_2_S [M^+^–C_4_H_9_Br] 307.1606, found 307.1615.

##### (*R*_S_,3*S*,4*R*)-3-Amino-*N*-(*tert*-butanesulfinyl)-1-phenyl-4-vinyldec-9-en-4-ol (**4ag**)

Following the general procedure, compound **4ag** (39.6 mg, 0.10 mmol, 35%) was obtained as a yellow wax; [α]_D_^23^ = −45.7 (*c* = 1.11, CH_2_Cl_2_); *R*_f_ = 0.20 (hexane/EtOAc, 3:1); ^1^H NMR (400 MHz, CDCl_3_) δ 7.33–7.12 (m, 5H), 5.91–5.61 (m, 2H), 5.36–5.14 (m, 2H), 5.02–4.84 (m, 2H), 3.45 (d, *J* = 7.3 Hz, 1H), 3.16–3.02 (m, 1H), 3.00–2.88 (m, 2H), 2.86 (s, 1H), 2.76–2.59 (m, 2H), 2.11–1.94 (m, 4H), 1.89–1.68 (m, 2H), 1.64–1.39 (m, 2H), 1.30 (s, 9H); ^13^C{^1^H} NMR (100 MHz, CDCl_3_) δ 141.7 (C), 140.0 (CH), 139.0 (CH), 128.8 (CH), 128.7 (CH), 128.6 (CH), 128.55 (CH), 128.4 (CH), 126.1 (CH), 115.4 (CH_2_), 114.5 (CH_2_), 77.4 (C), 64.35 (CH), 56.8 (C), 38.1 (CH_2_), 33.8 (CH_2_), 32.6 (CH_2_), 32.4 (CH_2_), 29.45 (CH_2_), 23.2 (CH_3_), 22.7 (CH_2_); LRMS (EI) *m*/*z* 321 (M^+^–C_4_H_8_, 1%), 238 (42), 182 (12), 164 (33), 139 (11), 136 (14), 134 (21), 117 (81), 108 (10), 104 (12), 91 (100), 67 (12), 57 (49), 55 (35), 41 (25); HRMS (EI-TOF) Calcd for C_18_H_27_NO_2_S [M^+^–C_4_H_8_] 321.1766, found 321.1764.

##### (*R*_S_,3*S*,4*S*)-4-Amino-*N*-(*tert*-butanesulfinyl)-3-phenylnona-1,8-dien-3-ol (**4eb**)

Following the general procedure, compound **4eb** (61.1 mg, 0.18 mmol, 61%) was obtained as a yellow wax; [α]_D_^23^ = +11.4 (*c* = 0.92, CH_2_Cl_2_); *R*_f_ = 0.29 (hexane/EtOAc, 3:1); ^1^H NMR (400 MHz, CDCl_3_) δ 7.53–7.29 (m, 5H), 6.12 (ddd, *J* = 17.0, 10.7, 1.4 Hz, 1H), 5.89–5.70 (m, 1H), 5.48 (dd, *J* = 17.0, 1.6 Hz, 1H), 5.19 (dd, *J* = 10.7, 1.6 Hz, 1H), 5.09–4.89 (m, 2H), 4.78 (d, *J* = 1.4 Hz, 1H), 3.74–3.58 (m, 1H), 3.40 (d, *J* = 3.1 Hz, 1H), 2.16–1.94 (m, 2H), 1.97–1.80 (m, 2H), 1.32–1.17 (m, 2H), 1.02 (s, 9H); ^13^C{^1^H} NMR (100 MHz, CDCl_3_) δ 144.8 (C), 140.5 (CH), 138.6 (CH), 128.9 (CH), 127.2 (CH), 125.8 (CH), 114.9 (CH_2_), 114.7 (CH_2_), 79.2 (C), 65.3 (CH), 55.7 (C), 33.6 (CH_2_), 26.3 (CH_2_), 26.0 (CH_2_), 22.7 (CH_3_); LRMS (EI) *m*/*z* 335 (M^+^, < 1%), 202 (39), 146 (44), 133 (59), 115 (10), 105 (23), 81 (28), 70 (15), 55 (44), 43 (100); HRMS (EI-TOF) Calcd for C_19_H_29_NO_2_S [M^+^] 335.1928, found 335.1923.

#### Synthesis of (*R*_S_,1*R*,7*R*)-7-Amino-*N*-(*tert*-butanesulfinyl)-1-phenylcyclohept-2-en-1-ol (**6**) from Amino Alcohol Derivative **3eb**

A solution of compound **3eb** (0.022 g, 0.065 mmol), Hoveyda–Grubbs second generation catalyst (4.34 mg, 0.007 mmol, 10 mol %), and 1,7-octadiene (44 μL, 0.3 mmol) in dry CH_2_Cl_2_ (2.0 mL) was stirred at 40 °C (oil bath) for 17 h. Then the solvent was evaporated (15 Torr). The residue was purified by column chromatography (silica gel, hexane/EtOAc) to give compound **6** (10.1 mg, 0.033 mmol, 55%) as a white solid; mp 64–66 °C (hexane/CH_2_Cl_2_); [α]_D_^23^ = +38.7 (*c* = 0.83, CH_2_Cl_2_); *R*_f_ = 0.20 (hexane/EtOAc, 2:1); ^1^H NMR (400 MHz, CDCl_3_) δ 7.61–7.48 (m, 2H), 7.46–7.21 (m, 3H), 6.13 (ddd, *J* = 12.1, 7.7, 4.3 Hz, 1H), 5.82–5.64 (m, 1H), 4.90 (s, 1H), 3.98–3.80 (m, 2H), 2.29–2.02 (m, 2H), 1.78–1.64 (m, 1H), 1.57–1.42 (m, 3H), 1.28 (s, 9H); ^13^C{^1^H} NMR (100 MHz, CDCl_3_) δ 141.85 (C), 137.5 (CH), 132.0 (CH), 128.6 (CH), 127.9 (CH), 127.25 (CH), 81.1 (C), 65.2 (CH), 56.0 (C), 31.4 (CH_2_), 28.75 (CH_2_), 22.9 (CH_3_), 20.7 (CH_2_); LRMS (EI) *m*/*z* 305 (M^+^, < 1%), 233 (20), 202 (13), 170 (100), 159 (25), 142 (29), 128 (11), 105 (40), 91 (20), 77 (23), 56 (38), 43 (26); HRMS (EI-TOF) Calcd for C_13_H_17_NO_2_S [M^+^–C_4_H_8_] 250.0892, found 250.0890.

##### Synthesis of (2*R*,3*S*)-2-Phenethyl-3-vinylpiperidin-3-ol (**7**) from Amino Alcohol Derivative **3af**

To a solution of compound **3af** (0.012 g, 0.03 mmol) in MeOH (0.5 mL) was added a 2 M solution of HCl in Et_2_O (115.0 μL, 0.23 mmol) at 0 °C. The reaction mixture was stirred at the same temperature for 30 min. After that, a 2 M aqueous solution of NaOH (2.0 mL, 2.0 mmol) was added to the reaction mixture at 0 °C, and after 10 min, it was extracted with CH_2_Cl_2_ (4 × 5 mL), the combined organic phases dried over anhydrous MgSO_4_, and the solvents evaporated (15 Torr). To a solution of the resulting residue in CH_2_Cl_2_ (2.0 mL) was added a 2 M aqueous solution of NaOH (2.0 mL, 4.0 mmol), and the reaction mixture was stirred at 23 °C for 15 h. After that, the aqueous layer was extracted with CH_2_Cl_2_ (5 × 5 mL), and the combined organic layers were dried over anhydrous MgSO_4_, and the solvents evaporated (15 Torr). The residue was purified by column chromatography (silica gel, hexane/EtOAc) to yield pure compound **7** (6.5 mg, 0.028 mmol, 93%) as a white solid; mp 39–41 °C (hexane/CH_2_Cl_2_); [α]_D_^23^ = +8.6 (*c* = 0.45 CH_2_Cl_2_); *R*_f_ = 0.67 (hexane/EtOAc, 1:1); ^1^H NMR (400 MHz, CDCl_3_) δ 7.40–7.07 (m, 5H), 5.72 (dd, *J* = 17.1, 10.6 Hz, 1H), 5.37–5.07 (m, 2H), 3.95–3.71 (m, 2H), 2.94 (ddd, *J* = 14.6, 10.3, 4.8 Hz, 1H), 2.75–2.47 (m, 2H), 1.95–1.73 (m, 4H), 1.71–1.63 (m, 2H), 1.25 (s, 1H); ^13^C{^1^H} NMR (100 MHz, CDCl_3_) δ 142.45 (C), 138.5 (CH), 128.5 (CH), 128.4 (CH), 125.8 (CH), 115.4 (CH_2_), 88.5 (C), 67.6 (CH_2_), 59.1 (CH), 35.2 (CH_2_), 34.0 (CH_2_), 33.5 (CH_2_), 25.5 (CH_2_); LRMS (EI) *m*/*z* 231 (M^+^, < 1%), 134 (100), 117 (35), 91 (94), 55 (25) 43 (15); HRMS (EI-TOF) Calcd for C_15_H_19_N [M^+^–H_2_O] 214.1595, found 214.1587.

##### Synthesis of (*R*_S_,2*R*,3*S*,*E*)-2-Amino-*N*-(*tert*-butanesulfinyl)-1-*O*-(*tert*-butyldimethylsilyl)-octadec-4-ene-1,3-diol (**8**) from Amino Diol Derivative **3da**

To a solution of allylic amino alcohol derivative **3da** (67.0 mg, 0.2 mmol), 1-pentadecene (84.0 mg, 108.4 μL, 0.4 mmol), and 1,7-octadiene (88.0 mg, 108.0 μL, 0.8 mmol) in anhydrous CH_2_Cl_2_ (1.0 mL) was added Hoveyda–Grubbs II catalyst (12.5 mg, 0.02 mmol). This mixture was stirred at 45 °C (oil bath) for 3 h. After that, the solvents evaporated (15 Torr). The residue was purified by column chromatography (silica gel, hexane/EtOAc) to yield pure compound **8** (62.0 mg, 1.20 mmol, 60%) as a colorless oil; [α]_D_^23^ = −42.7 (*c* = 0.97 CH_2_Cl_2_); *R*_f_ = 0.48 (hexane/EtOAc, 2:1); ^1^H NMR (300 MHz, CDCl_3_) δ 5.81 (dtd, *J* = 15.4, 6.8, 1.5 Hz, 1H), 5.50 (ddt, *J* = 15.5, 5.2, 1.4 Hz, 1H), 4.42 (br s, 1H), 4.02 (d, *J* = 9.7 Hz, 1H), 3.82–3.70 (m, 2H), 3.58 (dd, *J* = 10.2, 6.5 Hz, 1H), 3.52–3.42 (m, 1H), 2.08 (dd, *J* = 7.8, 6.5 Hz, 2H), 1.27 (s, 22H), 1.25 (s, 9H), 0.91 (s, 9H), 0.90 (t, *J* = 6.6 Hz, 3H), 0.09 (s, 3H), 0.08 (s, 3H); ^13^C{^1^H} NMR (75 MHz, CDCl_3_) δ 134.5 (CH), 127.7 (CH), 72.4 (CH), 64.3 (CH_2_), 62.5 (CH), 55.8 (C), 32.5 (CH_2_), 31.9 (CH_2_), 29.7 (CH_2_), 29.6 (CH_2_), 29.6 (CH_2_), 29.5 (CH_2_), 29.4 (CH_2_), 29.3 (CH_2_), 29.3 (CH_2_), 25.8 (CH_3_), 22.7(CH_3_), 22.6 (CH_2_), 14.1 (CH_3_), −5.5 (CH_3_), −5.6 (CH_3_); LRMS (EI) *m*/*z* 517 (M^+^, < 1%), 460 (7), 345 (19), 323 (100), 278 (89), 239 (16), 203 (16), 174 (65), 133 (11), 116 (47), 105 (10), 89 (57), 75 (78), 57 (88), 41 (32); HRMS (EI-TOF) Calcd for C_24_H_50_NO_3_SSi [M^+^–C_4_H_9_] 460.3281, found 460.3286.

## Data Availability

The data underlying this study are available in the published article and its Supporting Information.

## Supporting Information Available

Experimental procedures and characterization data for compounds **1** and **2**. Experimental epimerization conditions of the sulfur atom of amino alcohol derivative **3ae**. Copies of ^1^H, ^13^C NMR, and DEPT spectra for all the reported compounds (**1**, **2**, **3**, **4**, **6**, **7**, and **8**), and X-ray structures of compounds **3ab** and **3ai**. (PDF)
