# Supplementary material for: Synthesis of Vicinal anti-Amino Alcohols from N-tert-Butanesulfinyl Aldimines and Cyclopropanols
Source: J Org Chem. 2024 Apr 13;89(9):6193–204. doi: 10.1021/acs.joc.4c00198 (PMC11077494; doi:10.1021/acs.joc.4c00198)
Supplement: Supplementary file 1 — jo4c00198_si_001.pdf [file jo4c00198_si_001.pdf]

Supporting Information for

Synthesis of Vicinal *anti*-Amino Alcohols from *N-tert*-Butanesulfinyl Aldimines and Cyclopropanols

Sandra Hernández-Ibáñez,[a,b,c] *Juan F. Ortuño,*[a,b,c] *Ana Sirvent,*[a,b,c] *Carmen Nájera,*[c] *José Miguel Sansano,*[a,b,c] *Miguel Yus,*[c] *and Francisco Foubelo*[a,b,c]*

[a] Departamento de Química Orgánica, Facultad de Ciencias, Universidad de Alicante, Apdo. 99, 03080 Alicante (Spain)

[b] Instituto de Síntesis Orgánica (ISO), Universidad de Alicante, Apdo. 99, 03080 Alicante (Spain)

[c] Centro de Innovación en Química Avanzada (ORFEO-CINQA), Universidad de Alicante, Apdo. 99, 03080 Alicante (Spain)

Contents:

Procedure and characterization data for chiral *N-tert*-butanesulfinyl imines **1**…………………S2-S3

Procedure and characterization data for cyclopropanols **2**……………………...………………S4-S6

Epimerization of the sulfur atom of amino alcohol derivative **3ae**……………………...……….…S7

^{1}H-NMR, ^{13}C-NMR, DEPT spectra of compounds **1**, **2**, **3**, **4**, **6**, **7** and **8**……..............……….....S8-S42

X-ray structure of compounds **3ab** and **3ai**…..……………….……………….……..………...S43-S44

General Procedure for the Synthesis of *N*-*tert*-Butanesulfinyl Imines 1a-c and 1e from the Corresponding Aldehydes and (*R*)-*tert*-Butanesulfinamide. To a solution of (*R*)-*tert*-butanesulfinamide (0.605 g, 5.0 mmol) and the corresponding aldehyde (5.5 mmol) in dry THF (20 mL) under argon at 23 °C was slowly added $Ti(OEt)_4$ (2.2281 g, 2.095 mL, 10.0 mmol). The reaction mixture was stirred for 12 h at the same temperature. The resulting mixture was hydrolyzed with brine (30 mL), extracted with AcOEt (3 × 15 mL), dried with anhydrous $MgSO_4$, and the solvents evaporated (15 Torr). The residue was purified by column chromatography (silica gel, hexane/AcOEt) to yield pure compounds **1**. Yields, physical and spectroscopic data follow.

(*R*)-*N*-(*tert*-Butanesulfinyl)-*N*-(3-phenylpropyliden)amine (1a).[1] Following the general procedure, compound **1a** (1.04 g, 4.40 mmol, 88%) was obtained from (*R*)-*tert*-butanesulfinamide and 3-phenylpropanal as a colorless liquid; $[\alpha]_D^{23}$ = –138 (*c* = 1.53, CH_2Cl_2); R_f = 0.57 (hexane/EtOAc, 3:1); ^1H NMR (300 MHz, $CDCl_3$) δ 8.11 (t, *J* = 4.1 Hz, 1 H), 7.26–7.31 (m, 2 H), 7.22–7.19 (m, 3 H), 3.00–2.95 (m, 2 H), 2.89-2.82 (m, 2 H), 1.12 (s, 9 H); $^{13}C\{^1H\}$ NMR (75 MHz, $CDCl_3$) δ 168.5 (CH), 140.2 (C), 128.5 (CH), 128.3 (CH), 126.2 (CH), 56.5 (C), 37.4 (CH_2), 31.3 (CH_2), 22.2 (CH_3); LRMS (EI) *m/z* 131 (M^+–$C_4H_{10}SO$, 34%), 92 (11), 91 (100), 65 (19), 51 (10).

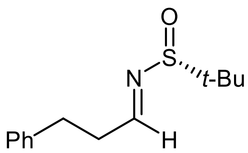

1a

(*R*)-*N*-Benzyliden-*N*-(*tert*-butanesulfinyl)amine (1b).[2] Following the general procedure, compound **1b** (0.90 g, 4.3 mmol, 86%) was obtained from (*R*)-*tert*-butanesulfinamide and isobutyraldehyde as a colorless liquid; $[\alpha]_D^{23}$ = –98 (*c* = 0.93, CH_2Cl_2); R_f = 0.65 (hexane/EtOAc, 3:1); ^1H NMR (300 MHz, $CDCl_3$) δ 8.59 (s, 1 H), 7.88–7.85 (m, 2 H), 7.53-7.45 (m, 3 H), 1.27 (s, 9 H); $^{13}C\{^1H\}$ NMR (75 MHz, $CDCl_3$) δ = 162.7 (CH), 134.0 (C), 132.4 (CH), 129.4 (CH), 128.9 (CH), 57.7 (C), 22.6 (CH_3); LRMS (EI) *m/z* 103 (M^+–$C_4H_{10}SO$, 100%), 76 (31), 50 (19).

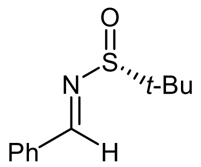

1b

(*R*)-*N*-(*tert*-Butanesulfinyl)-*N*-(2-methylpropyliden)amine (1c).[2] Following the general procedure, compound **1c** (0.79 g, 4.5 mmol, 90%) was obtained from (*R*)-*tert*-butanesulfinamide and isobutyraldehyde as a colorless liquid; $[\alpha]_D^{23}$ = –232 (*c* = 0.49, CH_2Cl_2); R_f = 0.70 (hexane/EtOAc, 3:1); ^1H NMR (300 MHz, $CDCl_3$) δ 7.95 (d, *J* = 4.3 Hz, 1 H), 2.71–2.66 (m, 1 H), 1.16 (s, 9 H), 1.14 (d, *J* = 7.0 Hz, 3 H), 1.13 (d, *J* = 7.0 Hz, 3 H); $^{13}C\{^1H\}$ NMR (75 MHz, $CDCl_3$) δ 173.5 (CH), 56.4 (C), 34.8 (CH), 22.2 (CH_3), 18.8 (CH_3); LRMS (EI) *m/z* 175 (M^+, 2%), 119 (20), 57 (100), 56 (52), 55 (10).

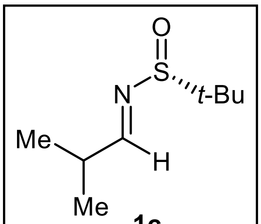

1c

(*R*)-*N*-(*tert*-Butanesulfinyl)-*N*-(hex-5-enyliden)amine (1e).[3] Following the general procedure,

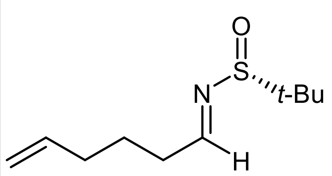
1e

compound **1e** (0.78 g, 3.9 mmol, 78%) was obtained from (*R*)-*tert*-butanesulfinamide and 5-hexenal as a colorless oil; $[\alpha]_D^{23}$ = +26.9 (*c* = 0.99, CH_2Cl_2); R_f = 0.61 (hexane/EtOAc 3:1); ^{1}H NMR (400 MHz, $CDCl_3$) δ 8.08 (t, J = 4.6 Hz, 1H), 5.91–5.65 (m, 1H), 5.17–4.83 (m, 2H), 2.54 (td, J = 7.5, 4.6 Hz, 2H), 2.21–2.06 (m, 2H), 1.75 (q, J = 7.5 Hz, 2H), 1.20 (s, 9H); $^{13}C\{^1H\}$ NMR (100 MHz, $CDCl_3$) δ 169.4 (CH), 137.8 (CH), 115.5 (CH_2), 56.6 (C), 35.5 (CH_2), 33.2 (CH_2), 24.7 (CH_2), 22.4 (CH_3); LRMS (EI) *m/z* 201 (M^+, <1%), 145 (20), 97 (11), 81 (27), 57 (100), 41 (33); HRMS (EI-TOF) Calcd for $C_{10}H_{19}NOS$ [M^+] 201.1187, found 201.1197.

Synthesis of (*R*)-*N*-(*tert*-Butanesulfinyl)-2-(*tert*-butyldimethylsilyloxy)ethylidenamine (1d) from Glycerol and (*R*)-*tert*-Butanesulfinamide.[4]

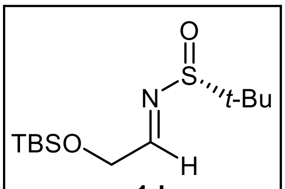
1d

To a solution of glycerol (4.37 g, 3.46 mL, 47.4 mmol), imidazole (4.08 g, 60.0 mmol), and DMF (22.85 g, 24.2 mL, 312.6 mmol) in CH_2Cl_2 (40 mL), a solution of TBSCl (3.255 g, 3.74 mL, 20.0 mmol) in CH_2Cl_2 (15 mL) was added dropwise at -18 ºC. The mixture was stirred for 1 h at room temperature. Then, it was hydrolyzed with water (100 mL), the aqueous phase was extracted with CH_2Cl_2 (3 × 25 mL), and the organic phase was washed with water (50 mL) and brine (40 mL), dried over anhydrous MgSO4, and the solvent was evaporated (15 Torr), yielding 3.34 g (16.0 mmol) of a colorless oil, which was identified as TBS-glycerol. This reaction crude was dissolved in CH_2Cl_2 (40 mL), and a solution of $NaIO_4$ (5.0 g, 24.0 mmol) in water (40 mL) was added in one portion. After stirring at room temperature for 3 h, the phases were separated, and the organic phase was washed with water (40 mL) and brine (30 mL), dried with anhydrous MgSO4, and the solvent was evaporated (15 Torr), obtaining 2.262 g (13.0 mmol) of colorless oil identified as *O*-TBS protected hydroxyacetaldehyde. The resulting reaction crude was added to a suspension of anhydrous $CuSO_4$ (32.5 mmol) and (*R*)-*tert*-butanesulfinamide (1.73 g, 14.3 mmol) in anhydrous CH_2Cl_2 (25 mL), and stirred at room temperature for 24 h. The resulting suspension was filtered through a plug of Celite, and the filter cake was washed with EtOAc (4 × 20 mL). After evaporation of the solvent (15 Torr), the residue was purified by column chromatography (silica gel, hexane/EtOAc) to yield pure compound **1d** (2.09 g, 7.54 mmol, 58%) as a colorless oil; $[\alpha]_D^{23}$ = –180.4 (*c* = 1.10, CH_2Cl_2); R_f = 0.61 (hexane/EtOAc 3:1); ^{1}H NMR (300 MHz, $CDCl_3$) δ 8.06 (t, J = 3.0 Hz, 1H), 4.55 (d, J = 3.0 Hz, 2H), 1.21 (s, 9H), 0.93 (s, 9H), 0.11 (s, 6H); $^{13}C\{^1H\}$ NMR (100 MHz, $CDCl_3$) δ 168.7 (CH),

65.6 (CH_2), 56.8 (C), 25.7 (CH_3), 22.3 (CH_3), 18.3 (C), –5.29 (CH_3), –5.31 (CH_3); LRMS (EI) *m/z* 221 (M^+–C_4H_8, 6%), 174 (16), 164 (25), 149 (26), 111 (15), 97 (20), 71 (24), 57 (71), 43 (100).

General Procedure for the Synthesis of Cyclopropanols 2. To a solution of the corresponding methyl or ethyl ester (25.0 mmol) and Ti(O*i*-Pr)$_4$ (0.71 g, 0.73 mL, 2.5 mmol) in Et_2O (80 mL) was slowly added over a period of 1 h a 1.0 M solution of ethylmagnesium bromide in ether (60 mL, 60.0 mmol), at 15-20 ºC. The stirring is continued for an additional 10 min. The resulting mixture is then carefully poured into a 10% aqueous H_2SO_4 solution (250 mL), which has been pre-cooled to 5 °C, extracted with AcOEt (3 × 30 mL), the organic phase washed with water (3 × 30 mL), dried with anhydrous $MgSO_4$, and the solvents evaporated (15 Torr). The residue was purified by column chromatography (silica gel, hexane/AcOEt) to yield pure compounds **2**. Yields, physical and spectroscopic data follow.

1-Phenylcyclopropanol (2b).[5] Following the general procedure, compound **2b** (2.88 g, 21.5 mmol, 86%) was obtained from ethyl benzoate as a colorless oil; R_f = 0.33 (hexane/EtOAc 6:1); ^{1}H NMR (400 MHz, $CDCl_3$) δ 7.45–7.19 (m, 6H), 2.60 (s, 1H), 1.34–1.24 (m, 2H), 1.13–0.99 (m, 2H); $^{13}C\{^1H\}$ NMR (100 MHz, $CDCl_3$) δ 144.4 (C), 128.52 (CH), 128.5 (CH), 126.5 (CH), 124.5 (CH), 56.7 (C), 17.95 (CH_2); LRMS (EI) *m/z* 134 (M^+, 20%), 133 (19), 115 (45), 105 (78), 91 (100), 79 (80), 78 (63), 77 (74), 55 (14), 51 (35).

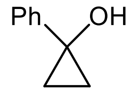

2b

1-Decylcyclopropanol (2c).[6] Following the general procedure, compound **2c** (3.07 g, 15.5 mmol, 62%) was obtained from methyl undecanoate as a white solid; mp 50–51 °C (hexane/CH_2Cl_2), R_f = 0.53 (hexane/EtOAc 6:1); ^{1}H NMR (400 MHz, $CDCl_3$) δ 2.03 (s, 1H), 1.38–1.19 (m, 16H), 0.94–0.84 (m, 3H), 0.78–0.69 (m, 2H), 0.49–0.39 (m, 2H); $^{13}C\{^1H\}$ NMR (100 MHz, $CDCl_3$) δ 56.0 (C), 38.4 (CH_2), 32.05 (CH_2), 29.8 (CH_2), 29.8 (CH_2), 29.5 (CH_2), 26.1 (CH_2), 22.8 (CH_2), 14.25 (CH_3), 13.6 (CH_2); LRMS (EI) *m/z* 198 (M^+, 3%), 169 (59), 95 (20), 85 (85), 73 (28), 72 (100), 57 (63), 55 (27).

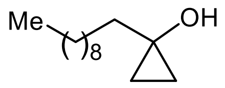

2c

1-(6-Bromohexyl)cyclopropanol (2d). Following the general procedure, compound **2d** (3.98 g, 18 mmol, 72%) was obtained from ethyl 7-bromoheptanoate as a colorless oil; R_f = 0.28 (hexane/EtOAc 6:1); ^{1}H NMR (400 MHz, $CDCl_3$) δ 3.34 (t, J = 6.8 Hz, 2H), 1.97 (s, 1H), 1.87–1.69 (m, 2H), 1.53–1.20 (m, 8H), 0.74–0.60 (m, 2H), 0.45–0.23 (m, 2H); $^{13}C\{^1H\}$ NMR (100 MHz, $CDCl_3$) δ 55.8 (C), 38.3 (CH_2), 34.1 (CH_2), 32.8 (CH_2), 28.9 (CH_2), 28.3 (CH_2), 25.85 (CH_2), 13.6 (CH_2); LRMS (EI) *m/z* 222 [M^+ (^{81}Br), 6%], 220 [M^+ (^{79}Br), 7%], 193 (35), 191 (38), 141.1 (50), 123.10

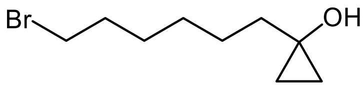

2d

(12), 88 (12), 83 (69), 73 (11), 72 (100), 57 (89), 55 (59); HRMS (EI-TOF) Calcd for $C_9H_{17}{}^{79}BrO$ [M$^+$] 220.0463, found 220.0458.

1-(5-Bromopentyl)cyclopropanol (2e).[7] Following the general procedure, compound **2e** (3.52 g, 17 mmol, 68%) was obtained from ethyl 6-bromohexanoate as a colorless oil: R_f = 0.26 (hexane/EtOAc 6:1); ^{1}H NMR (400 MHz, $CDCl_3$) δ 3.43 (t, J = 6.8 Hz, 2H), 1.96–1.81 (m, 2H), 1.60–1.53 (m, 6H), 0.79–0.70 (m, 2H), 0.52–0.32 (m, 2H); ^{13}C{^{1}H} NMR (100 MHz, $CDCl_3$) δ 55.9 (C), 38.3 (CH_2), 34.1 (CH_2), 32.9 (CH_2), 28.3 (CH_2), 25.3 (CH_2), 13.8 (CH_2); LRMS (EI) *m/z* 208 [M$^+$ (^{81}Br), 2%], 206 [M$^+$ (^{79}Br), 2%], 179 (10), 177 (11), 85 (20), 70 (10), 69 (34), 57 (100), 53 (11).

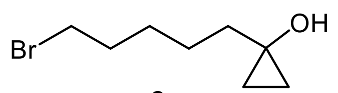

2e

1-(3-Bromopropyl)cyclopropanol (2f).[8] Following the general procedure, compound **2f** (2.51 g, 14 mmol, 56 %) was obtained from ethyl 4-bromobutanoate as a colorless oil; R_f = 0.25 (hexane/EtOAc 6:1); ^{1}H NMR (400 MHz, $CDCl_3$) δ 3.51 (t, J = 6.7 Hz, 2H), 2.18–2.06 (m, 2H), 1.75–1.63 (m, 2H), 0.80–0.69 (m, 2H), 0.54–0.42 (m, 2H); ^{13}C{^{1}H} NMR (100 MHz, $CDCl_3$) δ 55.05 (C), 36.8 (CH_2), 34.1 (CH_2), 29.5 (CH_2), 13.6 (CH_2); LRMS (EI) *m/z* 180 [M$^+$ (^{81}Br), 6%], 178 [M$^+$ (^{81}Br), 5%], 151 (11), 149 (10), 79 (14), 72 (100), 57 (65), 55 (17), 53 (20).

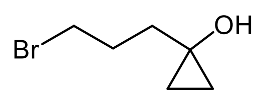

2f

1-(Hex-5-en-1-yl)cyclopropanol (2g). Following the general procedure, compound **2g** (2.55 g, 18.2 mmol, 73 %) was obtained from ethyl hept-6-enoate as a colorless oil; R_f = 0.40 (hexane/EtOAc 6:1); ^{1}H NMR (400 MHz, $CDCl_3$) δ 5.91–5.74 (m, 1H), 5.07–4.89 (m, 2H), 2.15 (s, 1H), 2.11–2.00 (m, 2H), 1.59–1.35 (m, 6H), 0.78–0.66 (m, 2H), 0.49–0.36 (m, 2H); ^{13}C{^{1}H} NMR (100 MHz, $CDCl_3$) δ 139.0 (CH), 114.5 (CH_2), 55.8 (C), 38.2 (CH_2), 33.9 (CH_2), 29.0 (CH_2), 25.5 (CH_2), 13.55 (CH_2); LRMS (EI) *m/z* 140 (M$^+$, 8%), 111 (20), 85 (40), 83 (44), 72 (45), 69 (31), 68 (56), 67 (30), 57 (100), 55 (77), 53 (25); HRMS (EI-TOF) Calcd for $C_{19}H_{16}O$ [M$^+$] 140.1201, found 140.1201.

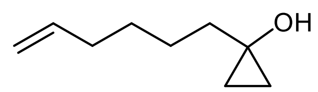

2g

1-Benzhydrylcyclopropanol (2h).[9] Following the general procedure, compound **2h** (3.76 g, 16.7 mmol, 67 %) was obtained from methyl isobutyrate as a colorless oil; R_f = 0.45 (hexane/EtOAc 6:1); ^{1}H NMR (400 MHz, $CDCl_3$) δ 7.43–7.13 (m, 10H), 2.04 (s, 1H), 1.00–0.85 (m, 2H), 0.72–0.60 (m, 2H); ^{13}C{^{1}H} NMR (100 MHz; $CDCl_3$) δ 142.1 (C), 129.45 (CH), 128.55 (CH), 126.8 (CH), 58.1 (C), 57.4 (CH), 13.5 (CH_2); LRMS (EI) *m/z* 224 (M$^+$, 16%), 206 (43), 167 (100), 165 (55), 152 (27), 133 (14), 118 (11), 105 (12), 91 (14).

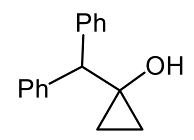

2h

1-(2-Bromophenyl)cyclopropanol (2i).[10] Following the general procedure, compound **2i** (2.93 g, 13.7 mmol, 55 %) was obtained from methyl 2-bromobenzoate as a white solid; mp 51–53 °C (hexane/CH_2Cl_2); R_f = 0.24 (hexane/EtOAc 6:1); ^{1}H NMR (400 MHz, $CDCl_3$) δ 7.62–7.54 (m, 1H), 7.42 (dd, J = 7.6, 1.8 Hz, 1H), 7.30 (dd, J = 7.5, 1.4 Hz, 1H), 7.21–7.08 (m, 1H), 3.03 (s, 1H), 1.37–1.16 (m, 2H), 1.05–0.87 (m, 2H); ^{13}C{^{1}H} NMR (100 MHz, $CDCl_3$) δ 141.05 (C), 133.1 (CH), 130.8 (CH), 129.6 (CH), 127.6 (CH), 58.5 (C), 14.9 (CH_2); LRMS (EI) *m/z* 213 [M^+ (^{81}Br), 27%], 211 [M^+ (^{79}Br), 2%], 185 (33), 183 (32), 157 (15), 155 (13), 133 (100), 132 (18), 105 (22), 77 (37), 55 (20), 51 (20).

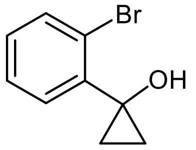

2i

References

1. Schenkel, L. B.; Ellman, J. A. Self-Condensation of *N-tert*-Butanesulfinyl Aldimines: Application to the Rapid Asymmetric Synthesis of Biologically Important Amine-Containing Compounds. *Org. Lett.* **2004**, *6*, 3621−3624.

12. Liu, G.; Cogan, D. A.; Owens, T. D.; Tang, T. P.; Ellman, J. A. Synthesis of Enantiomerically Pure *N-tert*-Butanesulfinyl Imines (*tert*-Butanesulfinimines) by the Direct Condensation of *tert*-Butanesulfinamide with Aldehydes and Ketones. *J. Org. Chem.* **1999**, *64*, 1278−1284.

3. Wang, J.; Li, W.; Liu, L.; Wang, B.; Zhou, Y.; Huang, S.; Wang, X. A unified and straightforward total synthesis of (+)-porantheridine and (−)-6-*epi*-porantheridine. *Org. Chem. Front.* **2019**, *6*, 1599−1602.

4. Ferreira, F.; Botuha, C.; Chemla, F.; Pérez-Luna, A. Expeditious Synthesis of a Common IntermediateofL-1-Deoxyallonojirimycin andL-1-Deoxymannojirimycin. *J. Org. Chem.* **2009**, *74*, 2238−2241.

5. Zhang, X.; Yang, T. M.: Hu, L. M.; Hu, X. H. Stereoselective Iron-Catalyzed Alkylation of Enamides with Cyclopropanols via Oxidative C(sp^2)–H Functionalization. *Org. Lett.* **2022**, *47*, 8677−8682.

6. Yang, J.; Shen, Y.; Jie Lim, Y.; Yoshikai, N. Divergent ring-opening coupling between cyclopropanols and alkynes under cobalt catalysis. *Chem. Sci.* **2018**, *9*, 6928−6934.

7. Vellakkaran, M.; Kim, T.; Hong, S. Visible-Light-Induced C4-Selective Functionalization of Pyridinium Salts with Cyclopropanols. *Angew. Chem. Int. Ed.* **2022**, *61*, e202113658.

8. Liu, J.; Xu, E.; Jiang, J.; Huang, Z.; Zheng, L.; Liu, Z.-Q. Copper-mediated tandem ring-opening/cyclization reactions of cyclopropanols with aryldiazonium salts: synthesis of N-arylpyrazoles. *Chem. Commun.* **2020**, *56*, 2202−2205.

9. He, X.-P.; Shu, Y.-J.; Dai, J.-J.; Zhang, W.-M.; Feng, Y.-S.; Xu, H.-J. Copper-catalysed ring-opening trifluoromethylation of cyclopropanols. *Org. Biomol. Chem.* **2015**, *13*, 7159−7163.

10. Paul, T.; Basak, S.; Punniyamurthy, T. Weak Chelation-Assisted C4-Selective Alkylation of Indoles with Cyclopropanols via Sequential C–H/C–C Bond Activation. *Org. Lett.* **2022**, *24*, 6000−6005.

Epimerization of the Sulfur Atom of Amino Alcohol Derivative 3ae. To a solution of amino alcohol derivative **3ae** (22.0 mg, 0.05 mmol) in CH_2Cl_2 (2 mL) was added a 2 M solution of HCl in Et_2O (0.25 mL, 0.5 mmol) at 0 ºC. The reaction mixture was stirred at the same temperature for 30 min. After that, Et_3N (50.5 mg, 0. 07 mL, 0.5 mmol) was added dropwise and the reaction mixture was stirred at room temperature for 1 h. The reaction mixture was diluted with CH_2Cl_2 (5 mL) and washed successively with a saturated $NaHCO_3$ aqueous solution (5 mL) and brine (5 mL). The organic phase was dried with anhydrous $MgSO_4$, and the solvents evaporated (15 Torr). The residue (20.0 mg) was a mixture of starting amino alcohol derivative **3ae** and its epimer at the sulfur atom of the sulfinyl group. We analyzed the ^1H NMR spectrum of the crude reaction mixture and identified two distinct sets of signals: one corresponding to the starting *anti* isomer **3ae** and another set perfectly matching the signals of compound **4ae**. This unequivocally confirmed the *anti* relative configuration of amino alcohol derivatives **4**, as the ^1H NMR spectra of the epimer at the sulfur atom of **3ae** (*ent*-**4ae**) and **4ae** were entirely identical. Furthermore, a simple TLC experiment revealed identical R_f values for **4ae** and the epimerized product of **3ae** (*ent*-**4ae**).

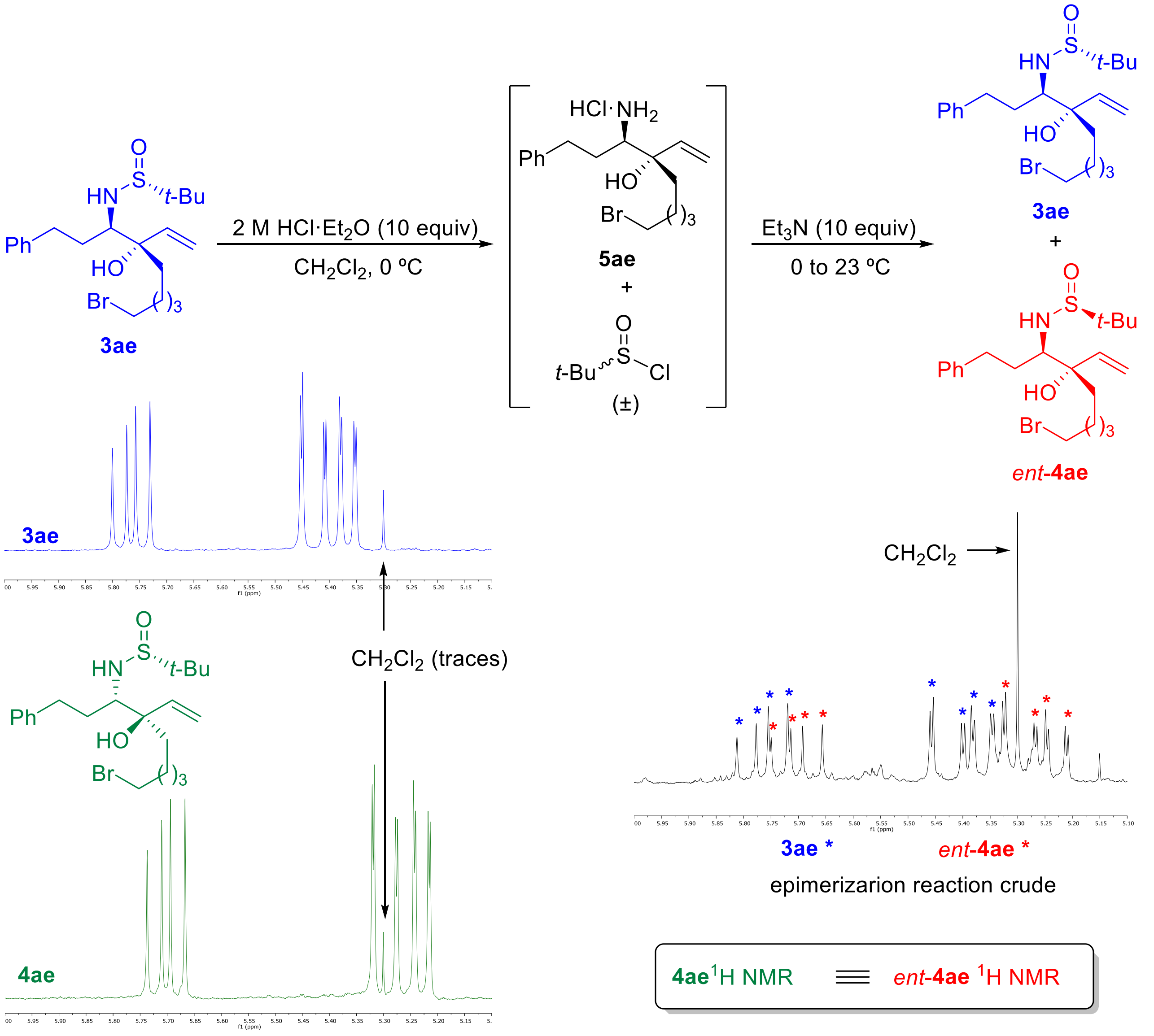

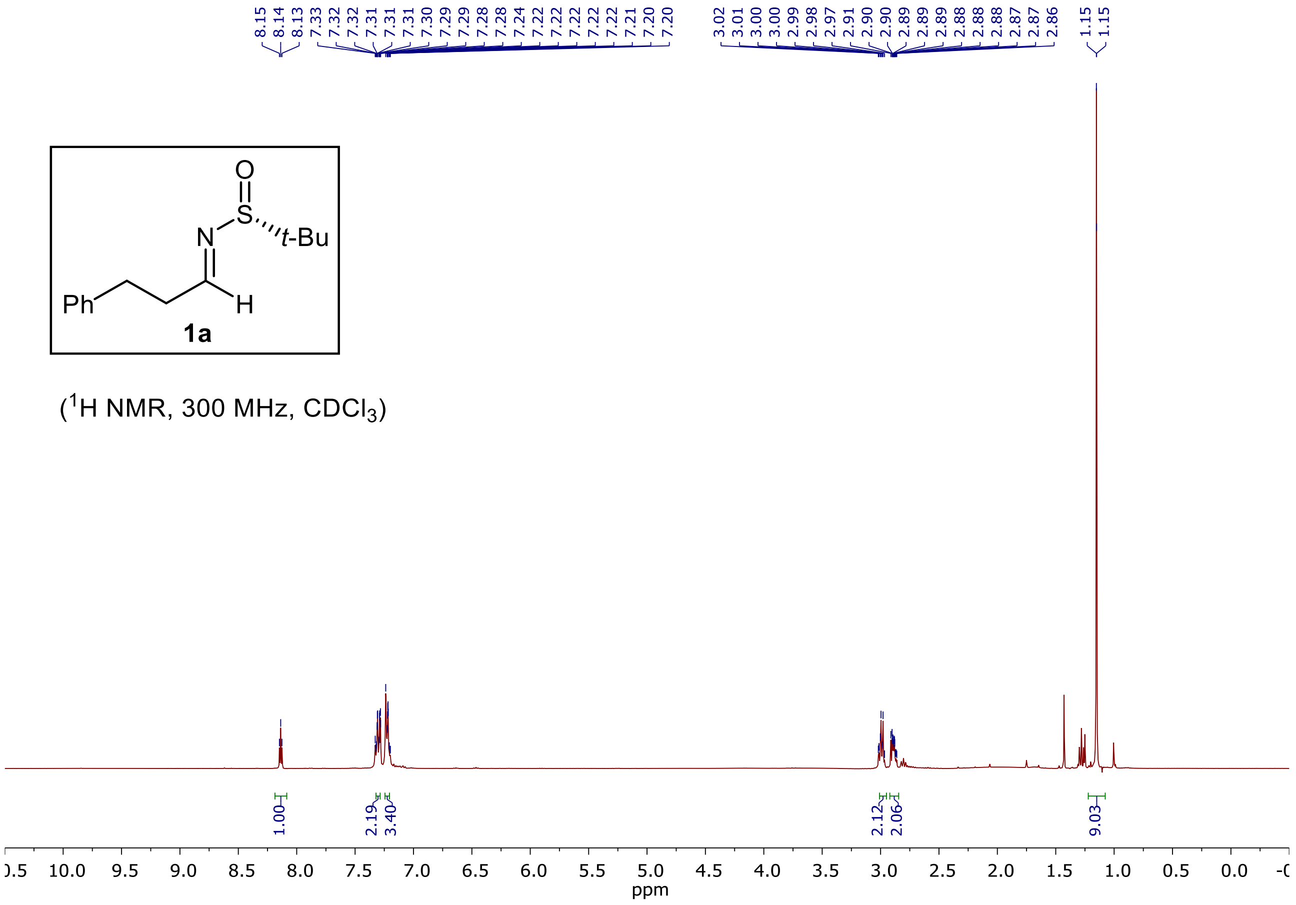
8.15
8.14
8.13
7.33
7.32
7.32
7.31
7.31
7.31
7.30
7.29
7.29
7.28
7.28
7.24
7.22
7.22
7.22
7.22
7.22
7.21
7.20
7.20
3.02
3.01
3.00
3.00
2.99
2.98
2.97
2.91
2.90
2.90
2.89
2.89
2.89
2.88
2.88
2.88
2.87
2.87
2.86
1.15
1.15
O
S
N
t-Bu
Ph
H
1a
(^{1}H NMR, 300 MHz, CDCl$_3$)
1.00
2.19
3.40
2.12
2.06
9.03
ppm

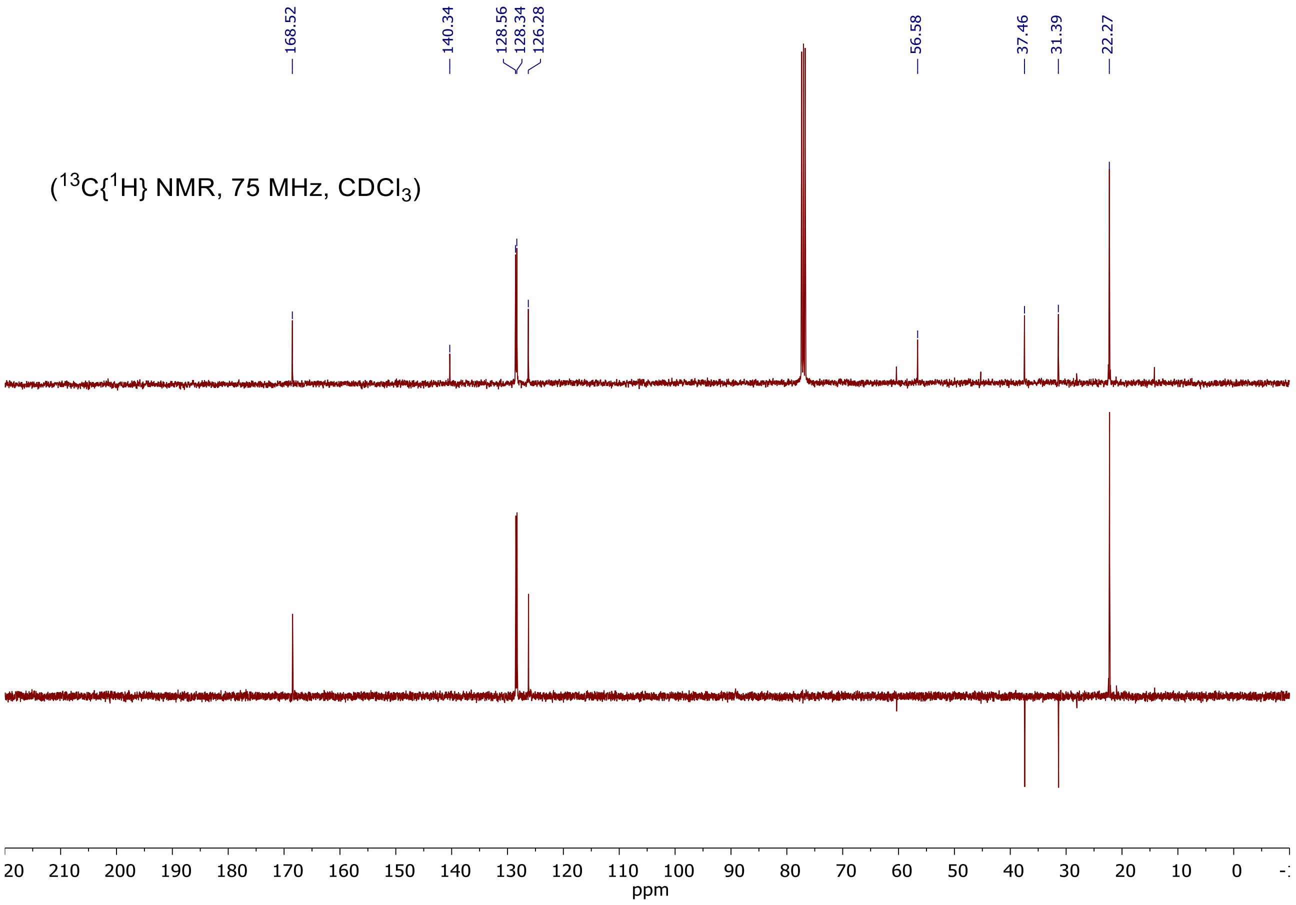
168.52
140.34
128.56
128.34
126.28
56.58
37.46
31.39
22.27
(^{13}C{^{1}H} NMR, 75 MHz, CDCl$_3$)
ppm

1b

(${}^{1}H$ NMR, 300 MHz, $CDCl_3$)

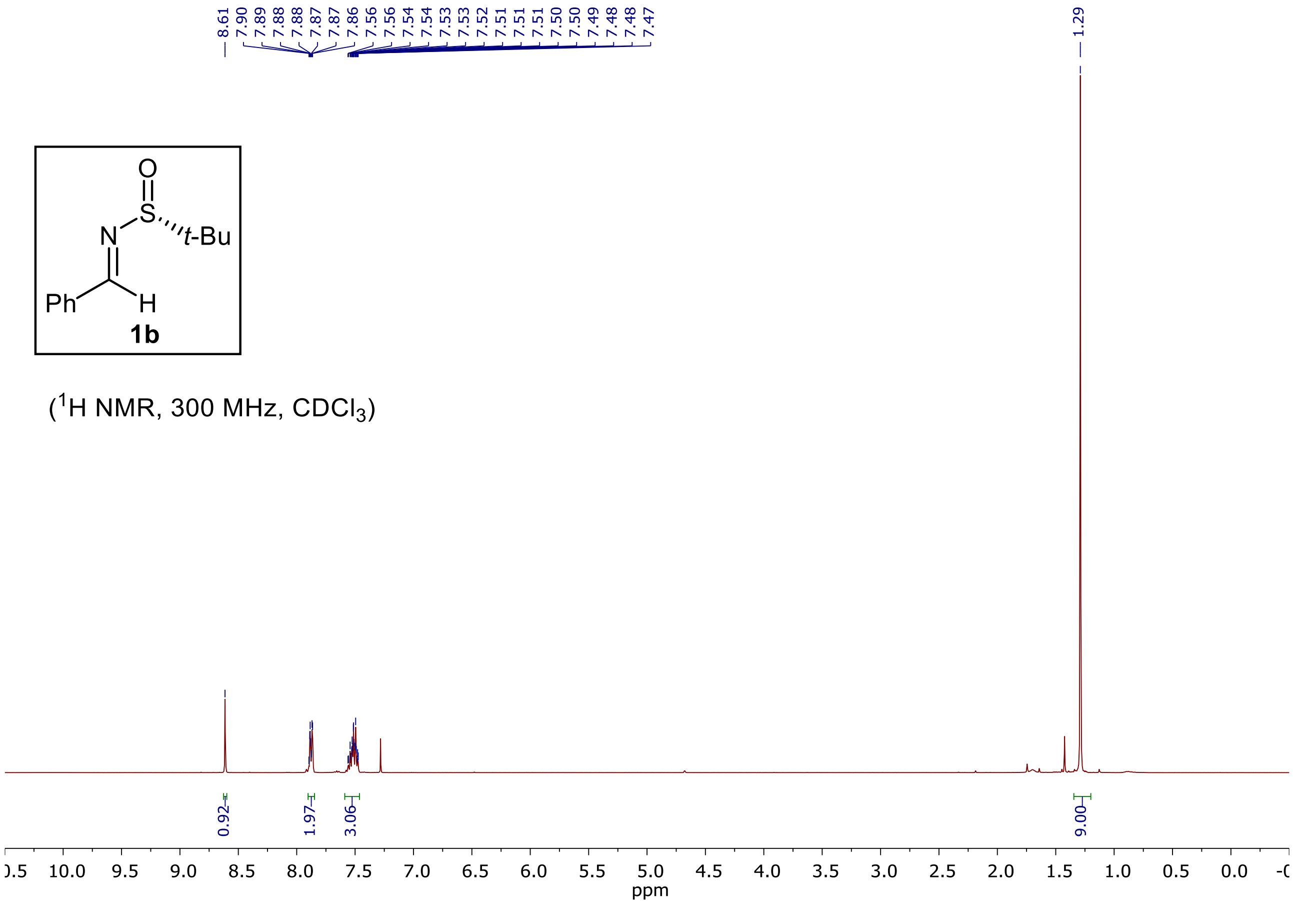

(${}^{13}C\{{}^{1}H\}$ NMR, 75 MHz, $CDCl_3$)

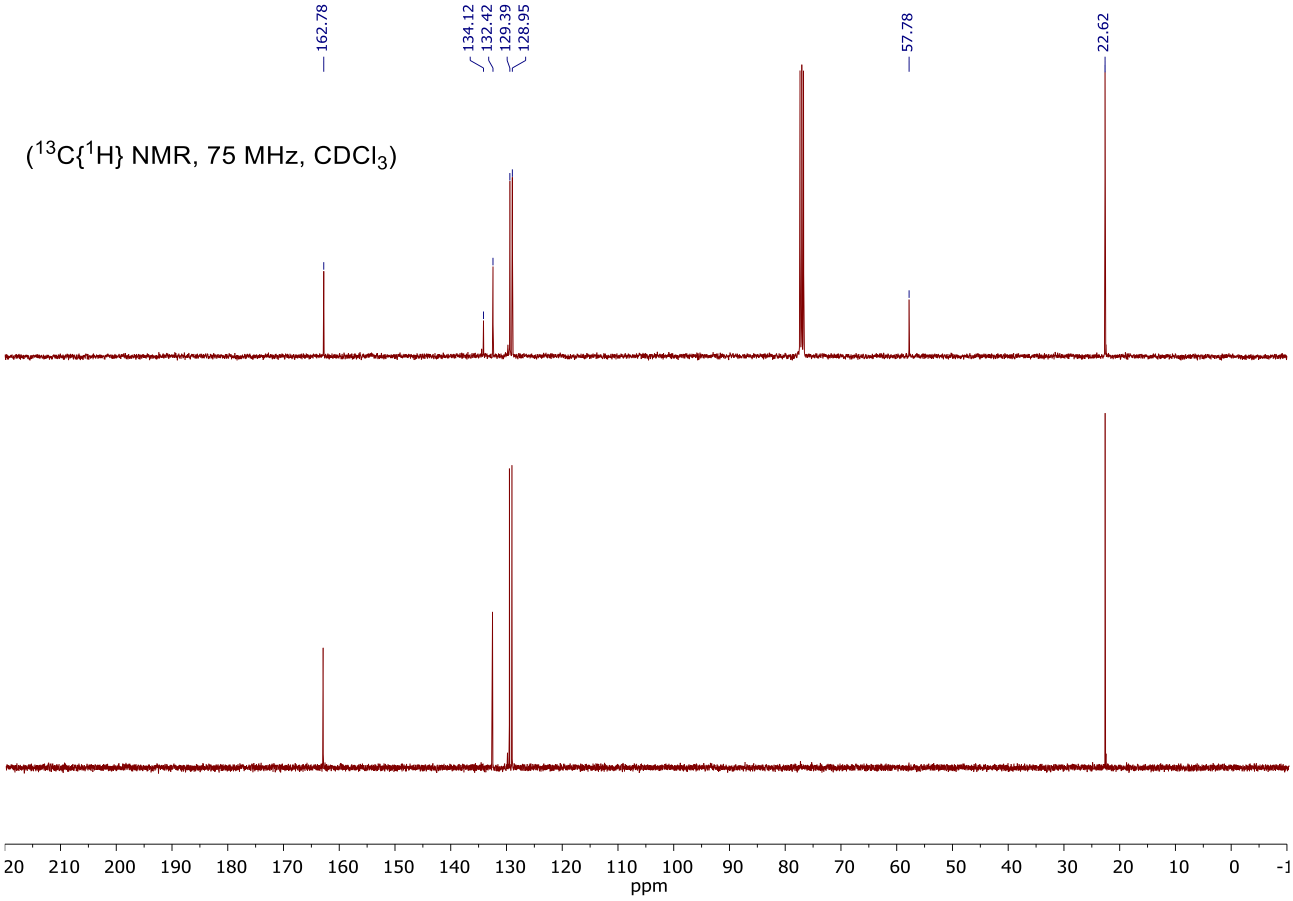

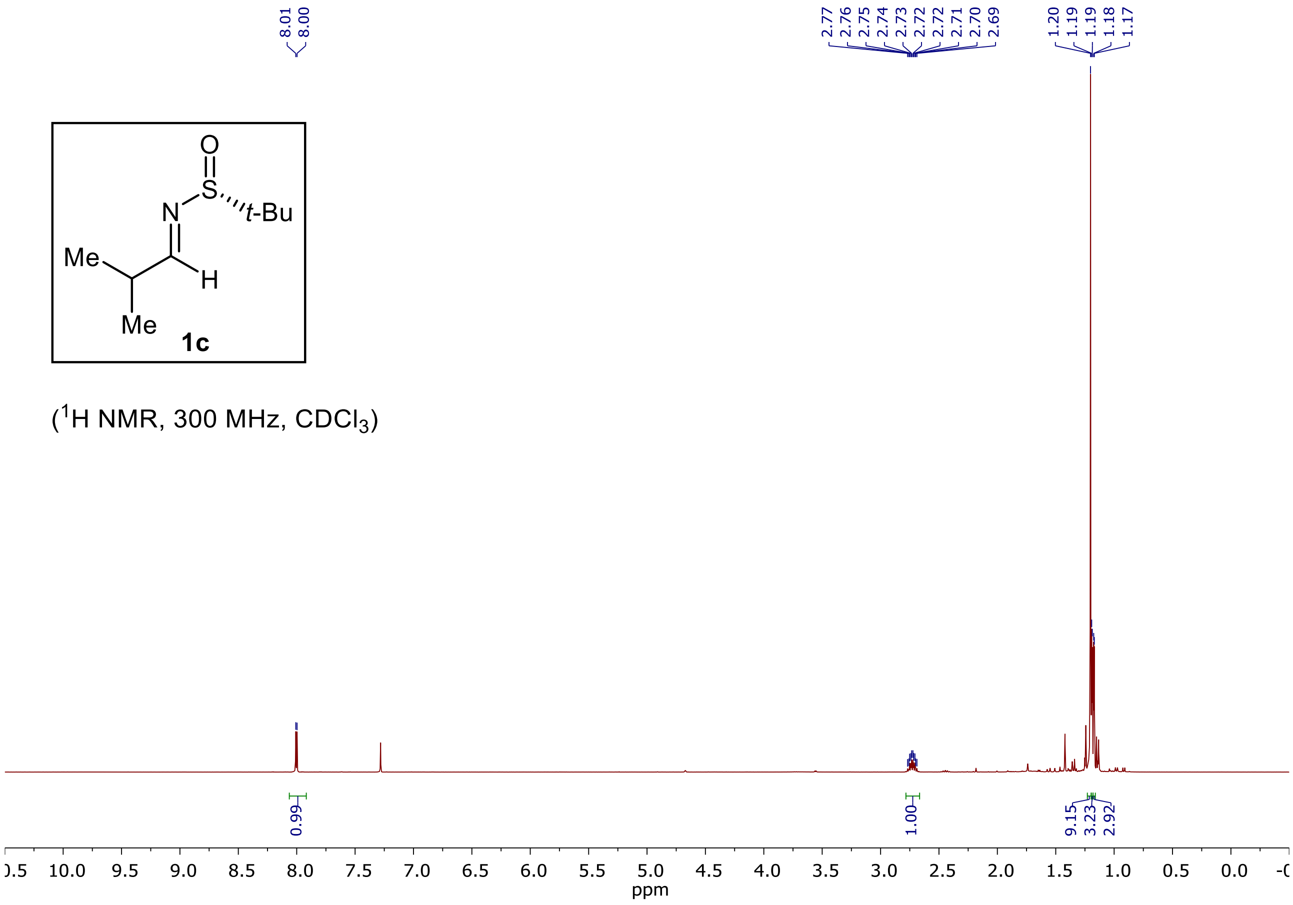
8.01
8.00
2.77
2.76
2.75
2.74
2.73
2.72
2.72
2.71
2.70
2.69
1.20
1.19
1.19
1.18
1.17
O
S
N
t-Bu
Me
H
Me
1c
(^{1}H NMR, 300 MHz, CDCl$_3$)
0.99
1.00
9.15
3.23
2.92
10.0
9.5
9.0
8.5
8.0
7.5
7.0
6.5
6.0
5.5
5.0
4.5
4.0
3.5
3.0
2.5
2.0
1.5
1.0
0.5
0.0
ppm

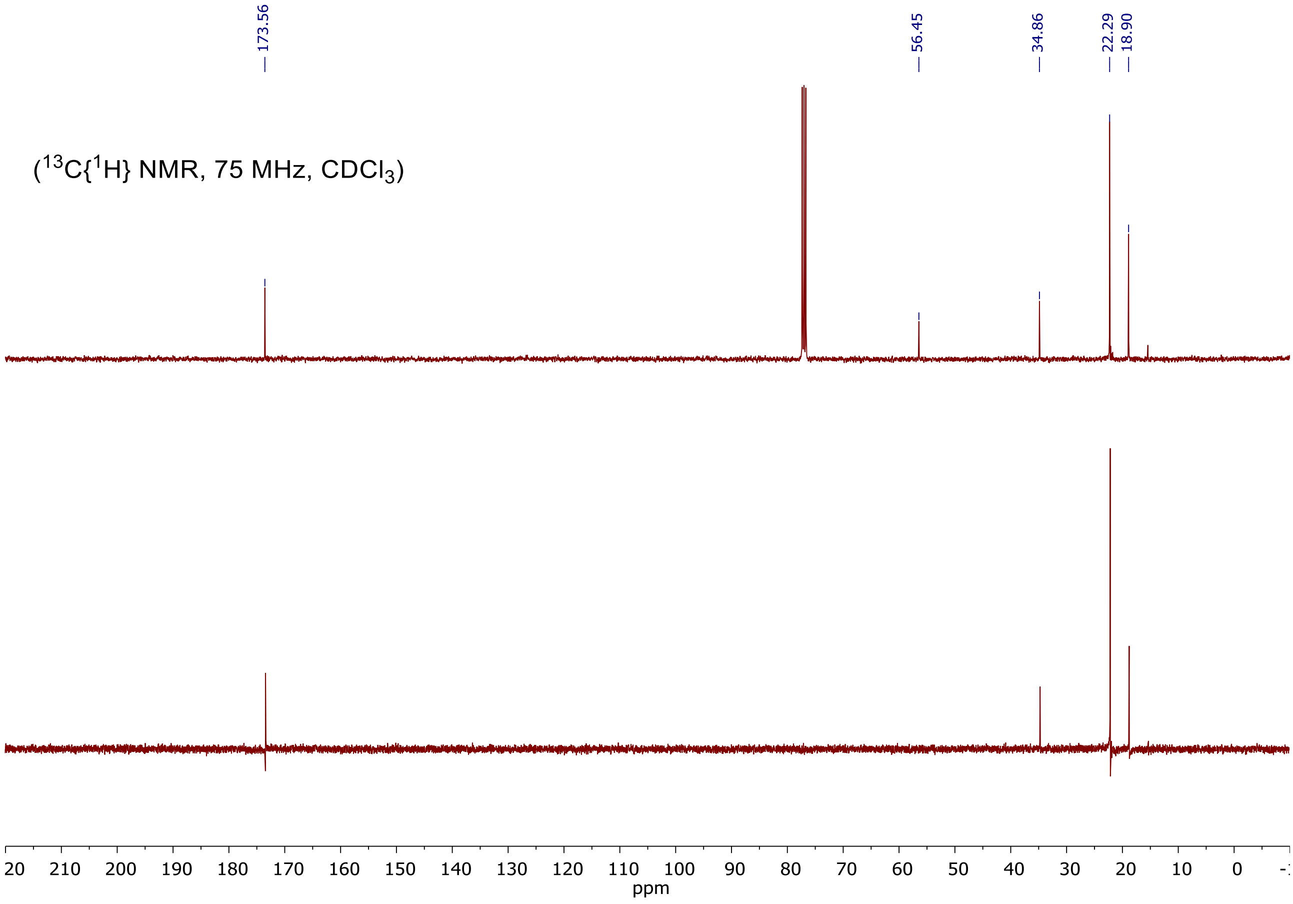
173.56
56.45
34.86
22.29
18.90
(^{13}C{^{1}H} NMR, 75 MHz, CDCl$_3$)
210
200
190
180
170
160
150
140
130
120
110
100
90
80
70
60
50
40
30
20
10
0
ppm

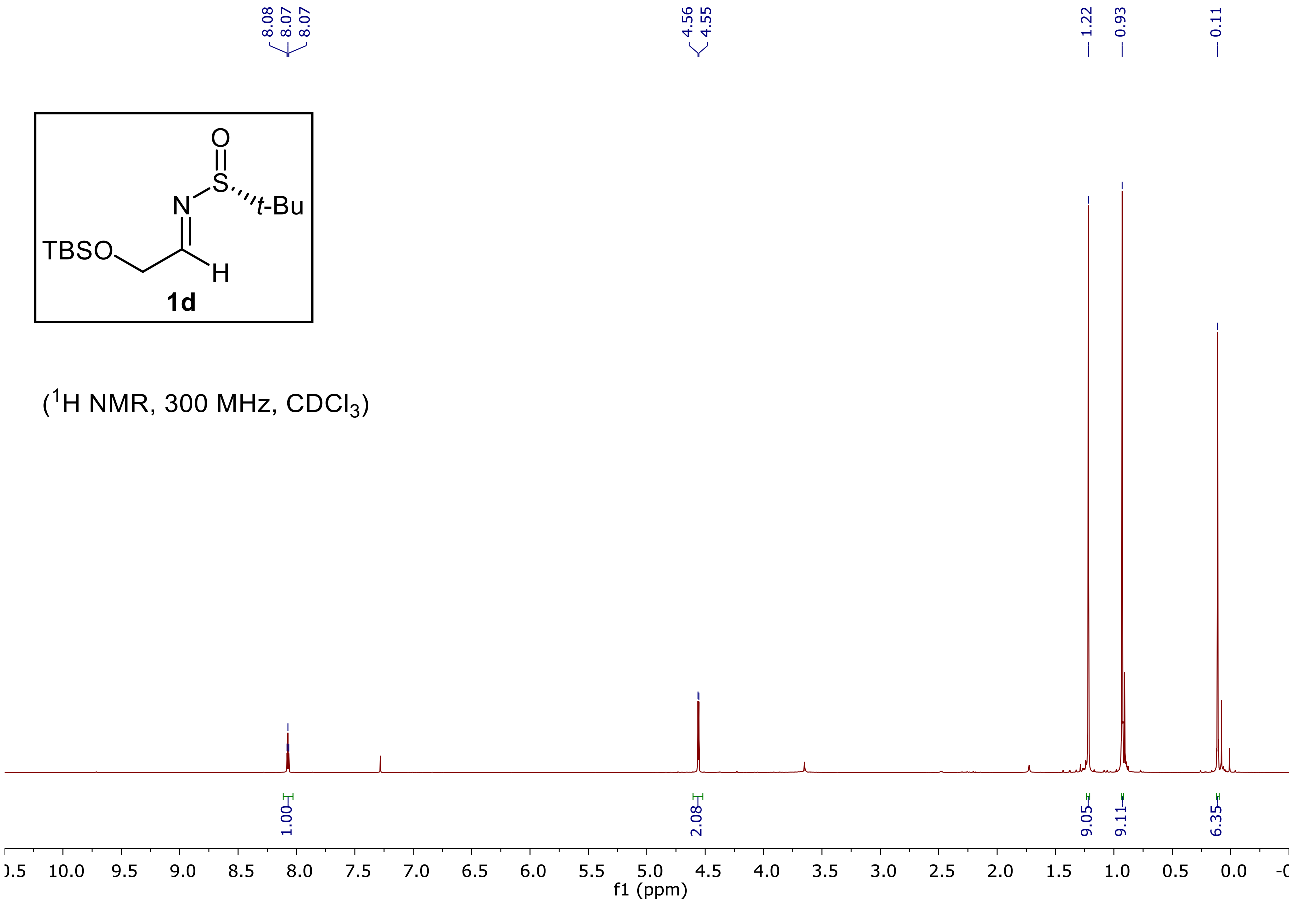
8.08
8.07
8.07
4.56
4.55
1.22
0.93
0.11
TBSO
N
S
O
t-Bu
H
1d
(¹H NMR, 300 MHz, CDCl₃)
1.00
2.08
9.05
9.11
6.35
f1 (ppm)

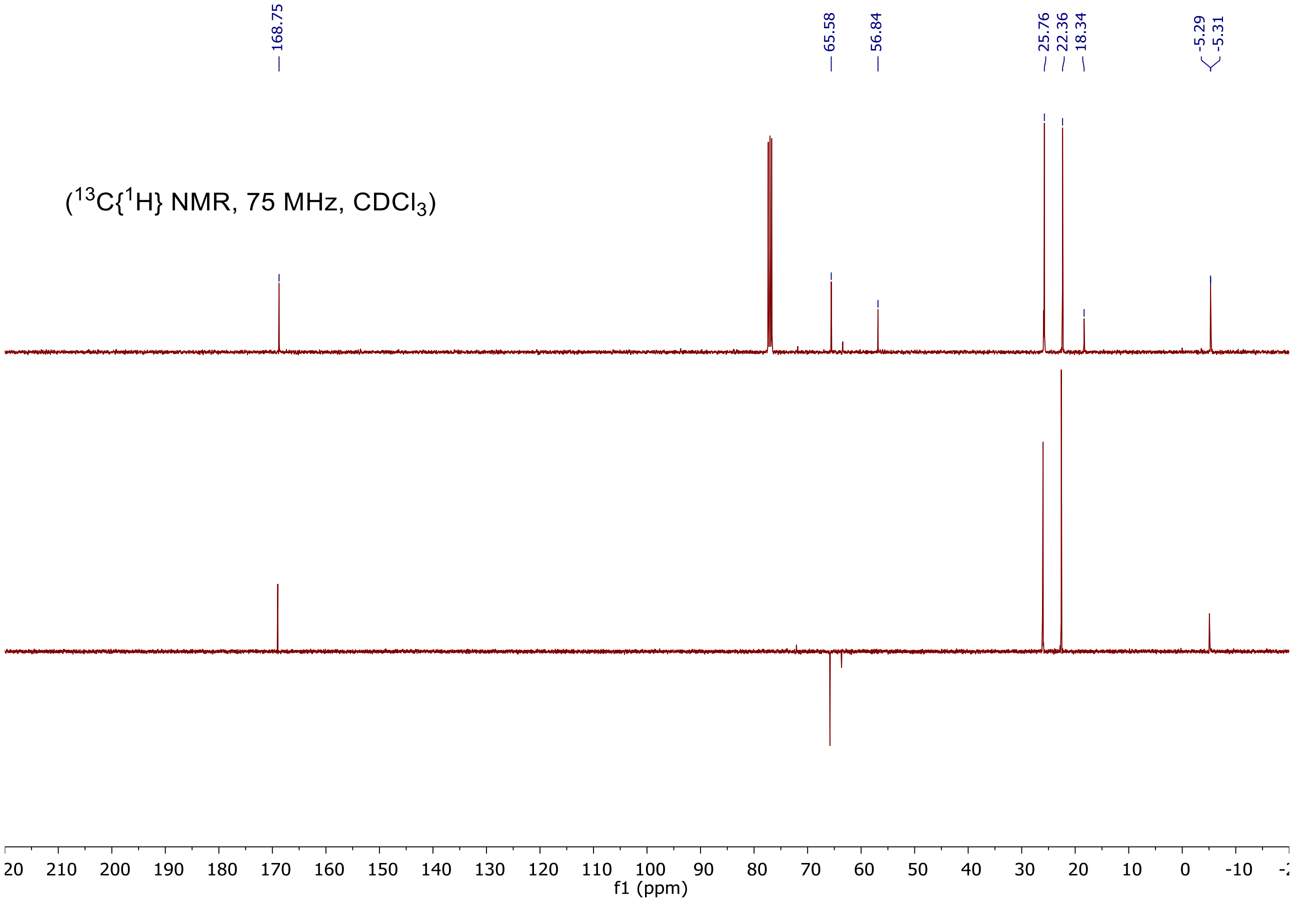
168.75
65.58
56.84
25.76
22.36
18.34
-5.29
-5.31
(¹³C{¹H} NMR, 75 MHz, CDCl₃)
f1 (ppm)

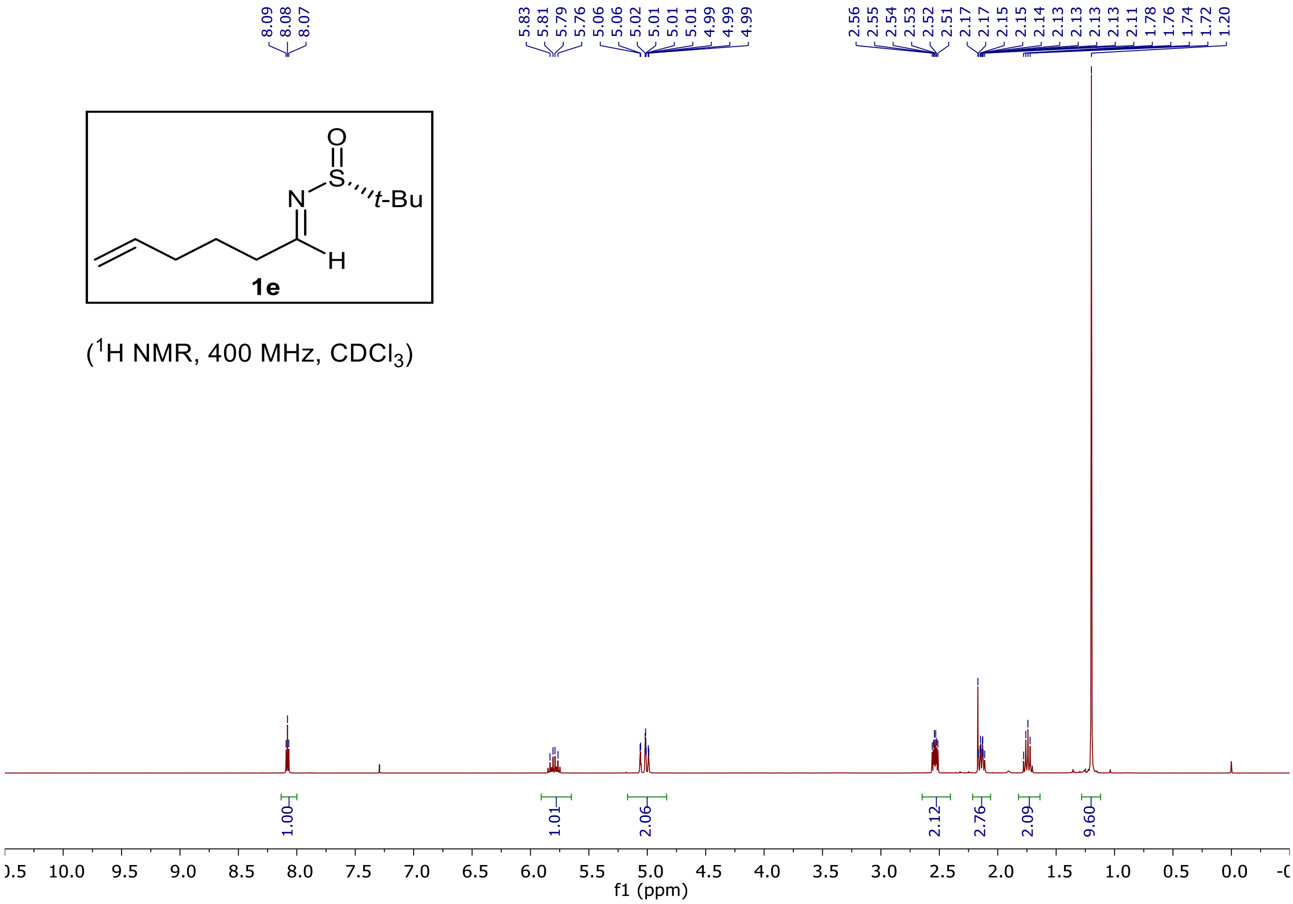

(^{1}H NMR, 400 MHz, $CDCl_3$)

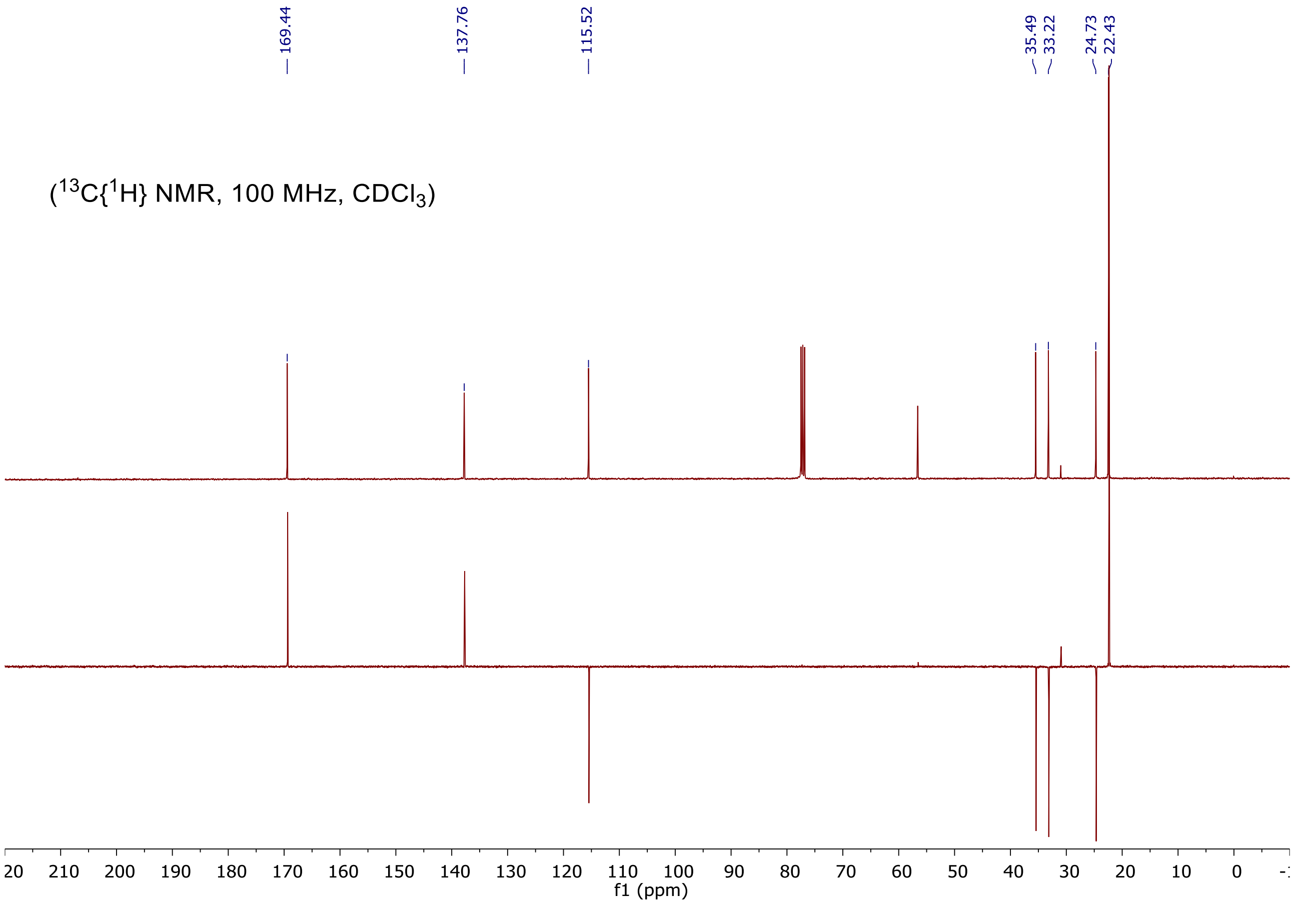

($^{13}C\{^1H\}$ NMR, 100 MHz, $CDCl_3$)

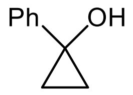

2b

(^{1}H NMR, 400 MHz, $CDCl_3$)

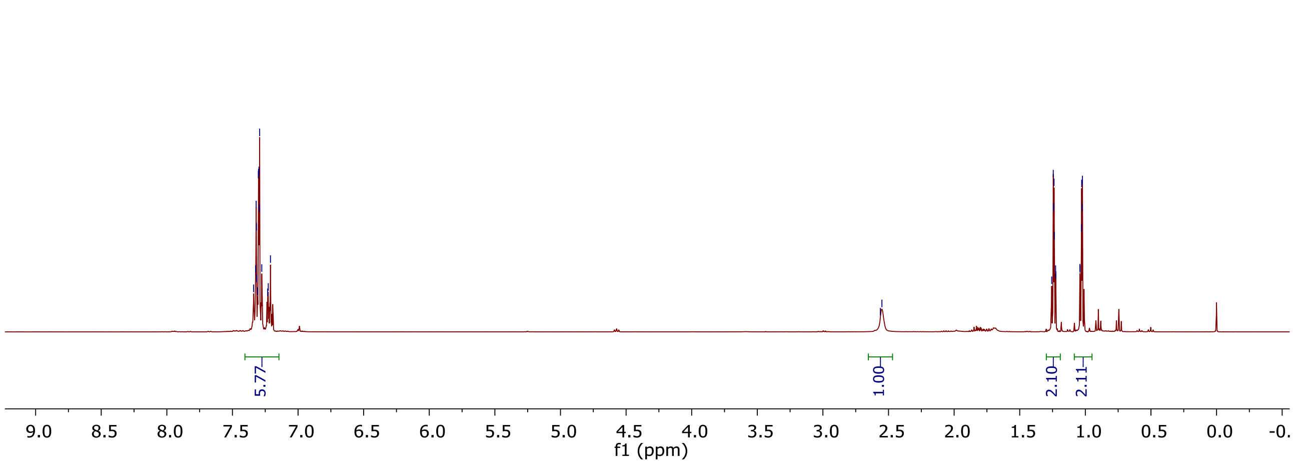

(^{13}C{^{1}H} NMR, 100 MHz, $CDCl_3$)

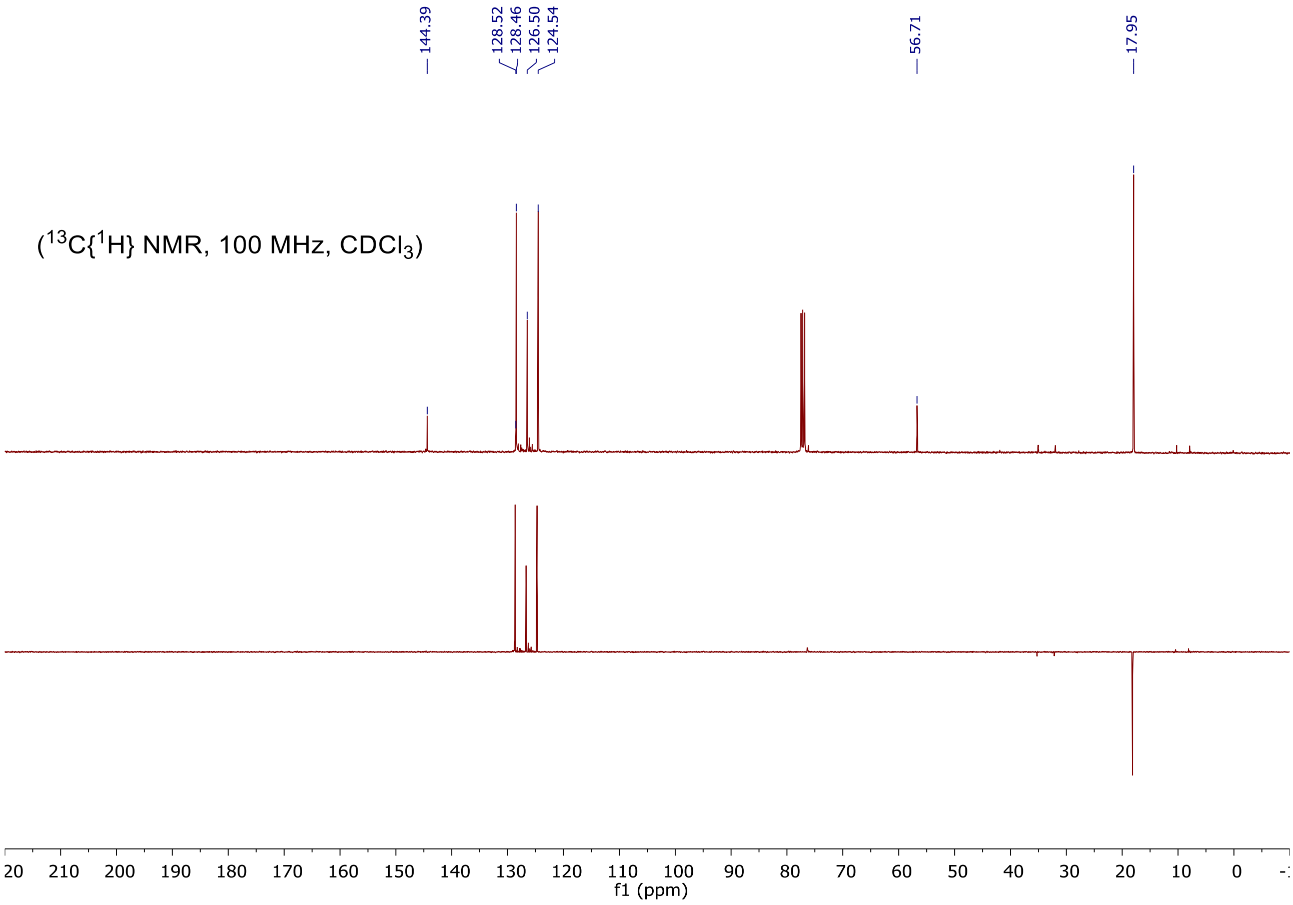

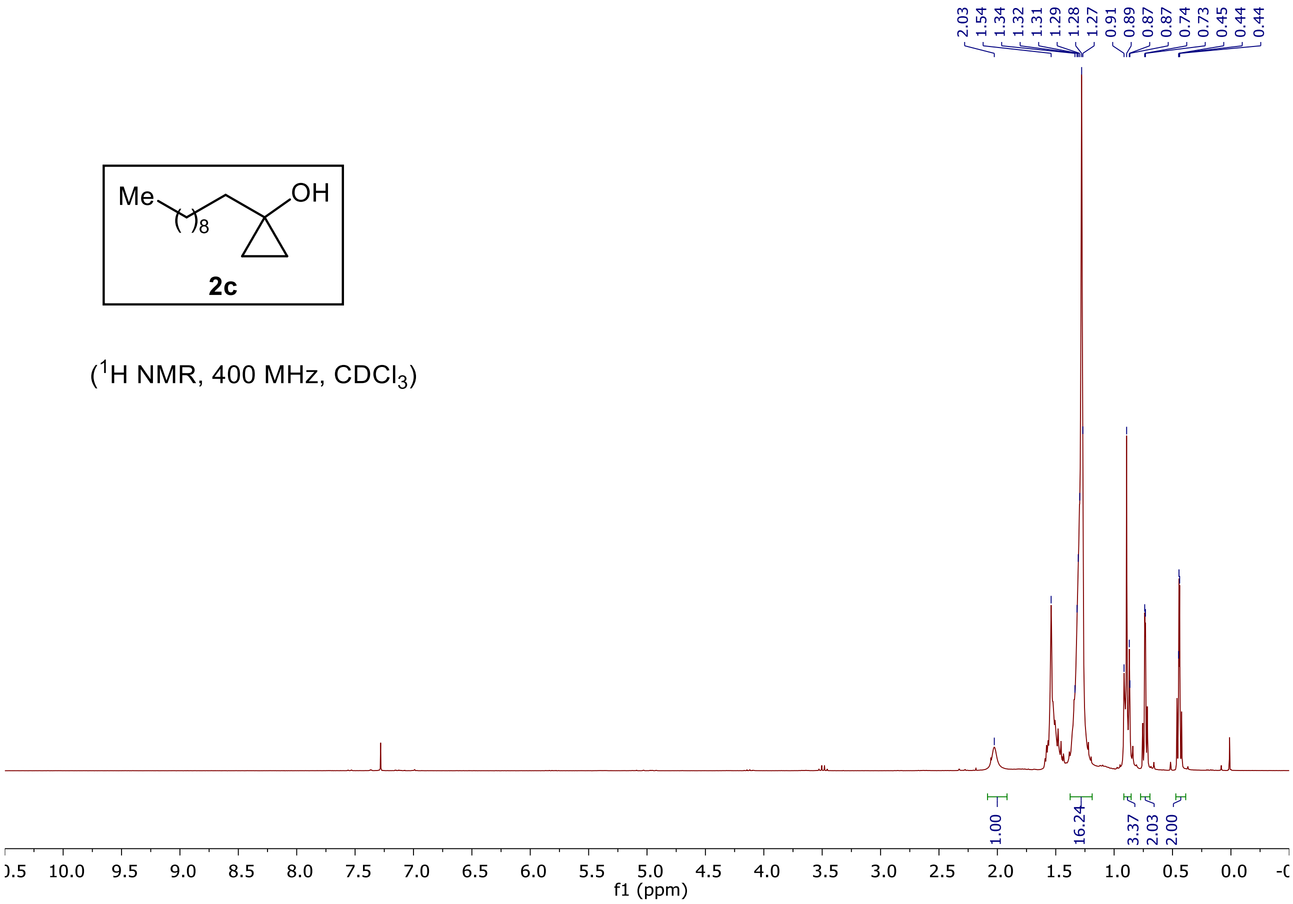

(^{13}C{^{1}H} NMR, 100 MHz, $CDCl_3$)

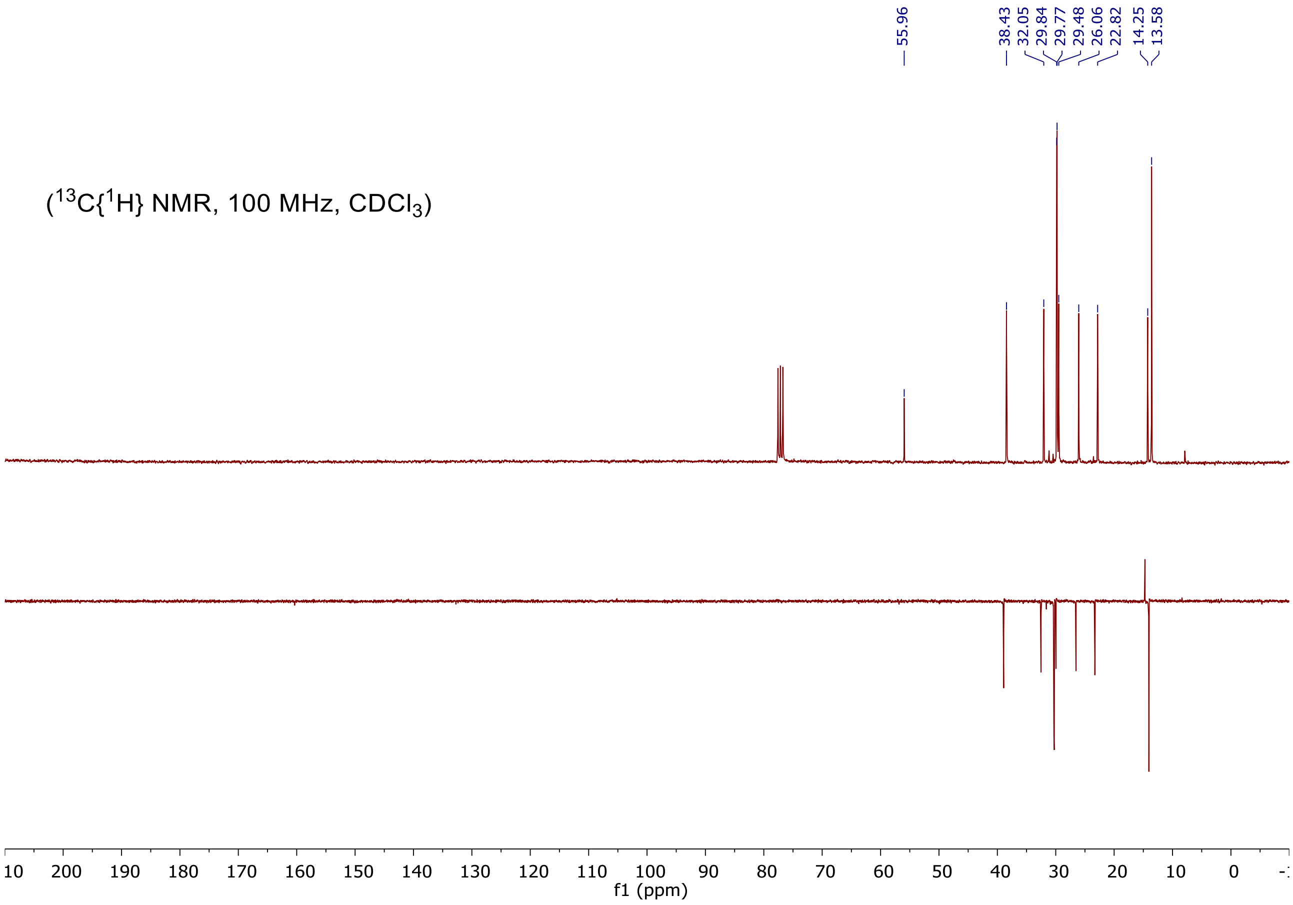

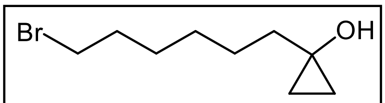

2d

(^{1}H NMR, 400 MHz, $CDCl_3$)

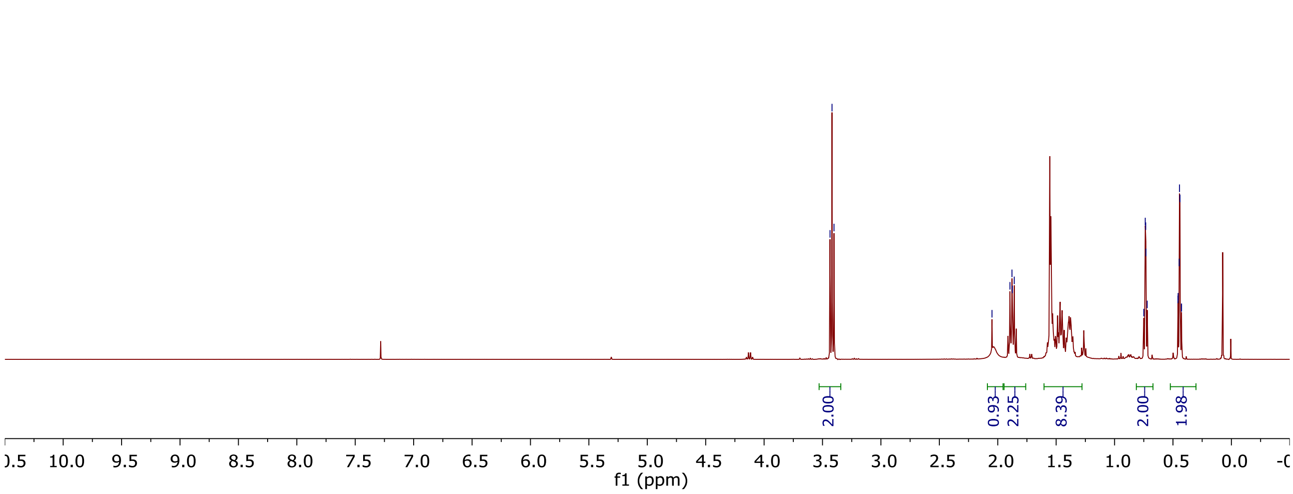

(^{13}C{^{1}H} NMR, 100 MHz, $CDCl_3$)

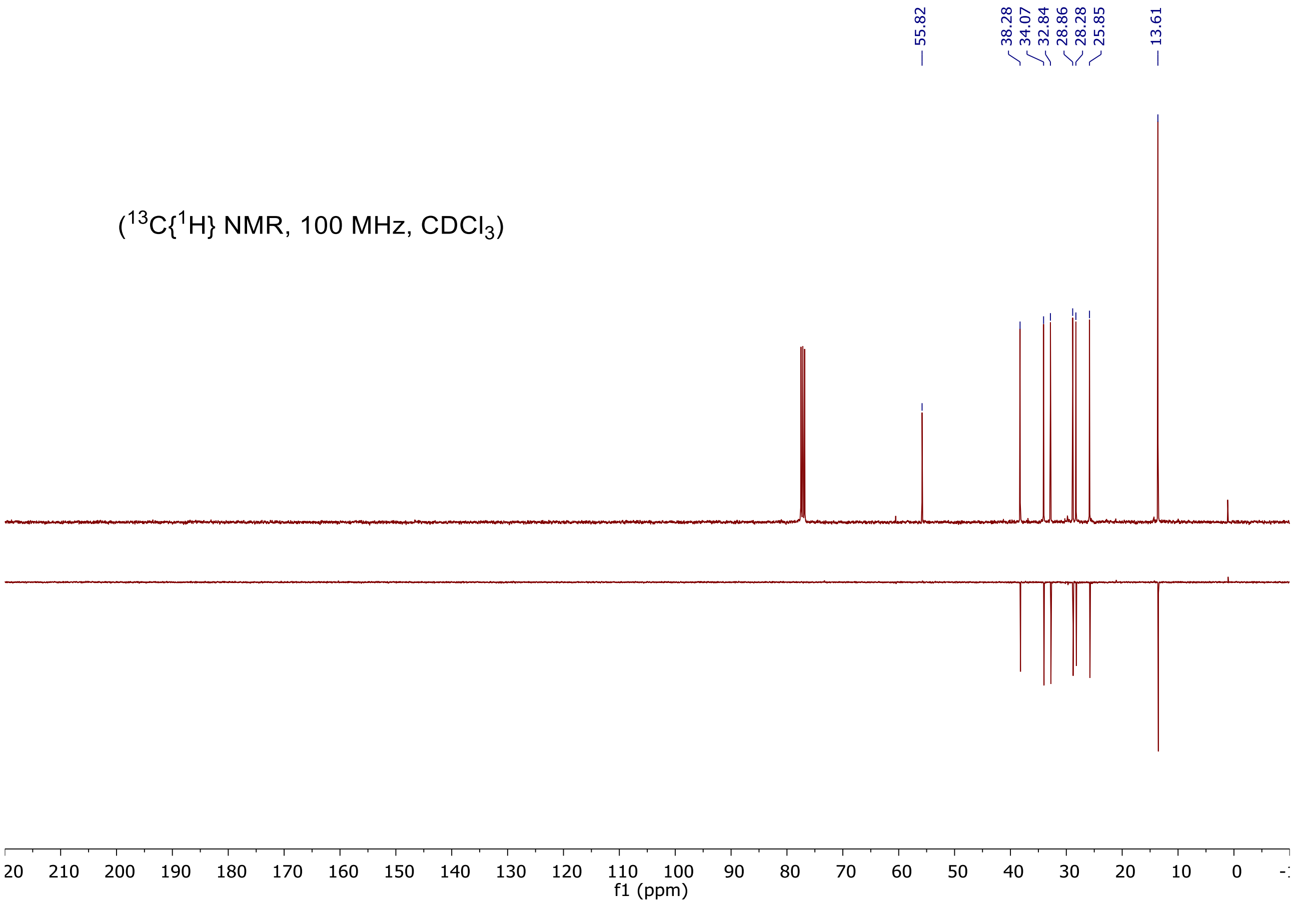

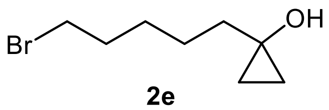

2e

(^{1}H NMR, 400 MHz, $CDCl_3$)

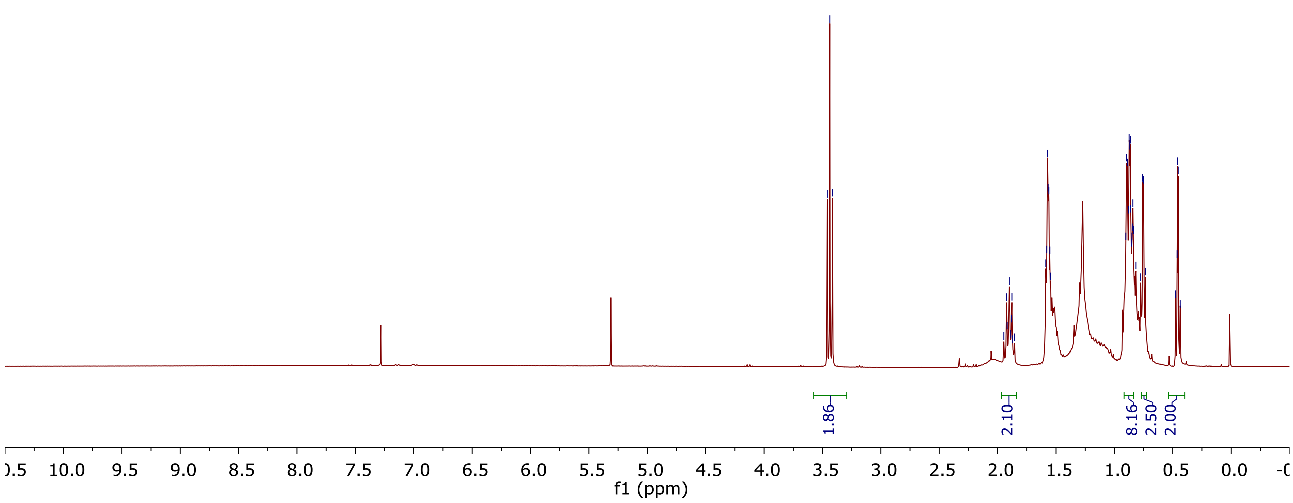

(^{13}C{^{1}H} NMR, 100 MHz, $CDCl_3$)

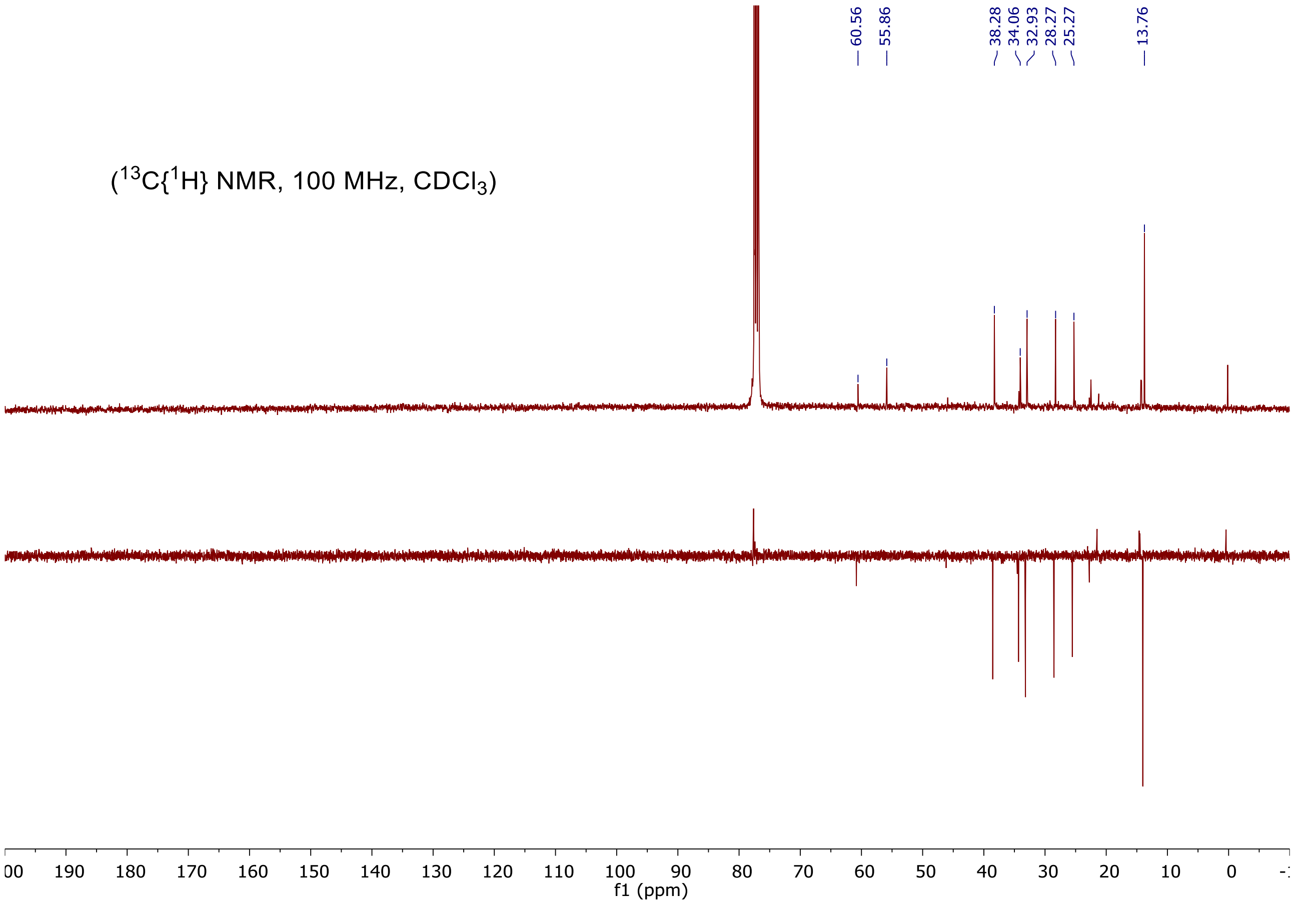

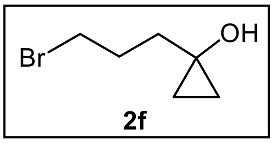

(¹H NMR, 400 MHz, $CDCl_3$)

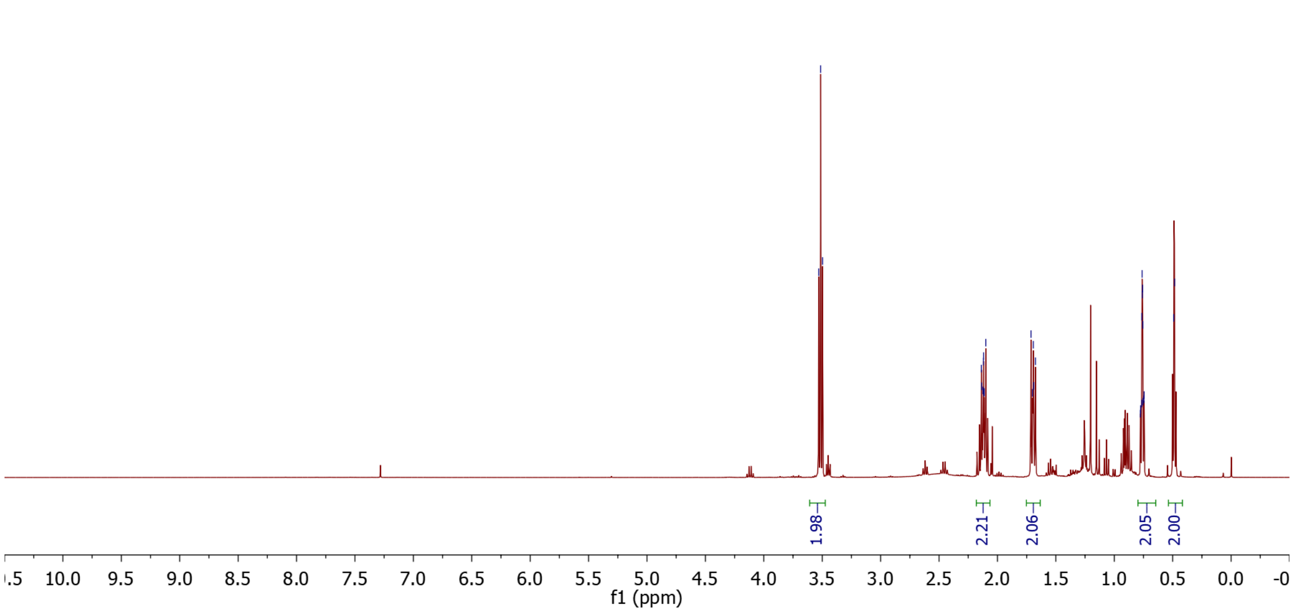

(¹³C{¹H} NMR, 100 MHz, $CDCl_3$)

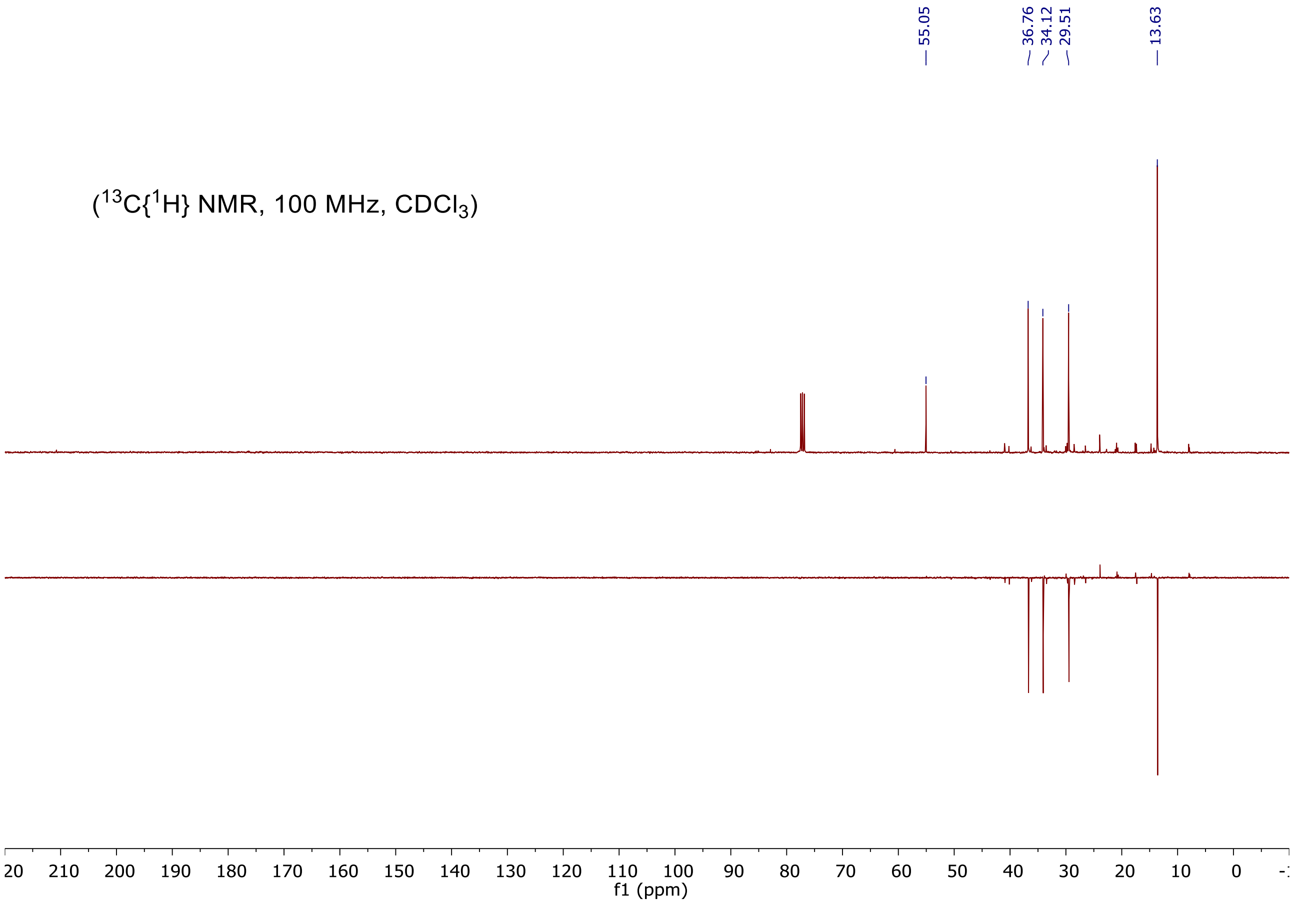

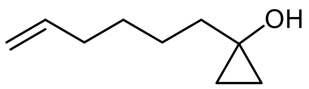

2g

(¹H NMR, 400 MHz, $CDCl_3$)

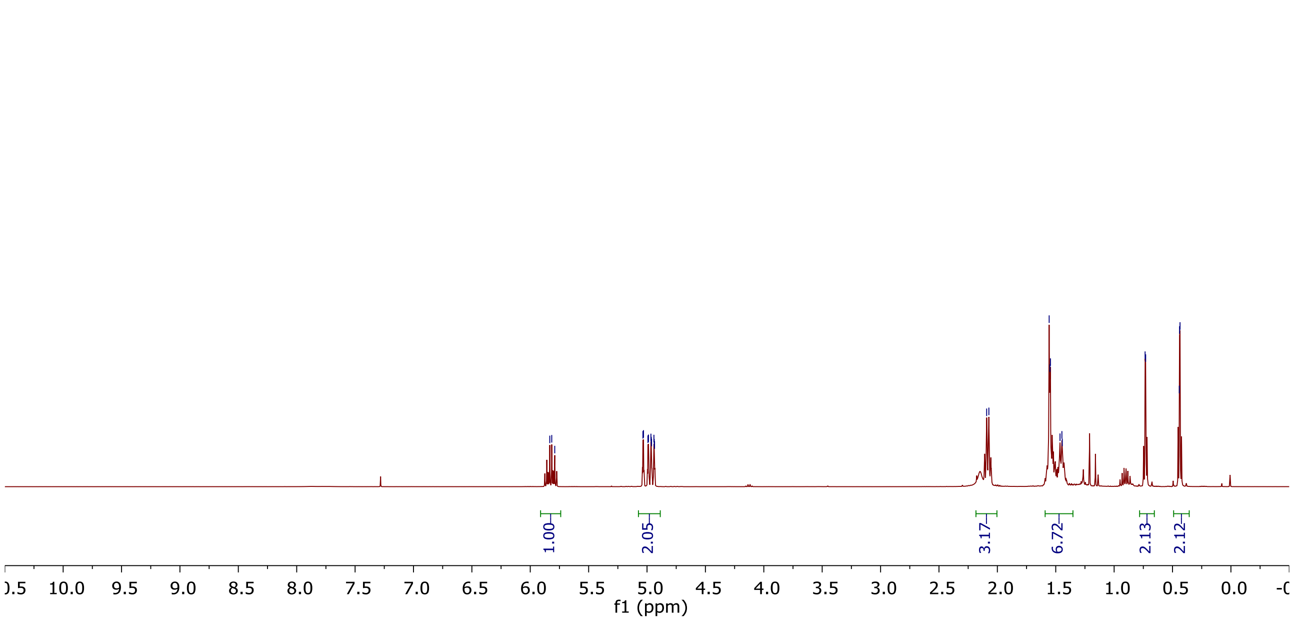

(¹³C{¹H} NMR, 100 MHz, $CDCl_3$)

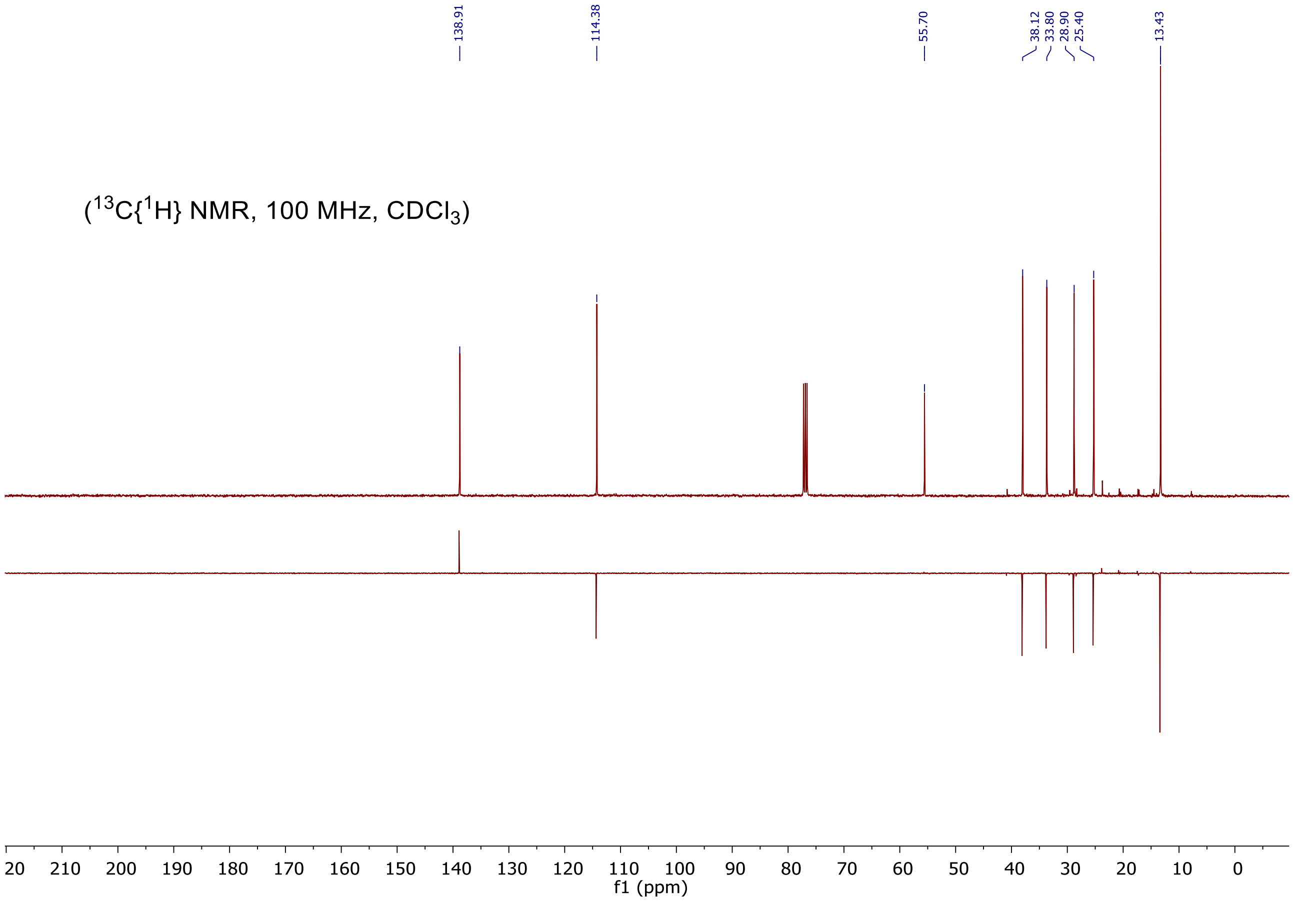

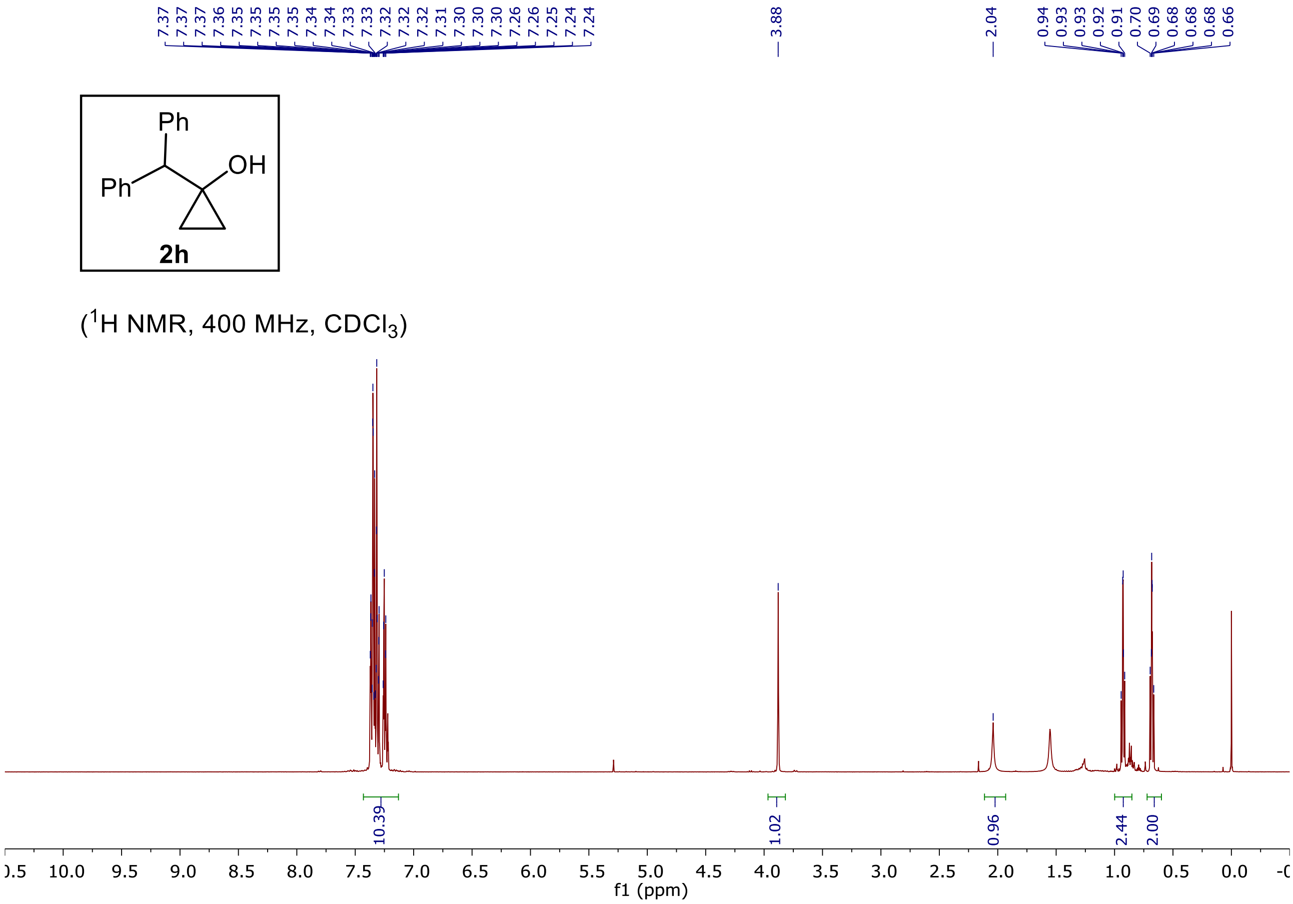

(^{1}H NMR, 400 MHz, $CDCl_3$)

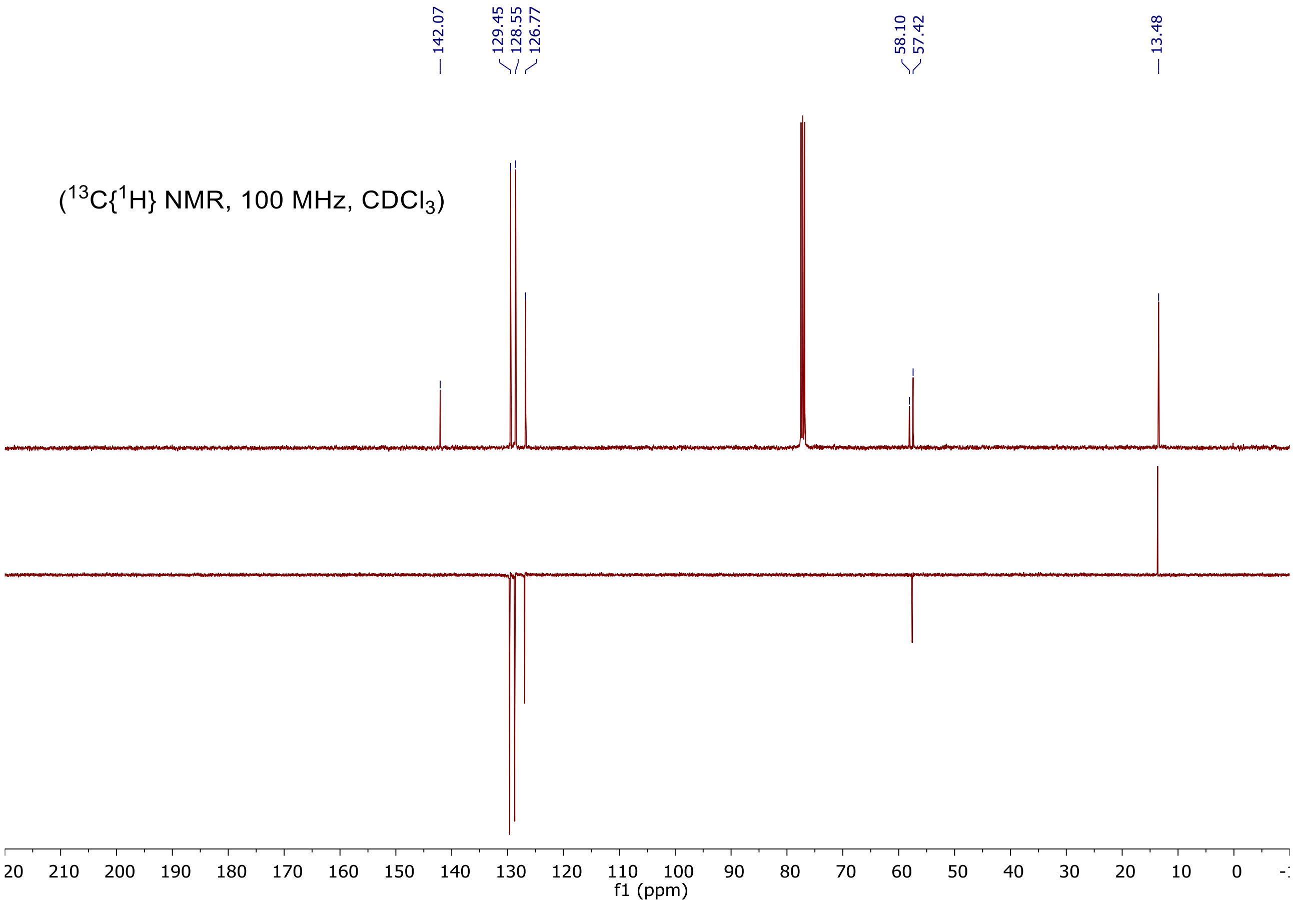

(^{13}C{^{1}H} NMR, 100 MHz, $CDCl_3$)

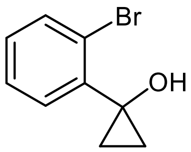

2i

(^{1}H NMR, 400 MHz, $CDCl_3$)

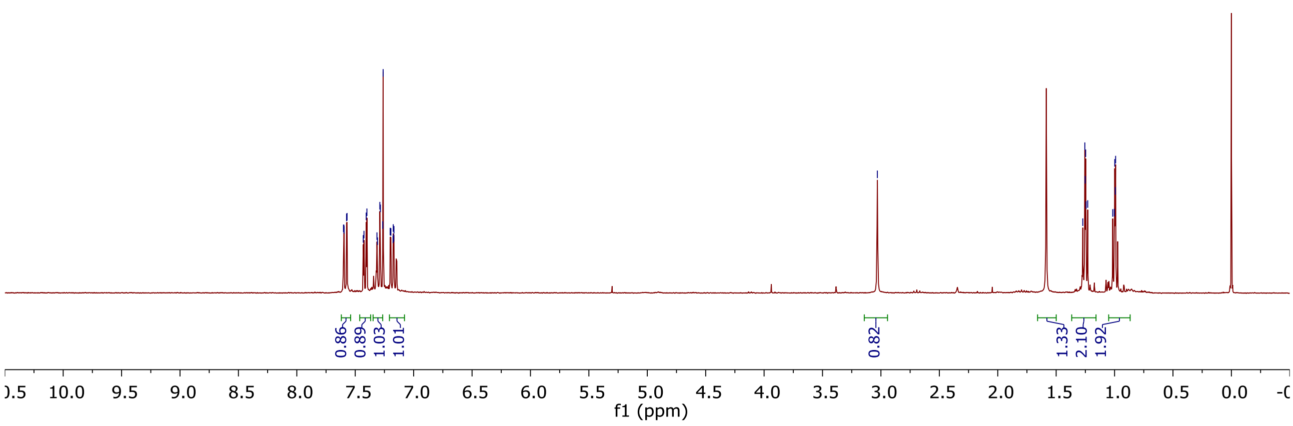

(^{13}C{^{1}H} NMR, 100 MHz, $CDCl_3$)

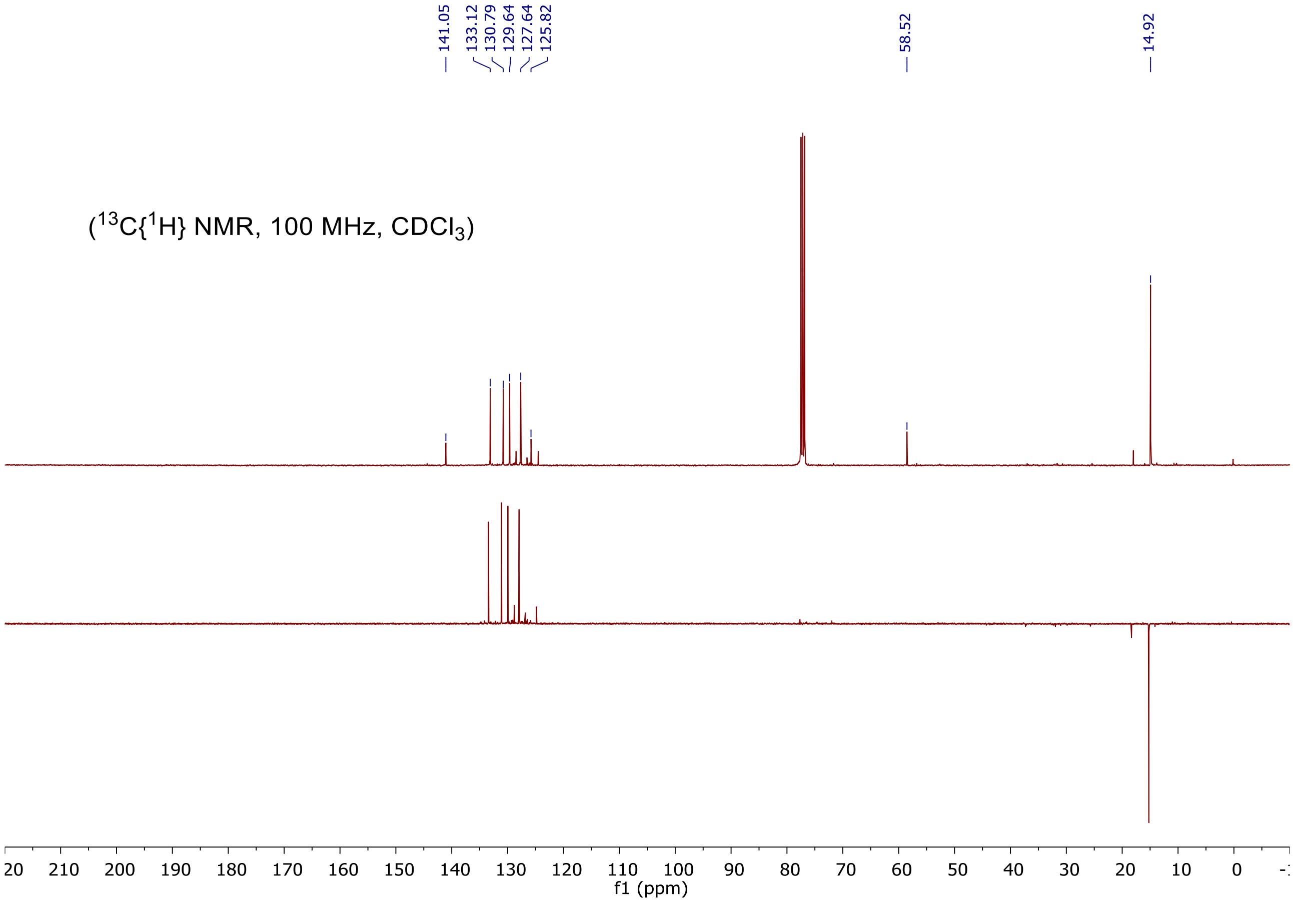

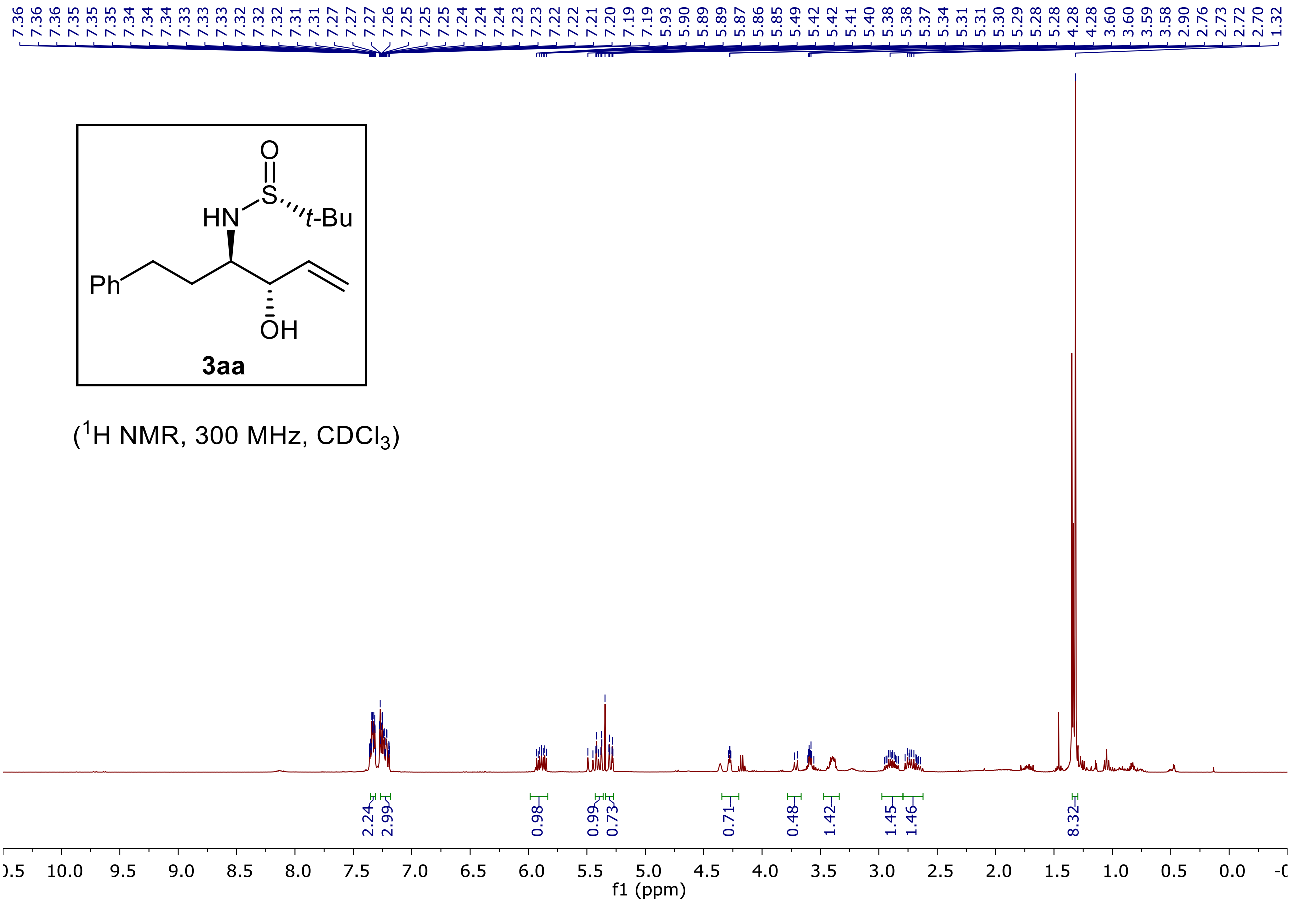

3aa

(^{1}H NMR, 300 MHz, $CDCl_3$)

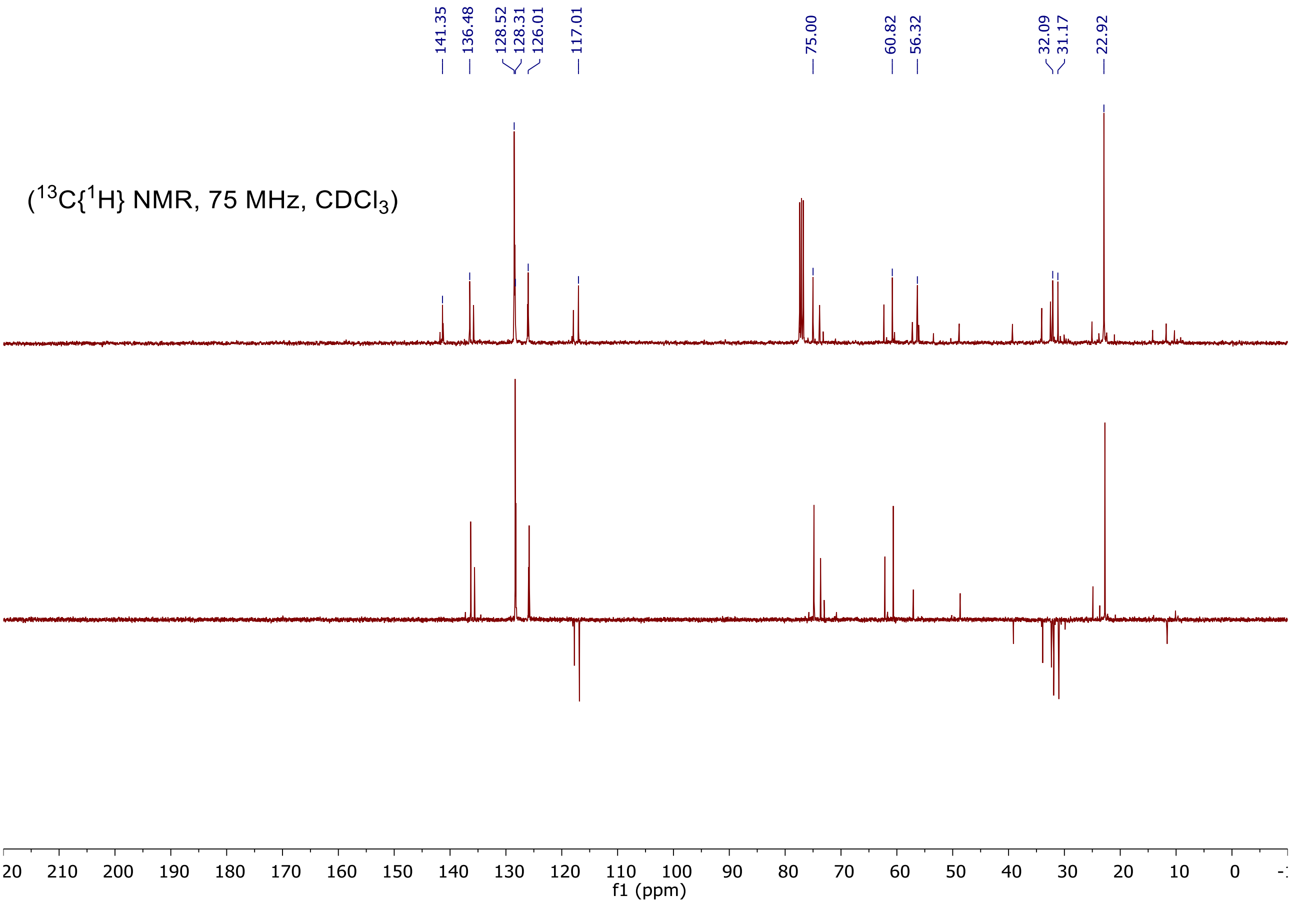

(^{13}C{^{1}H} NMR, 75 MHz, $CDCl_3$)

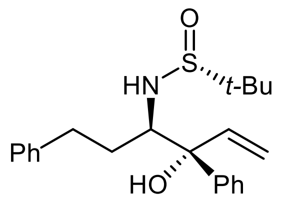

3ab

(^{1}H NMR, 400 MHz, $CDCl_3$)

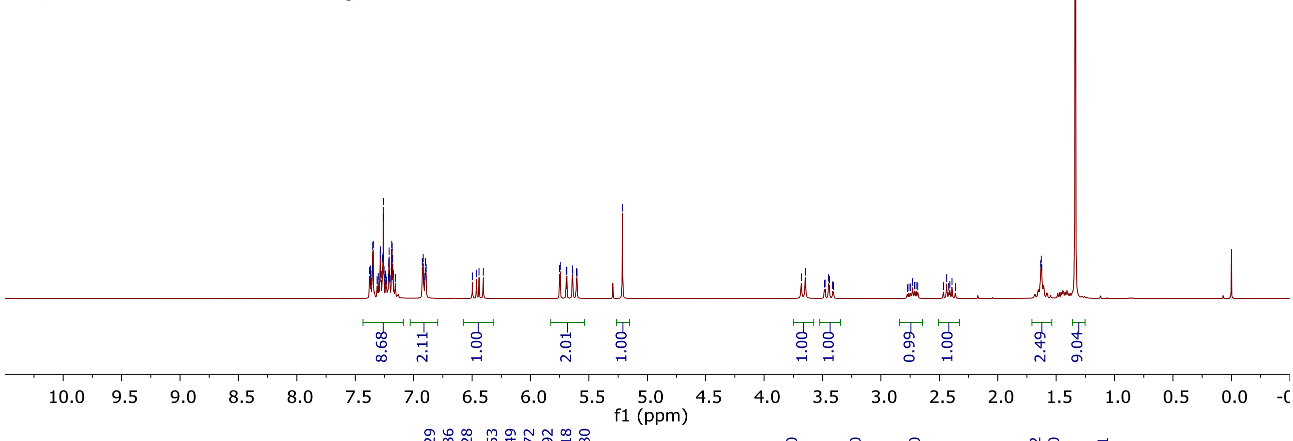

(${}^{13}C\{{}^{1}H\}$ NMR, 100 MHz, $CDCl_3$)

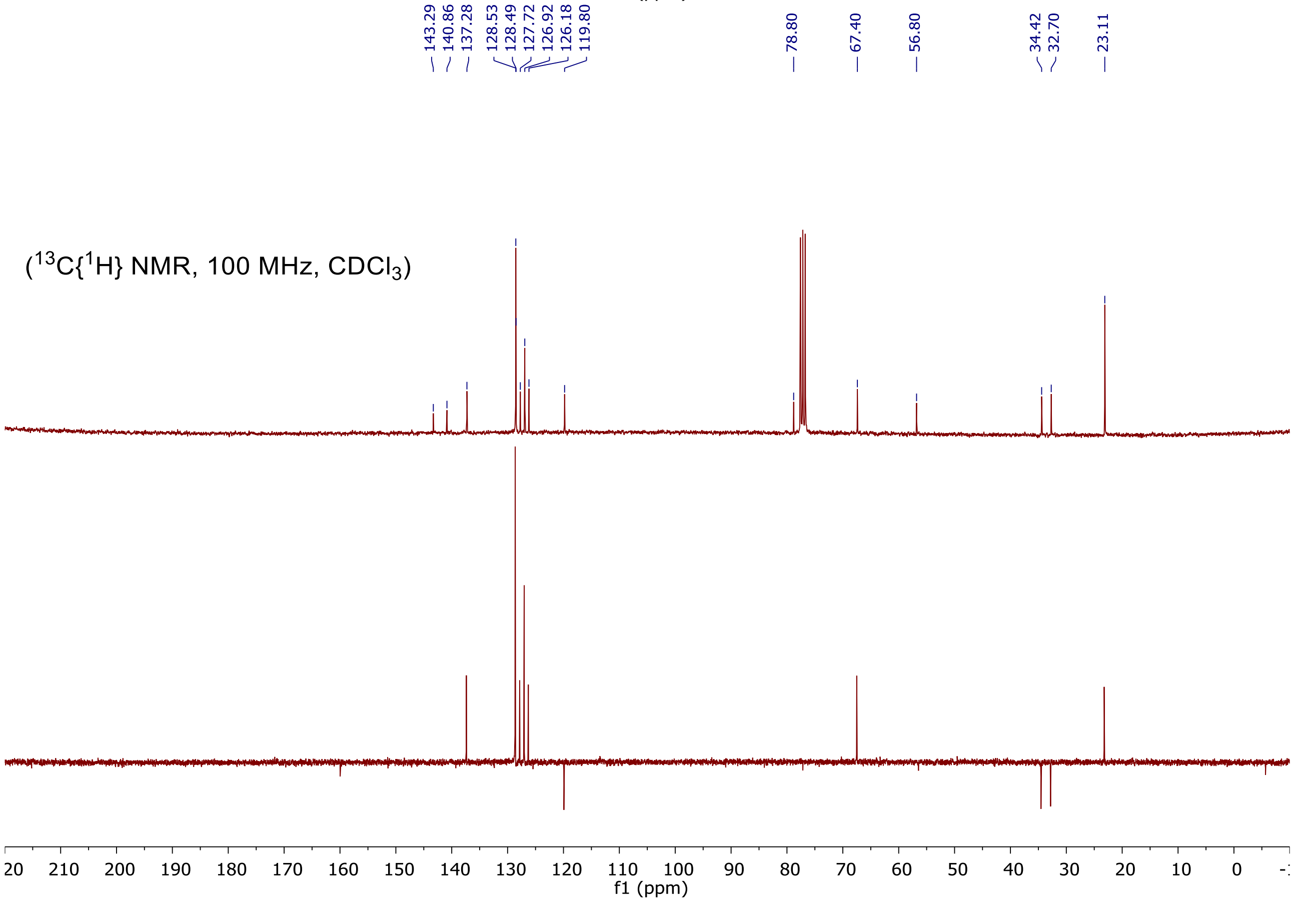

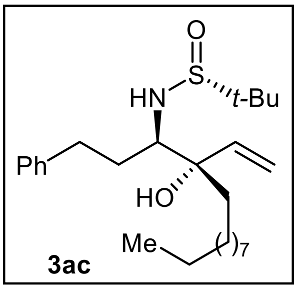

(¹H NMR, 400 MHz, $CDCl_3$)

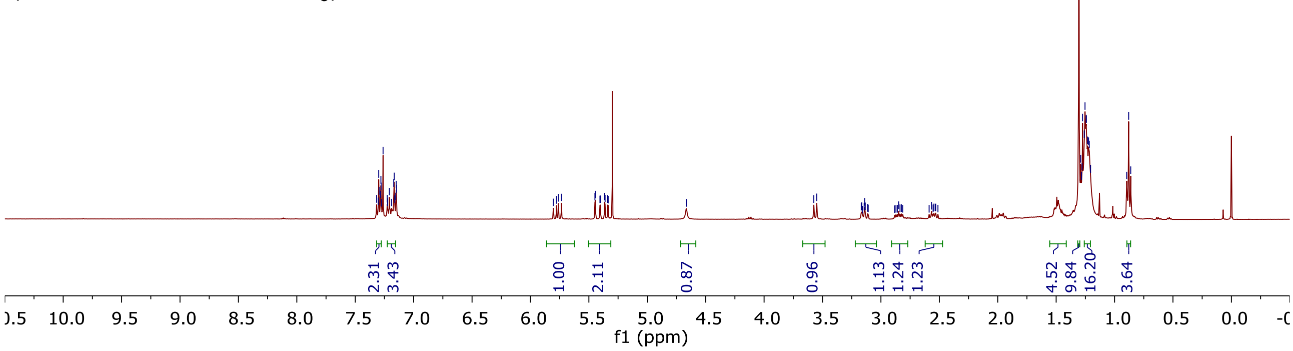

($^{13}C\{^1H\}$ NMR, 100 MHz, $CDCl_3$)

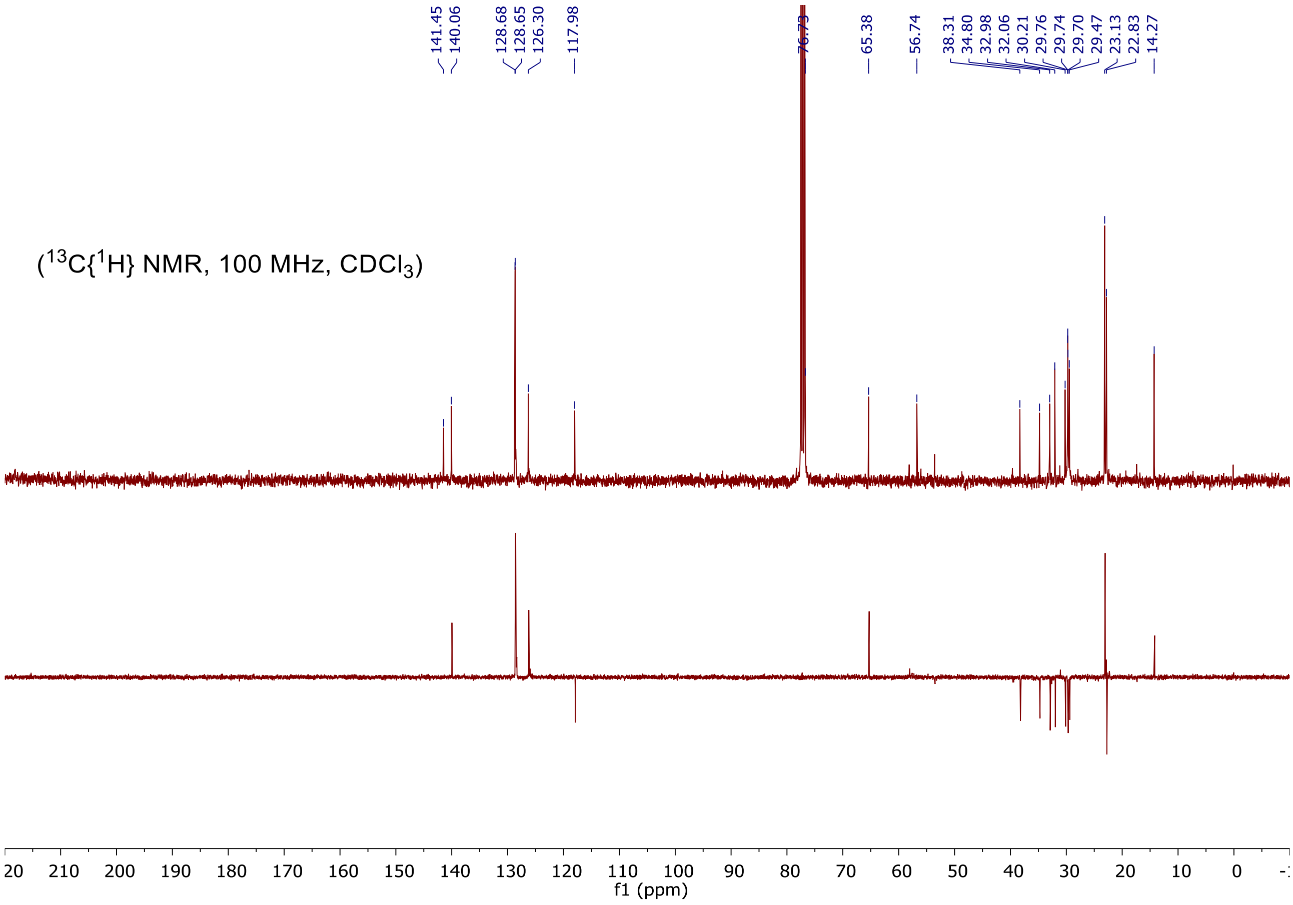

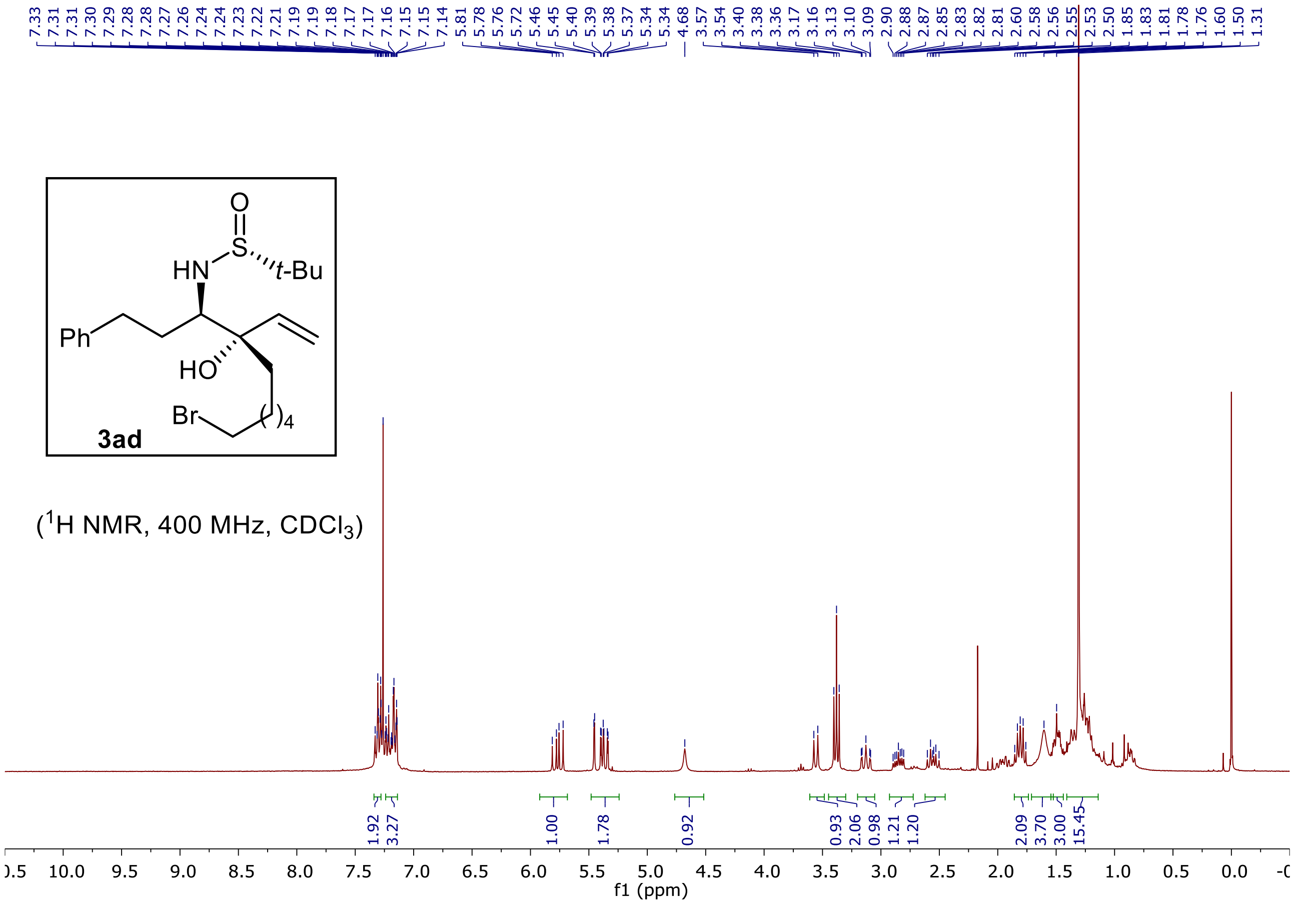
7.33 7.31 7.31 7.30 7.29 7.28 7.28 7.27 7.26 7.24 7.24 7.23 7.22 7.21 7.19 7.19 7.18 7.17 7.17 7.16 7.15 7.15 7.14 5.81 5.76 5.72 5.46 5.45 5.40 5.39 5.38 5.37 5.34 5.34 4.68 3.57 3.54 3.40 3.38 3.36 3.17 3.16 3.13 3.10 3.09 2.90 2.88 2.87 2.85 2.83 2.82 2.81 2.60 2.58 2.56 2.55 2.53 2.50 1.85 1.83 1.81 1.78 1.76 1.60 1.50 1.31
HN
S
O
t-Bu
Ph
HO
Br
4
3ad
(1H NMR, 400 MHz, CDCl3)
1.92 3.27 1.00 1.78 0.92 0.93 2.06 0.98 1.21 1.20 2.09 3.70 3.00 15.45
f1 (ppm)

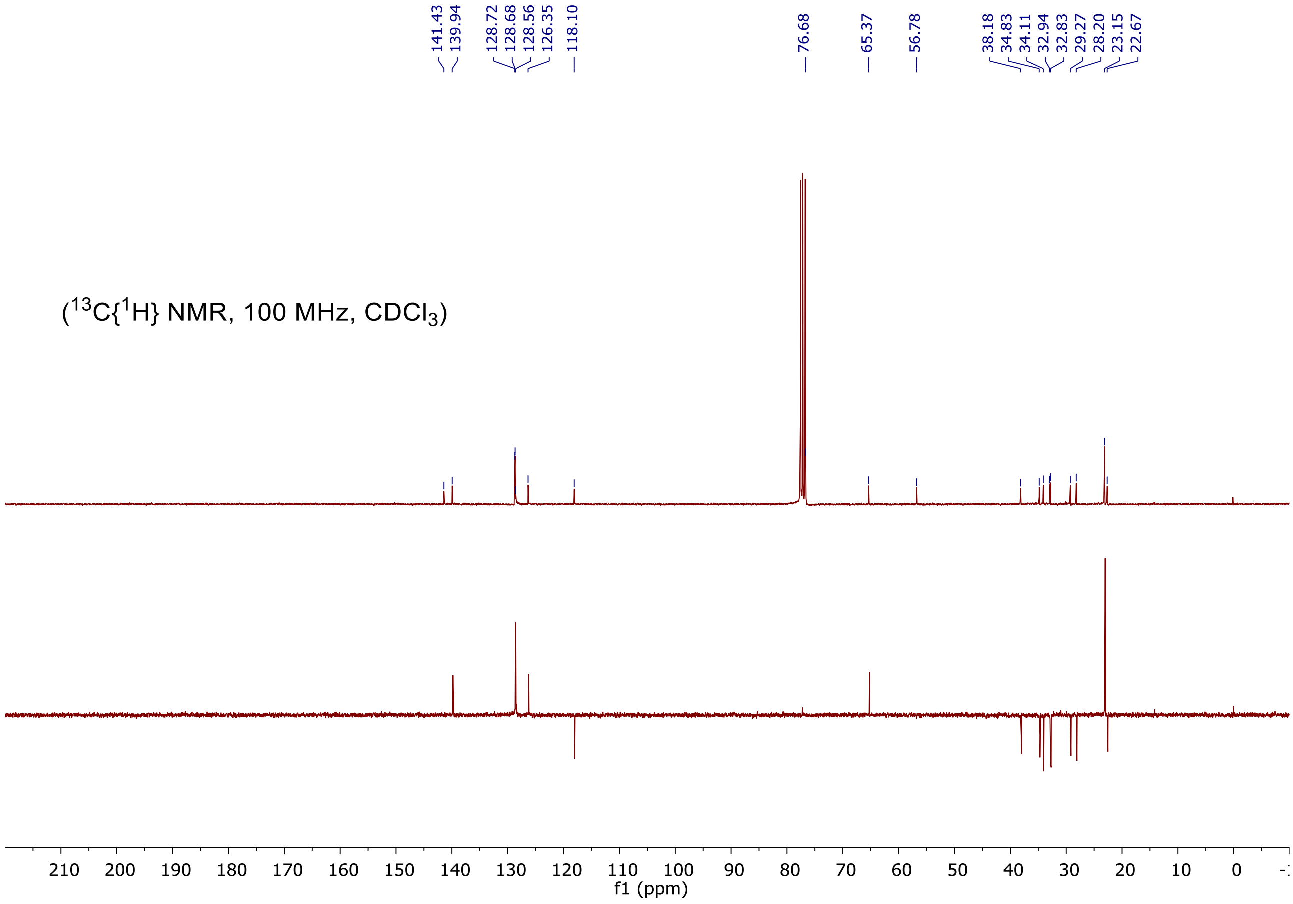
141.43 139.94 128.72 128.68 128.56 126.35 118.10 76.68 65.37 56.78 38.18 34.83 34.11 32.94 32.83 29.27 28.20 23.15 22.67
(13C{1H} NMR, 100 MHz, CDCl3)
f1 (ppm)

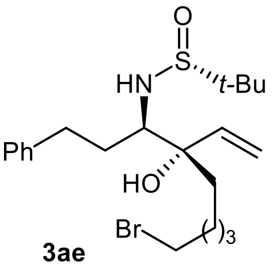

3ae

(¹H NMR, 400 MHz, CDCl₃)

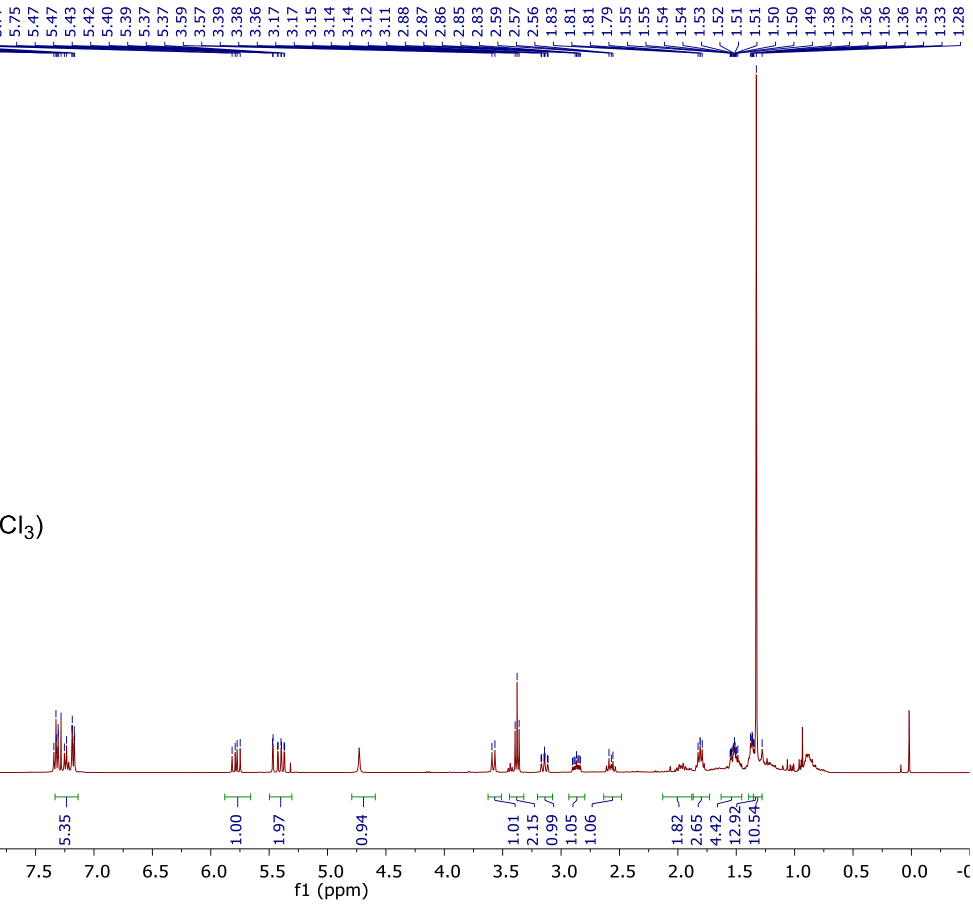

(¹³C{¹H} NMR, 100 MHz, CDCl₃)

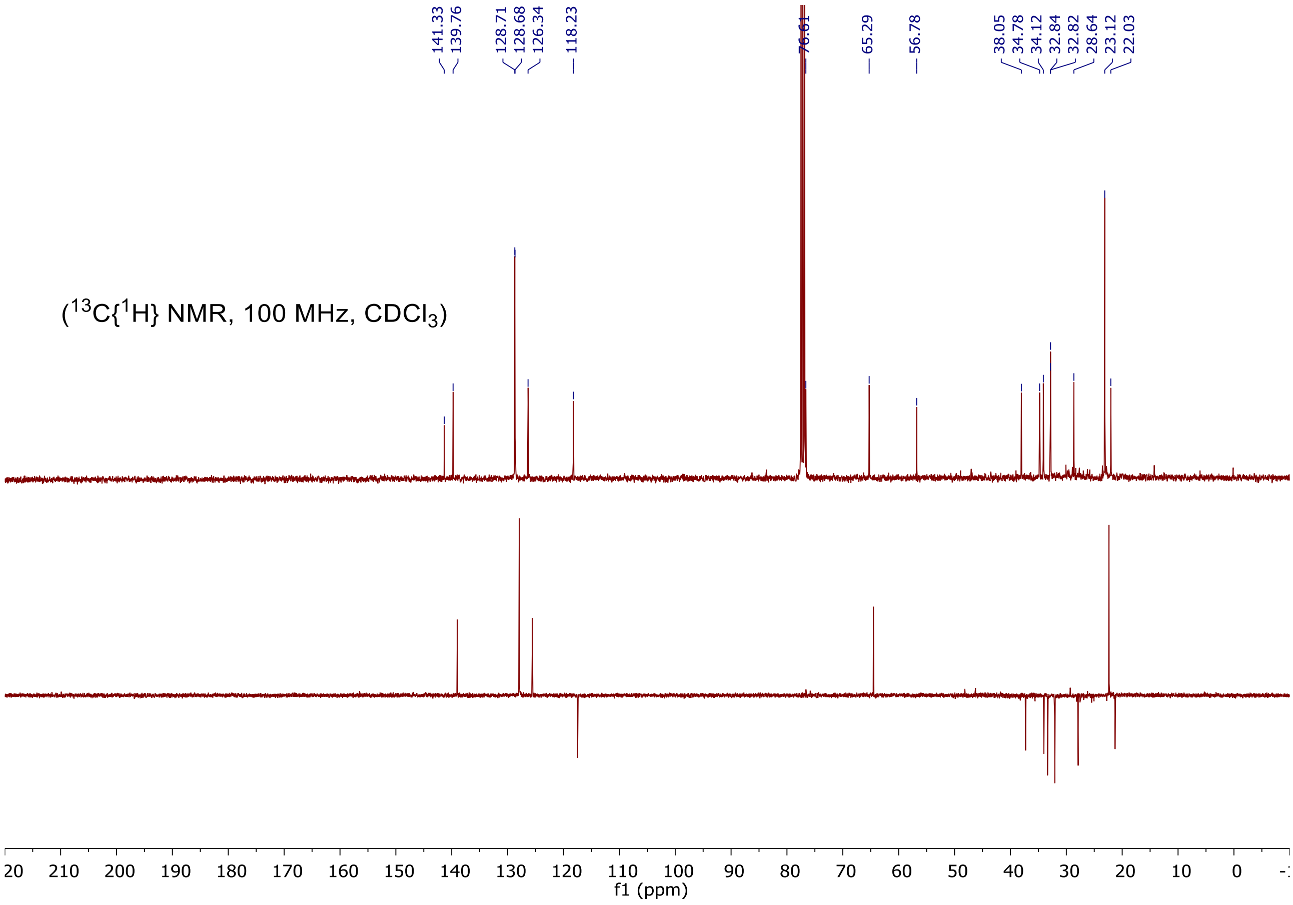

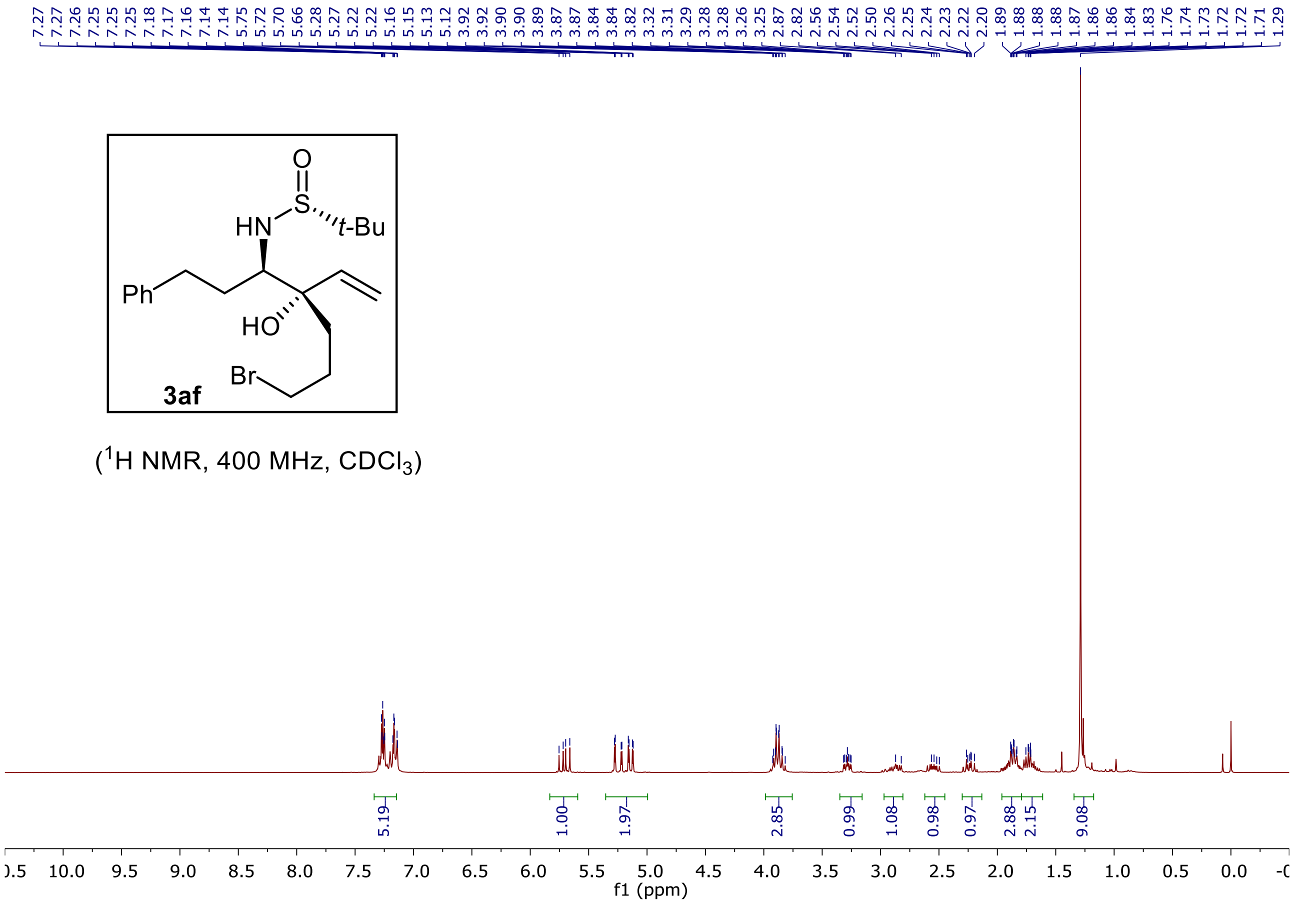
7.27 7.27 7.26 7.25 7.25 7.25 7.18 7.17 7.16 7.14 5.75 5.72 5.70 5.66 5.28 5.27 5.22 5.22 5.16 5.15 5.13 5.12 3.92 3.92 3.90 3.90 3.89 3.87 3.87 3.84 3.84 3.82 3.32 3.31 3.29 3.28 3.28 3.26 3.25 2.87 2.82 2.56 2.54 2.52 2.50 2.26 2.25 2.24 2.23 2.22 2.20 1.89 1.88 1.88 1.88 1.87 1.86 1.86 1.84 1.83 1.76 1.74 1.73 1.72 1.72 1.71 1.29
O
S
HN
t-Bu
Ph
HO
Br
3af
(1H NMR, 400 MHz, CDCl3)
5.19 1.00 1.97 2.85 0.99 1.08 0.98 0.97 2.88 2.15 9.08
f1 (ppm)

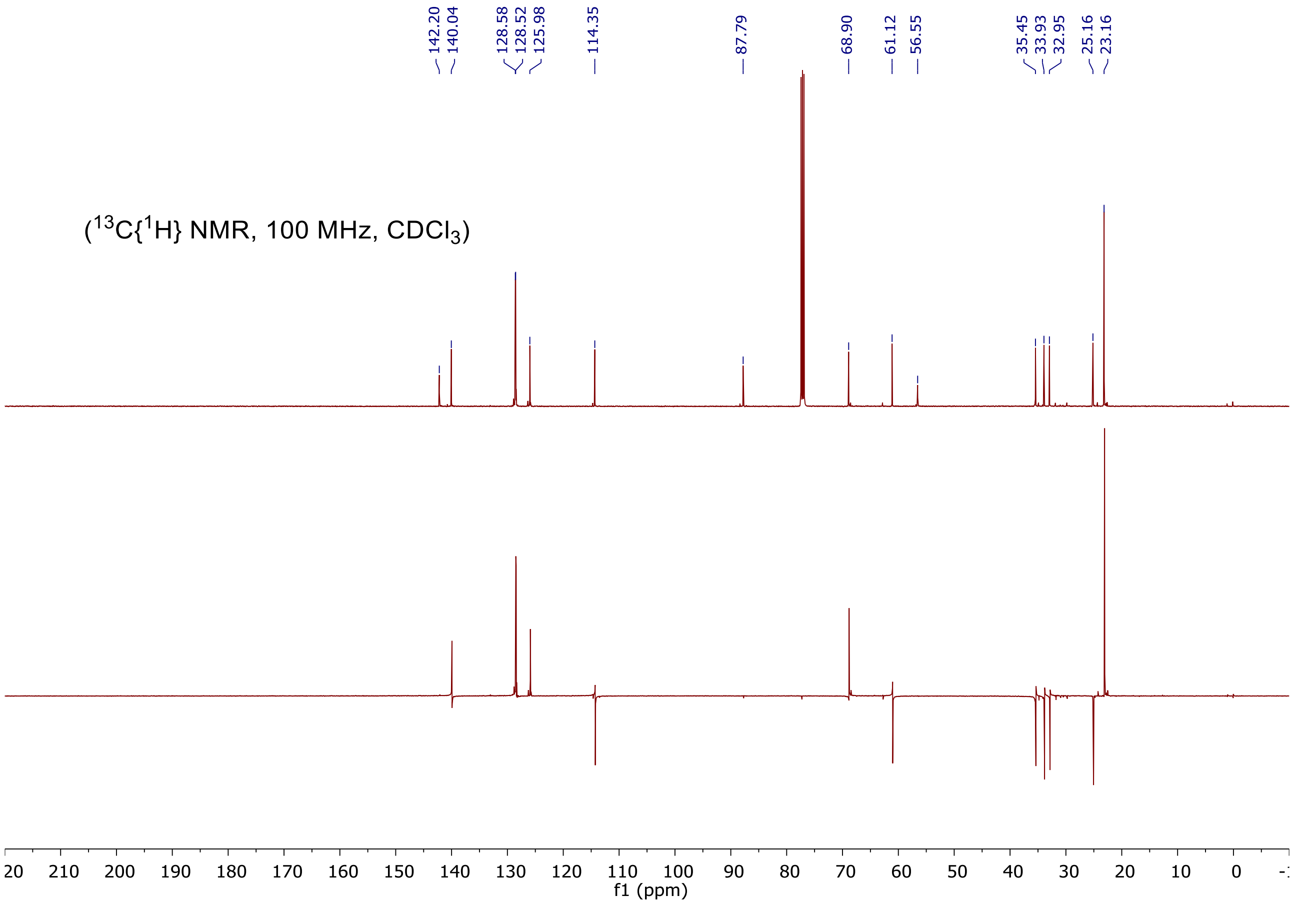
142.20 140.04 128.58 128.52 125.98 114.35 87.79 68.90 61.12 56.55 35.45 33.93 32.95 25.16 23.16
(13C{1H} NMR, 100 MHz, CDCl3)
f1 (ppm)

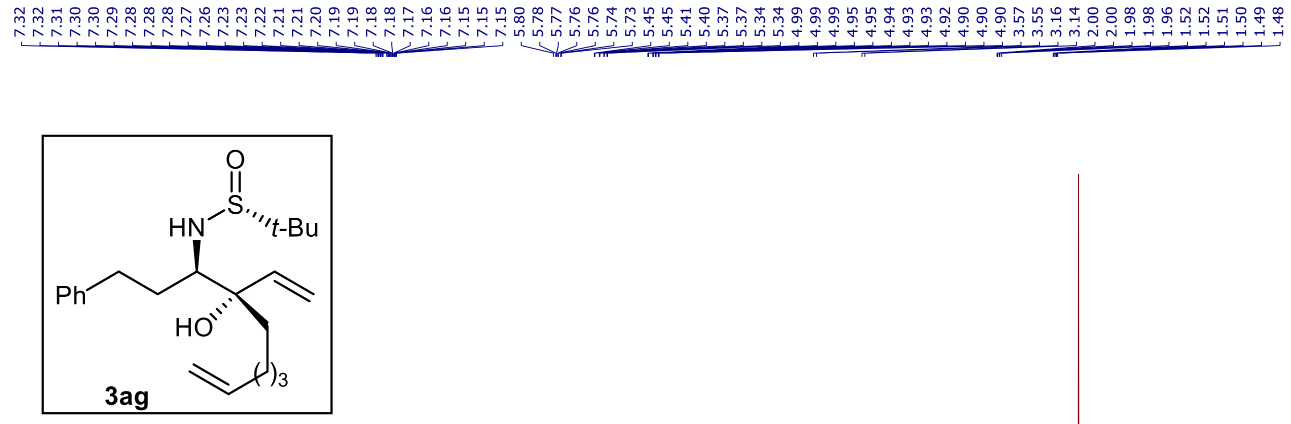

3ag
HN
S
O
t-Bu
Ph
HO
3

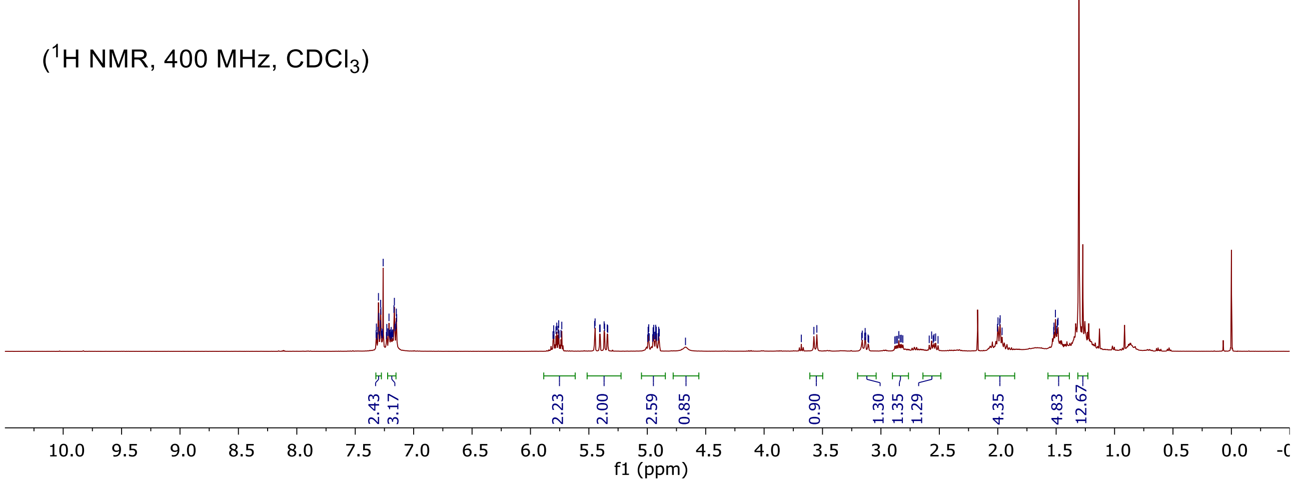

7.32 7.32 7.31 7.30 7.30 7.29 7.28 7.28 7.28 7.27 7.26 7.23 7.23 7.22 7.21 7.21 7.20 7.19 7.19 7.18 7.18 7.17 7.16 7.15 7.15 7.15 5.80 5.78 5.77 5.76 5.76 5.74 5.73 5.45 5.45 5.41 5.40 5.37 5.37 5.34 5.34 4.99 4.99 4.95 4.95 4.94 4.93 4.93 4.92 4.90 4.90 3.57 3.55 3.16 3.14 2.00 2.00 1.98 1.96 1.52 1.51 1.50 1.49 1.48
(1H NMR, 400 MHz, CDCl3)
2.43 3.17 2.23 2.00 2.59 0.85 0.90 1.30 1.35 1.29 4.35 4.83 12.67
10.0 9.5 9.0 8.5 8.0 7.5 7.0 6.5 6.0 5.5 5.0 4.5 4.0 3.5 3.0 2.5 2.0 1.5 1.0 0.5 0.0
f1 (ppm)

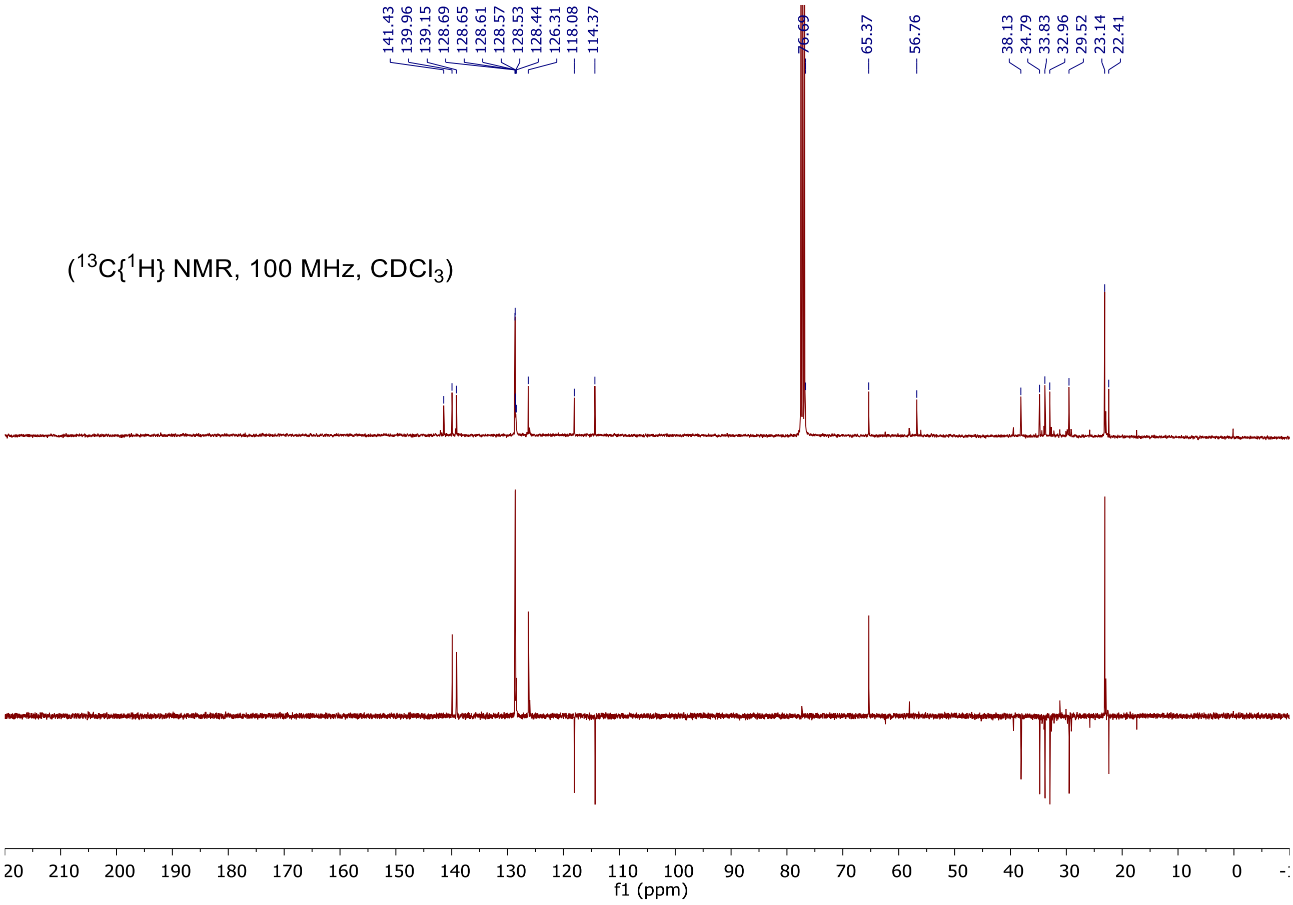

141.43 139.96 139.15 128.69 128.65 128.61 128.57 128.53 128.44 126.31 118.08 114.37
[illegible]
65.37
56.76
38.13 34.79 33.83 32.96 29.52 23.14 22.41
(13C{1H} NMR, 100 MHz, CDCl3)
210 200 190 180 170 160 150 140 130 120 110 100 90 80 70 60 50 40 30 20 10 0
f1 (ppm)

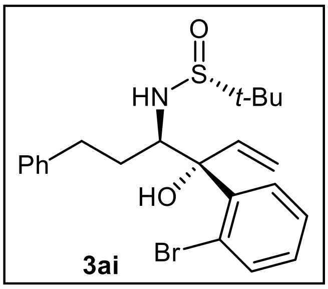

3ai

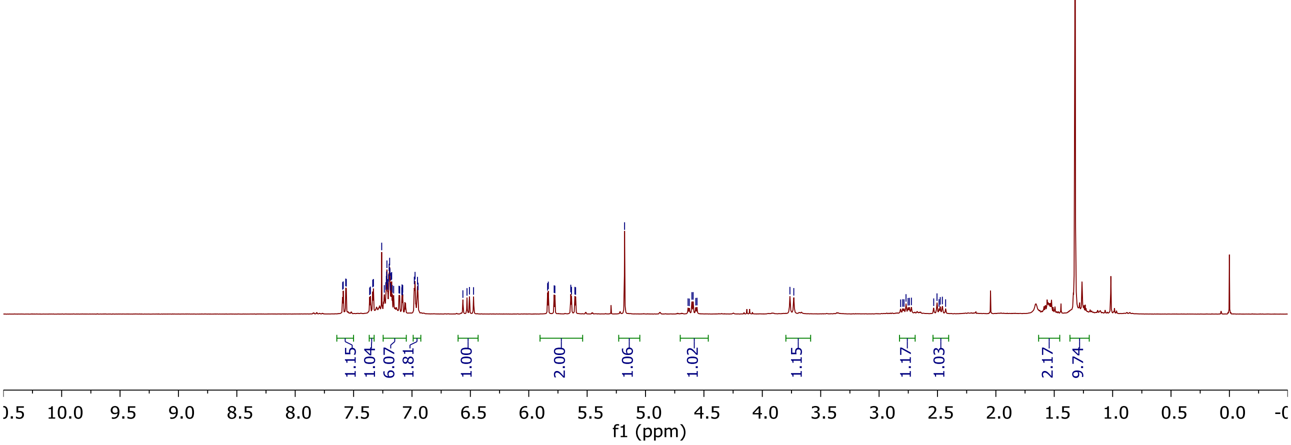

(^{1}H NMR, 400 MHz, $CDCl_3$)

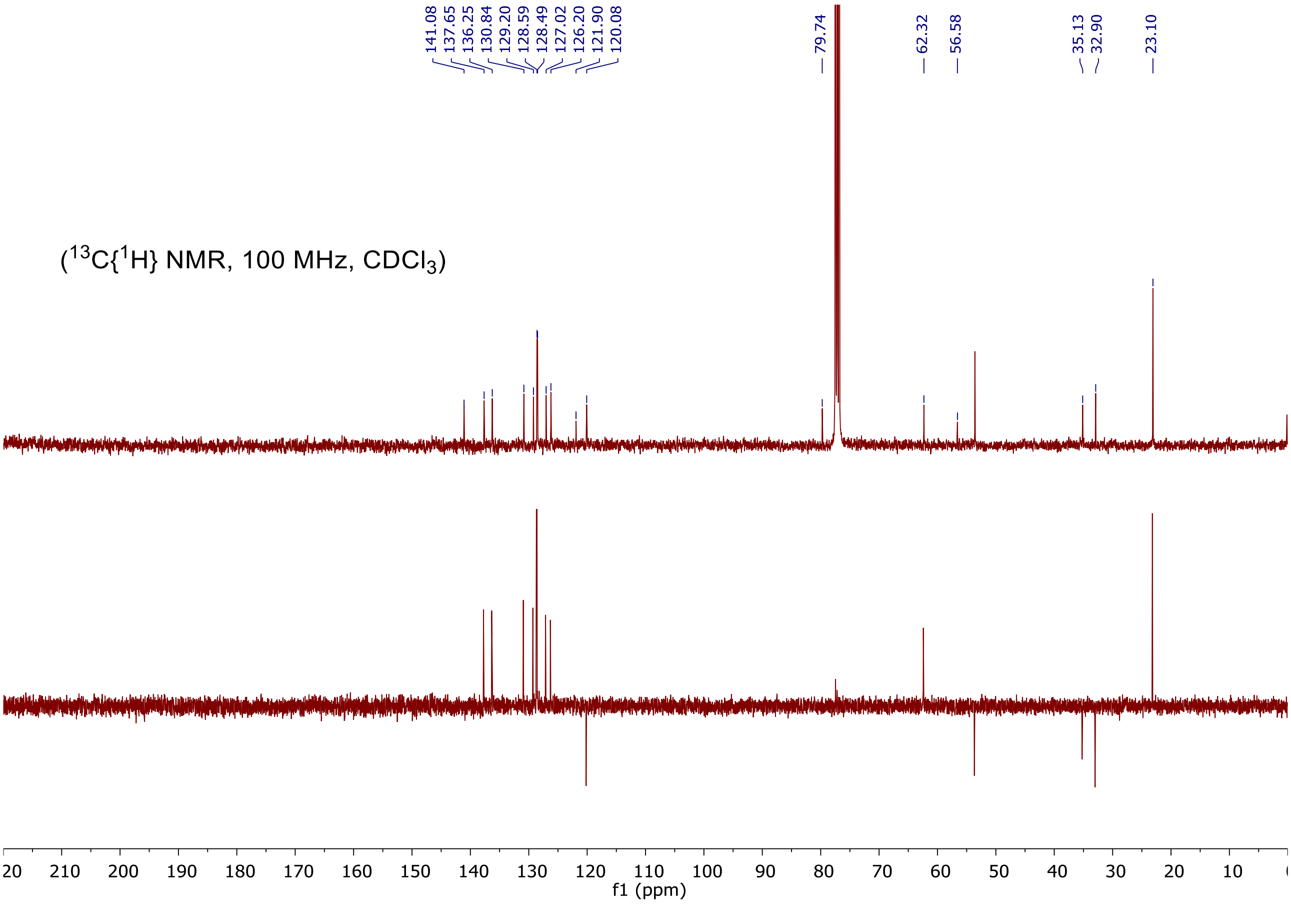

(^{13}C{^{1}H} NMR, 100 MHz, $CDCl_3$)

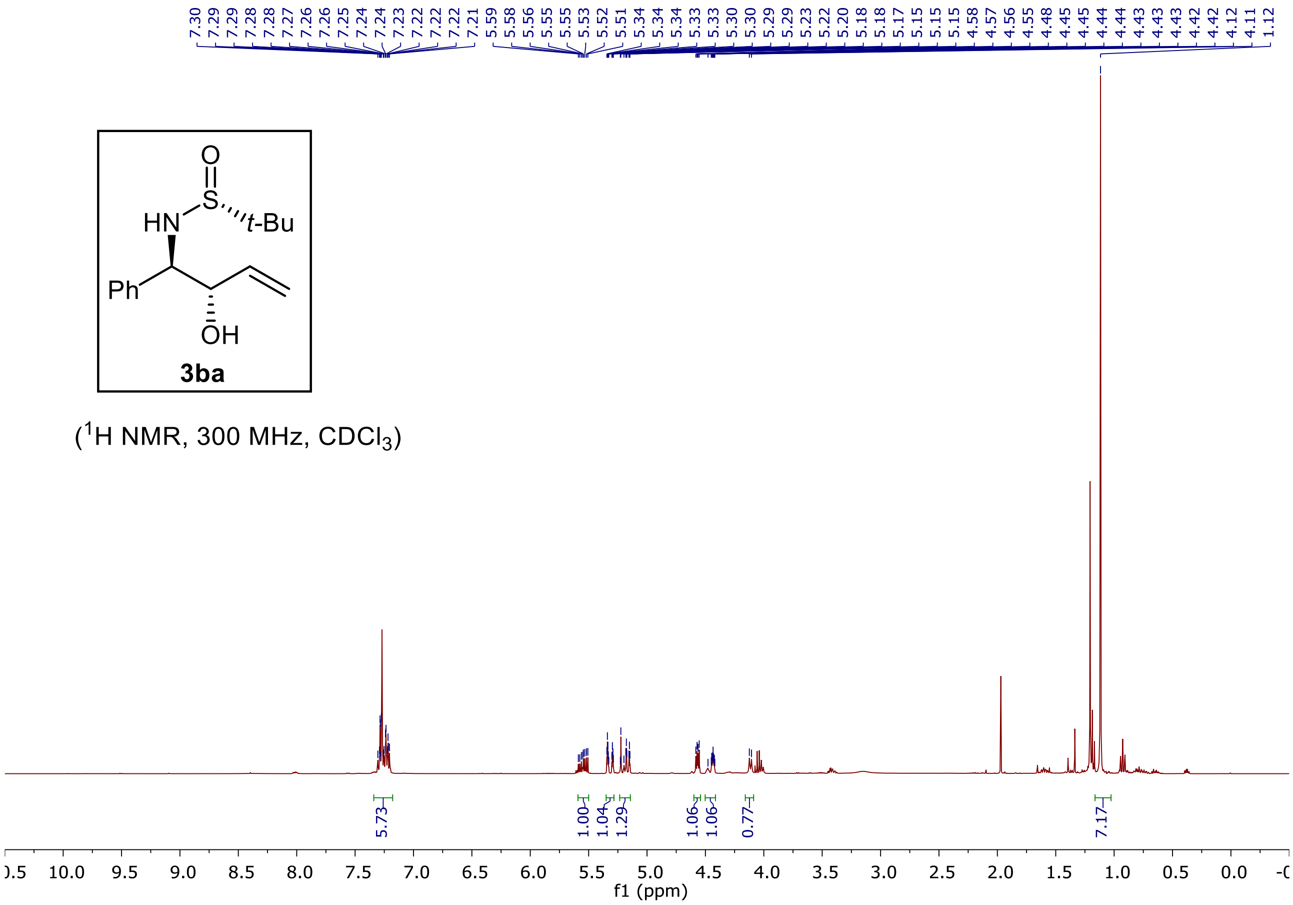

7.30 7.29 7.29 7.28 7.28 7.27 7.26 7.26 7.25 7.24 7.24 7.23 7.22 7.22 7.22 7.21 5.59 5.58 5.56 5.55 5.55 5.53 5.52 5.51 5.34 5.34 5.34 5.33 5.33 5.30 5.29 5.29 5.23 5.22 5.20 5.18 5.18 5.17 5.15 5.15 5.15 4.58 4.57 4.56 4.55 4.48 4.45 4.45 4.44 4.44 4.43 4.43 4.43 4.42 4.42 4.12 4.11 1.12
O
S
HN
t-Bu
Ph
OH
3ba
(^{1}H NMR, 300 MHz, $CDCl_3$)
5.73
1.00
1.04
1.29
1.06
1.06
0.77
7.17
f1 (ppm)

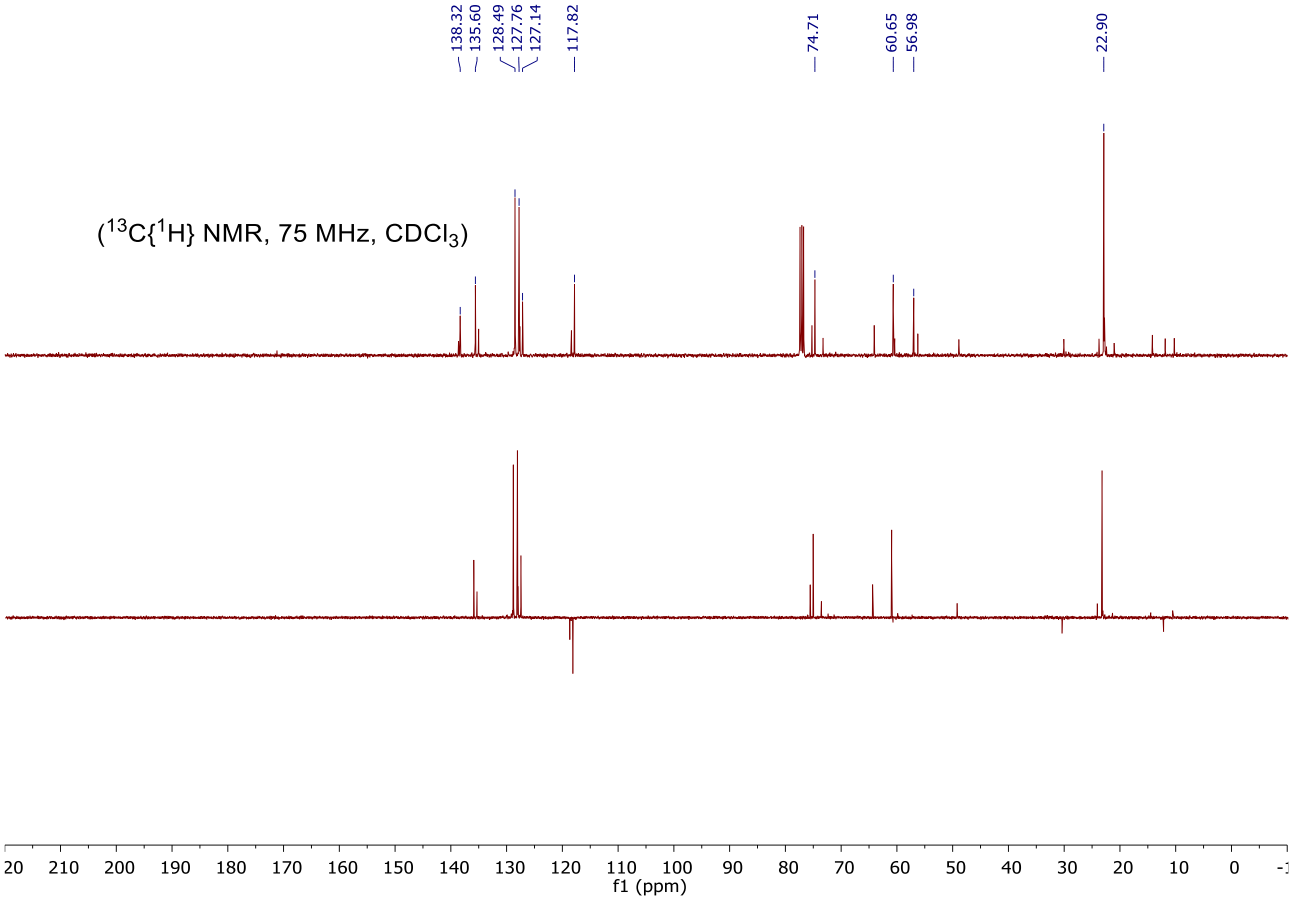

138.32
135.60
128.49
127.76
127.14
117.82
74.71
60.65
56.98
22.90
(^{13}C{^{1}H} NMR, 75 MHz, $CDCl_3$)
f1 (ppm)

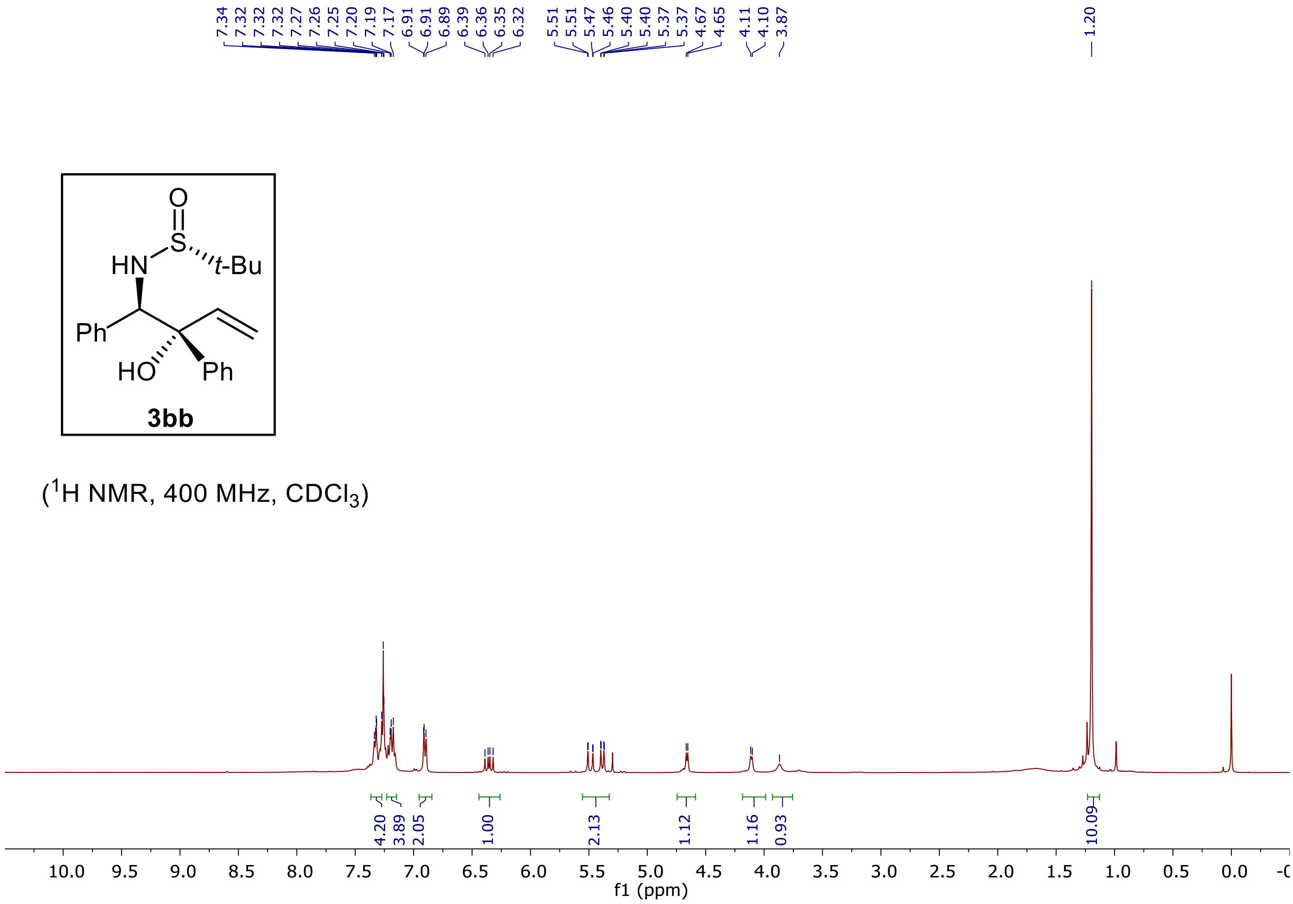

(^{1}H NMR, 400 MHz, $CDCl_3$)

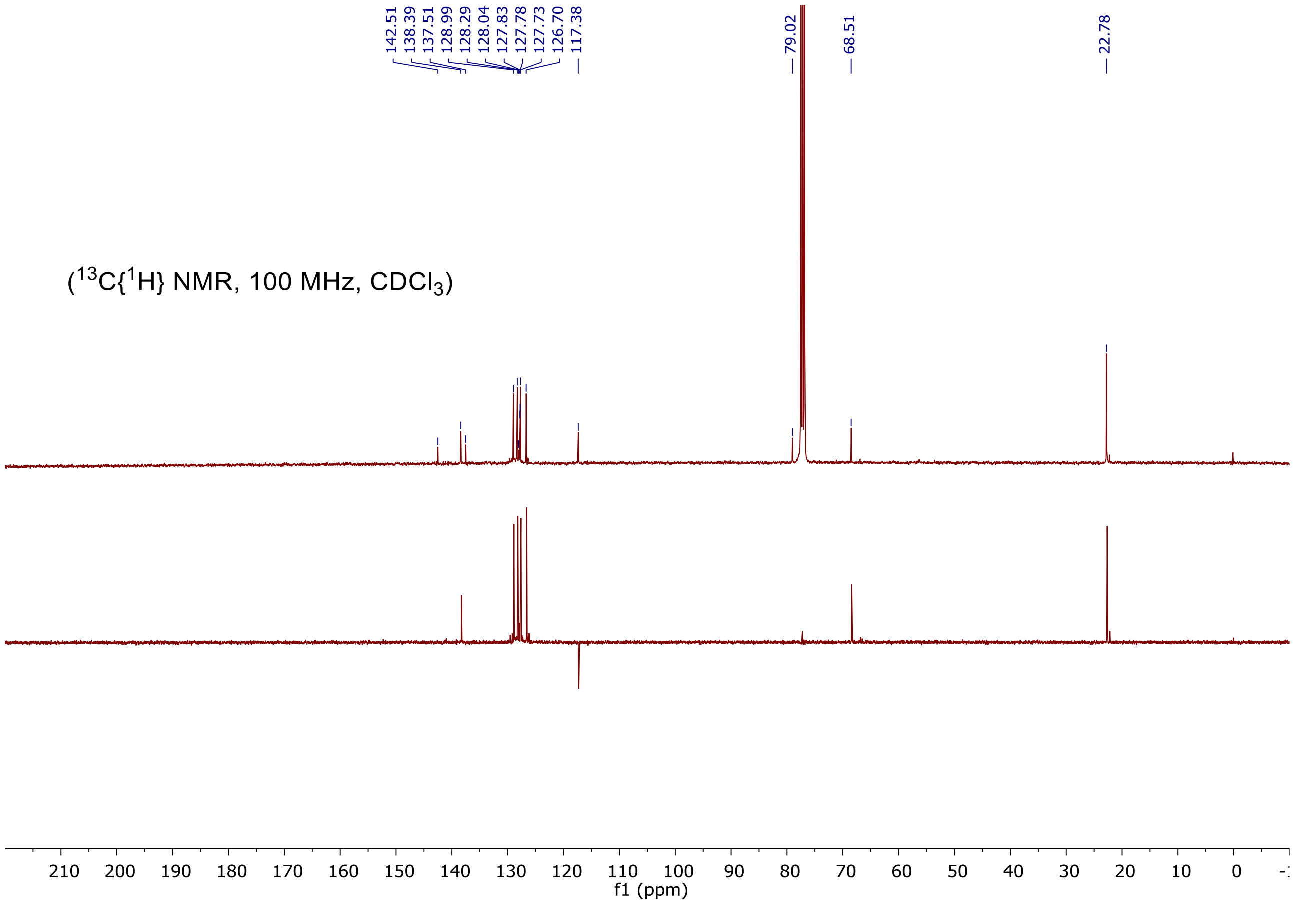

(^{13}C{^{1}H} NMR, 100 MHz, $CDCl_3$)

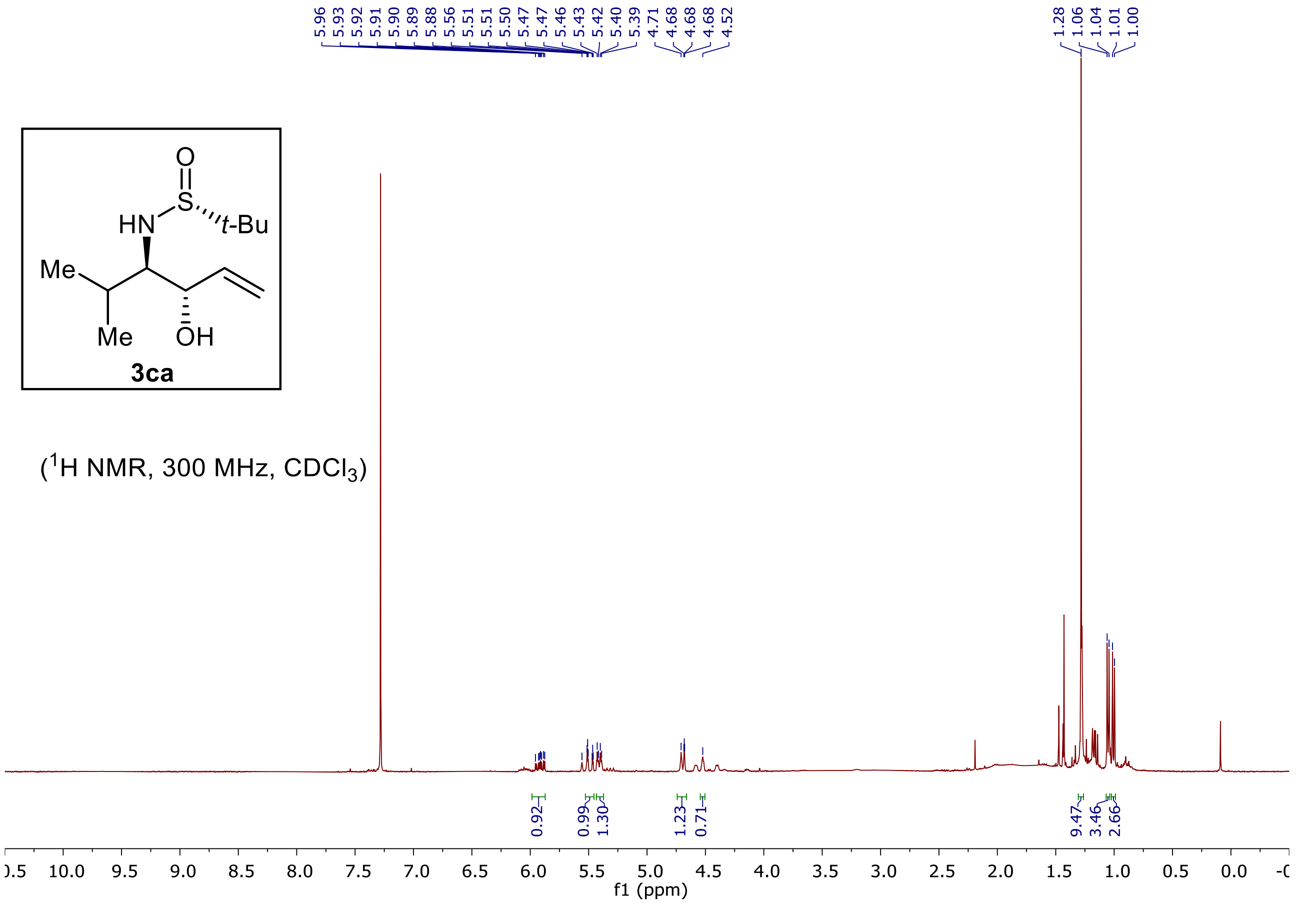
5.96 5.93 5.92 5.91 5.90 5.89 5.88 5.56 5.51 5.51 5.50 5.47 5.47 5.46 5.43 5.42 5.40 5.39 4.71 4.68 4.68 4.68 4.52
1.28 1.06 1.04 1.01 1.00
O
HN
S
t-Bu
Me
Me
OH
3ca
(1H NMR, 300 MHz, CDCl3)
0.92 0.99 1.30 1.23 0.71 9.47 3.46 2.66
f1 (ppm)

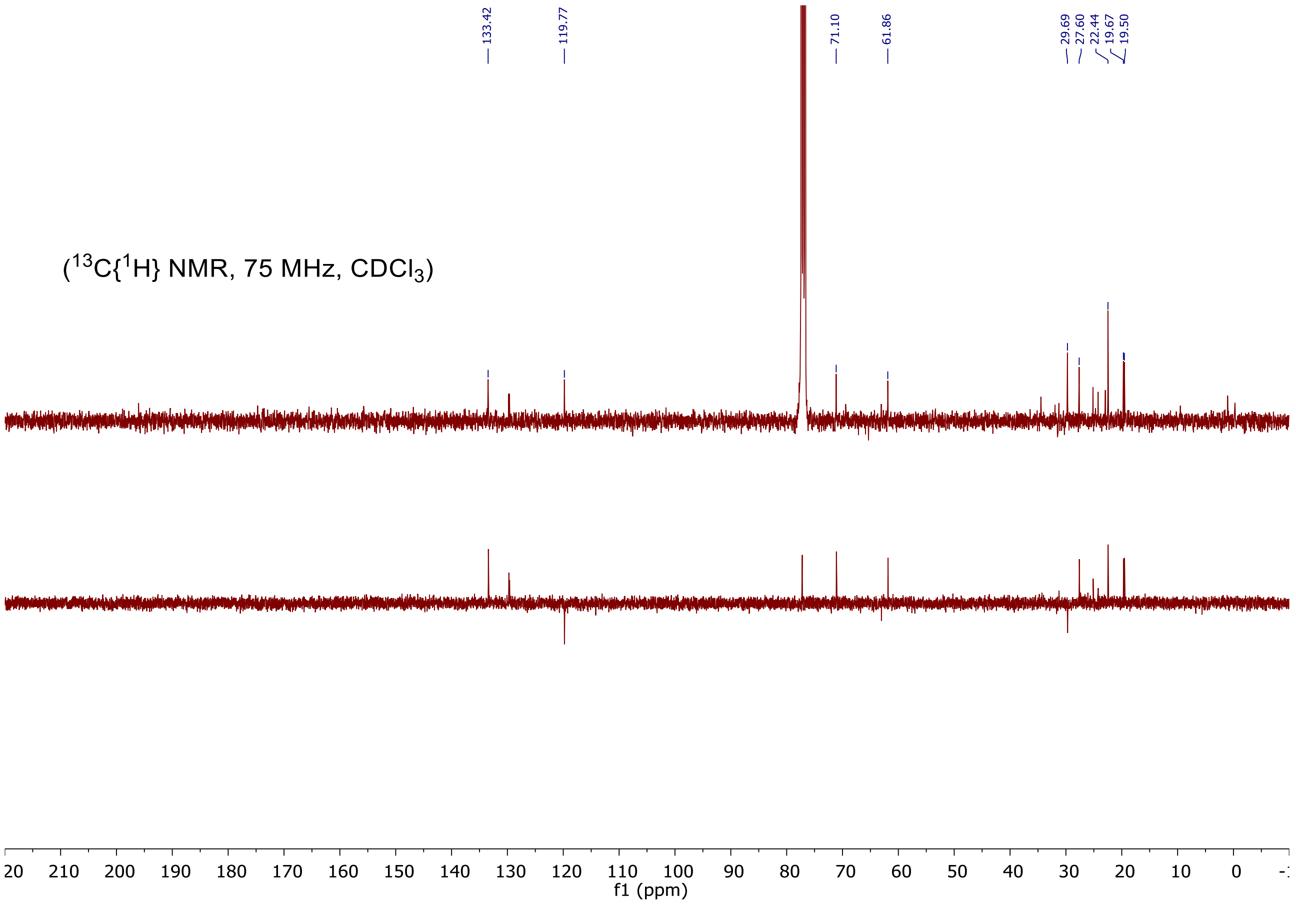
133.42
119.77
71.10
61.86
29.69 27.60 22.44 19.67 19.50
(13C{1H} NMR, 75 MHz, CDCl3)
f1 (ppm)

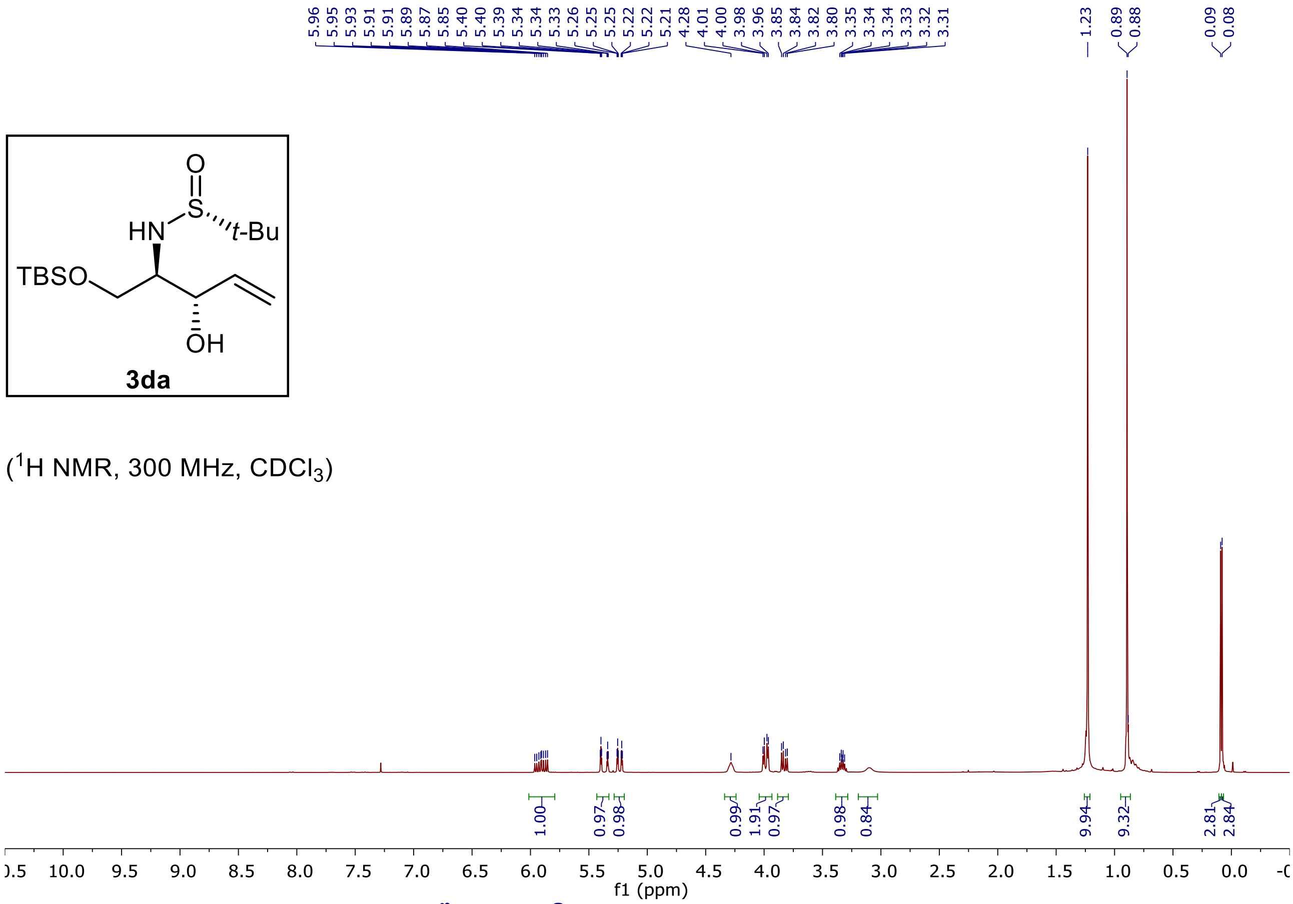

3da

(^{1}H NMR, 300 MHz, $CDCl_3$)

(${}^{13}C\{{}^1H\}$ NMR, 75 MHz, $CDCl_3$)

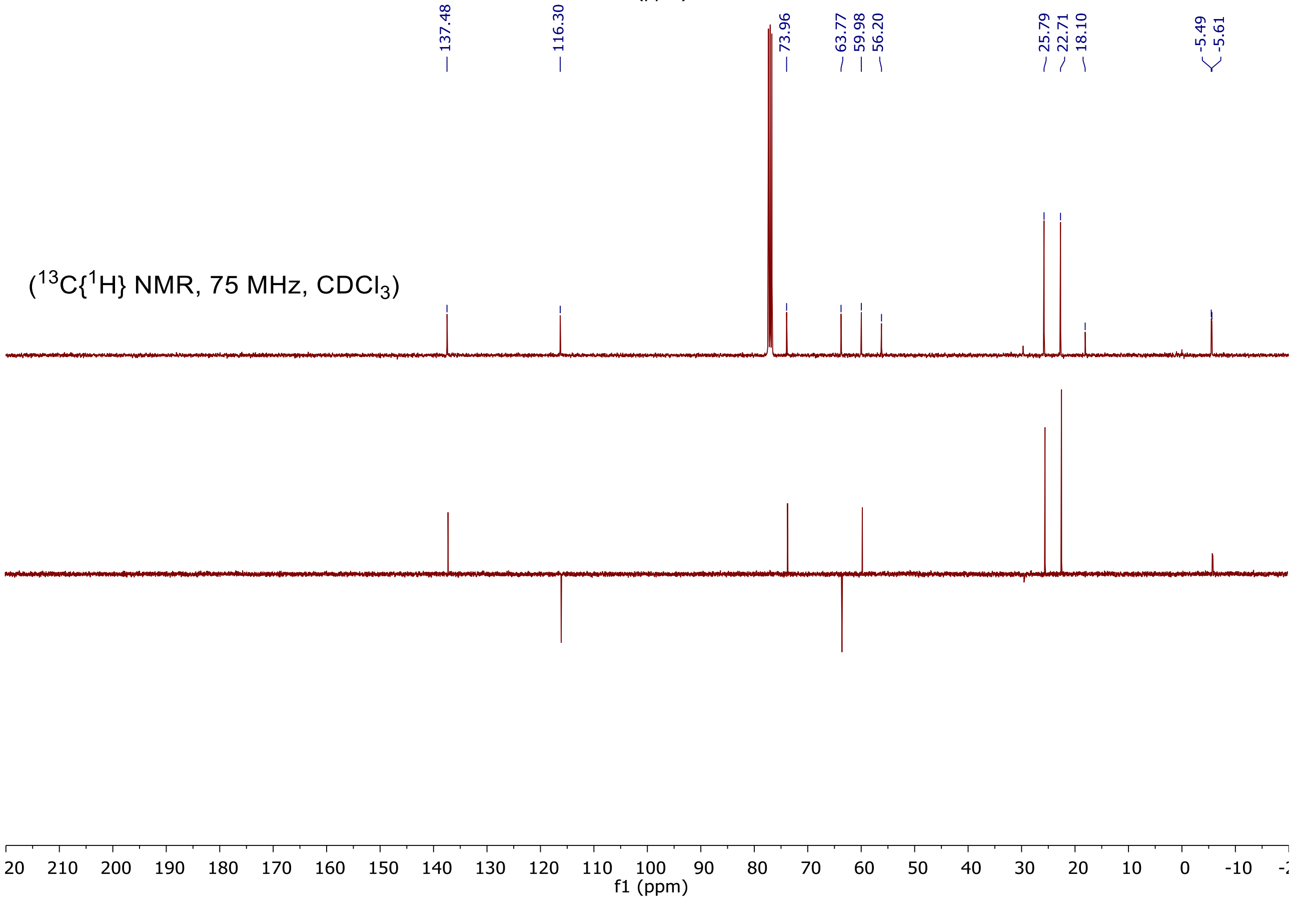

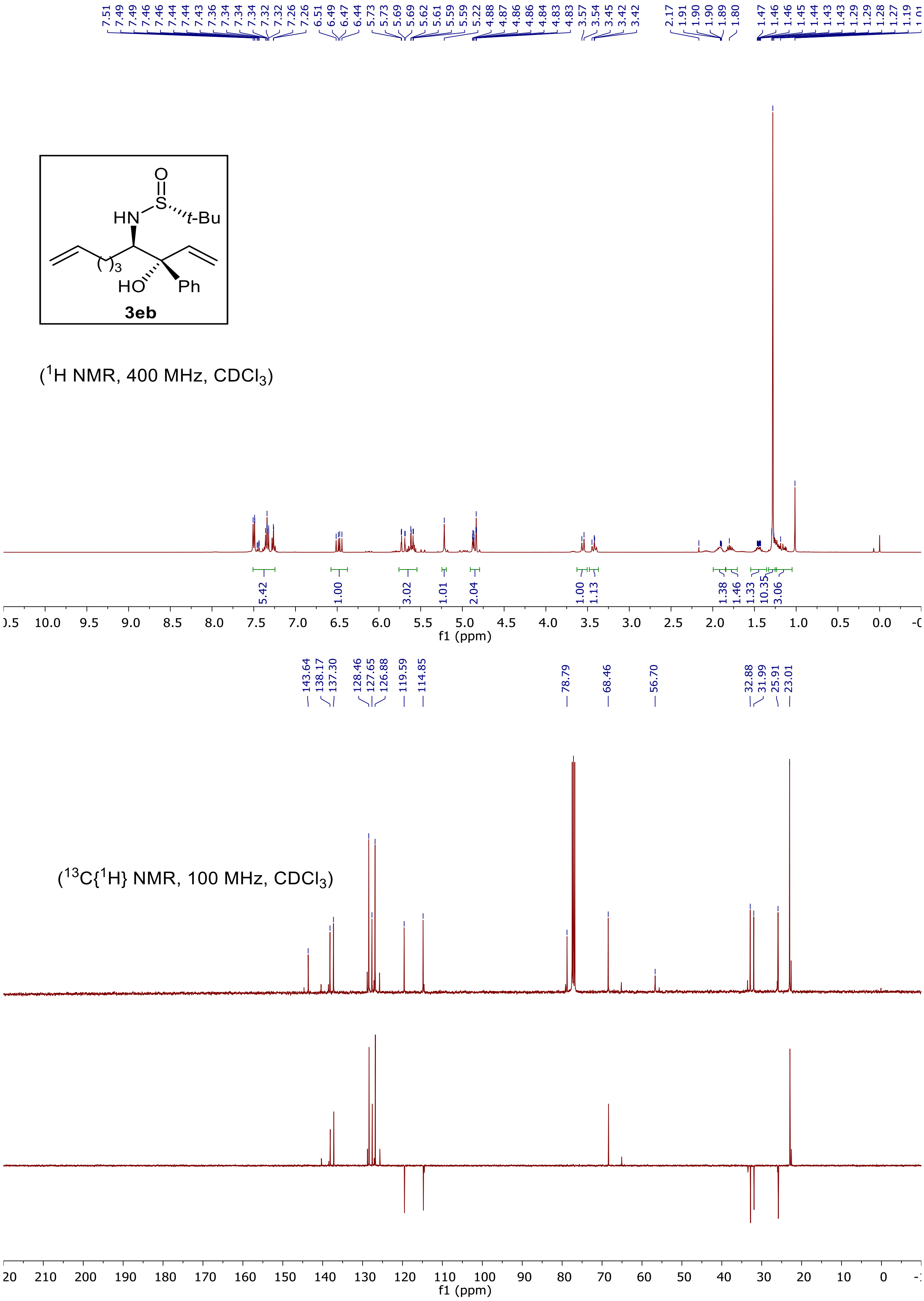
7.51 7.49 7.49 7.46 7.44 7.44 7.43 7.36 7.34 7.34 7.34 7.32 7.32 7.26 7.26 6.51 6.49 6.47 6.44 5.73 5.73 5.69 5.69 5.62 5.61 5.59 5.59 5.22 4.88 4.87 4.86 4.86 4.84 4.83 4.83 3.57 3.54 3.45 3.42 3.42 2.17 1.91 1.90 1.90 1.89 1.80 1.47 1.46 1.46 1.45 1.44 1.43 1.29 1.29 1.28 1.27 1.19
O
S
HN
t-Bu
3
HO
Ph
3eb
(1H NMR, 400 MHz, CDCl3)
5.42
1.00
3.02
1.01
2.04
1.00
1.13
1.38
1.46
1.33
10.35
3.06
f1 (ppm)
143.64 138.17 137.30 128.46 127.65 126.88 119.59 114.85 78.79 68.46 56.70 32.88 31.99 25.91 23.01
(13C{1H} NMR, 100 MHz, CDCl3)
f1 (ppm)

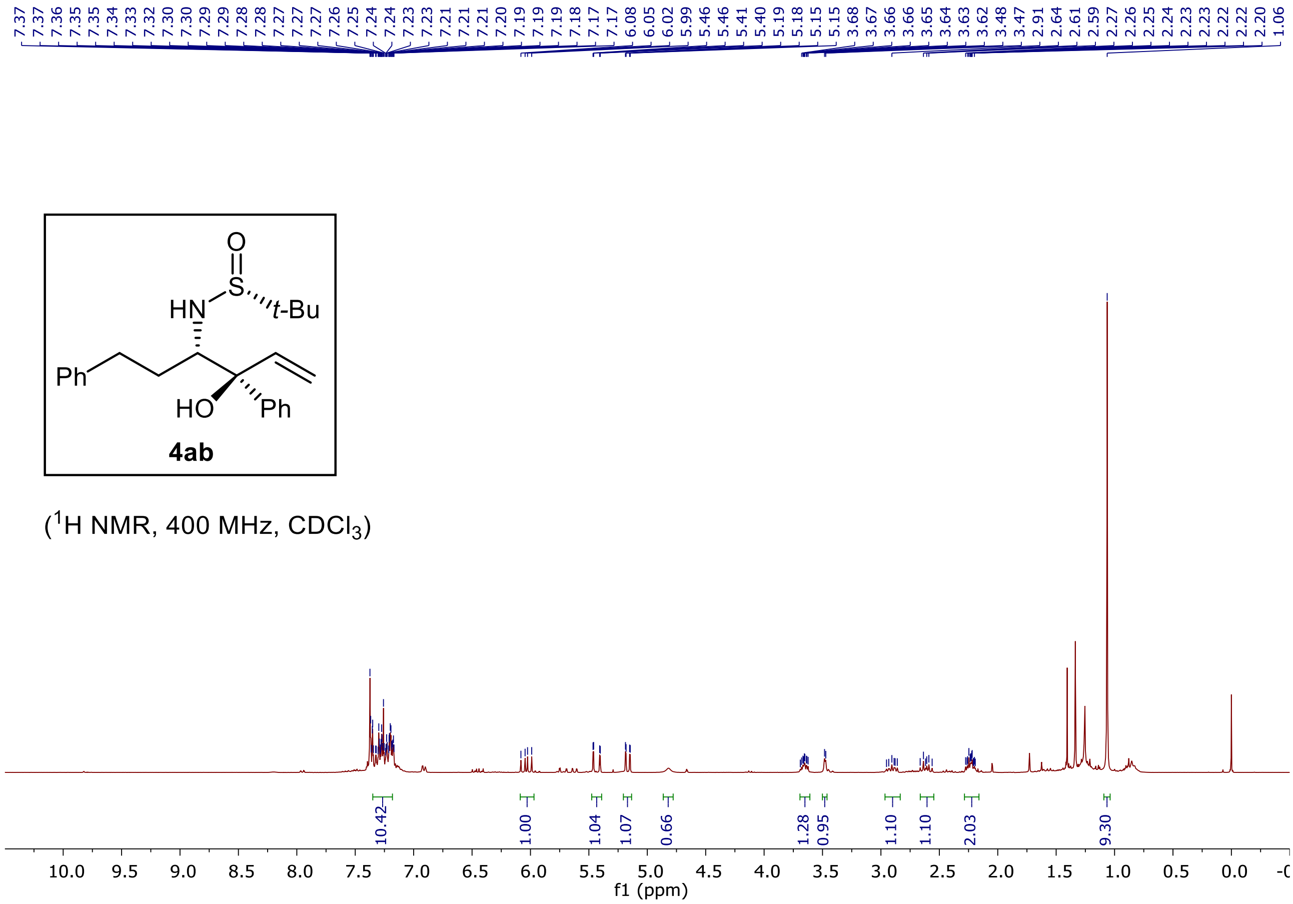

(¹H NMR, 400 MHz, CDCl₃)

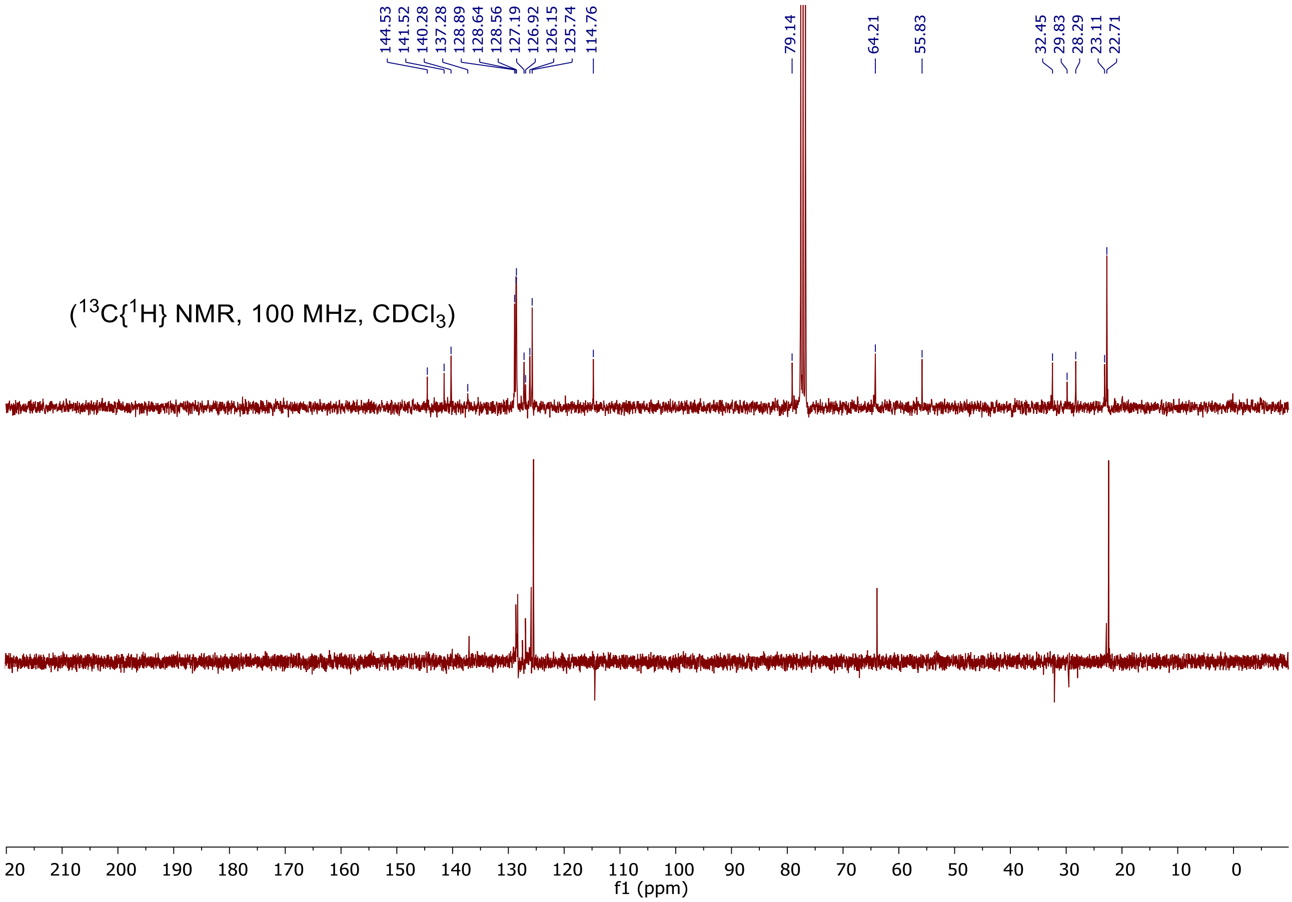

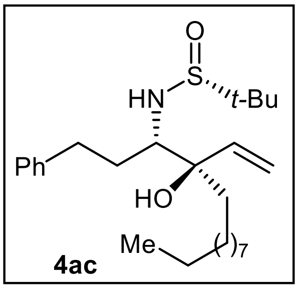

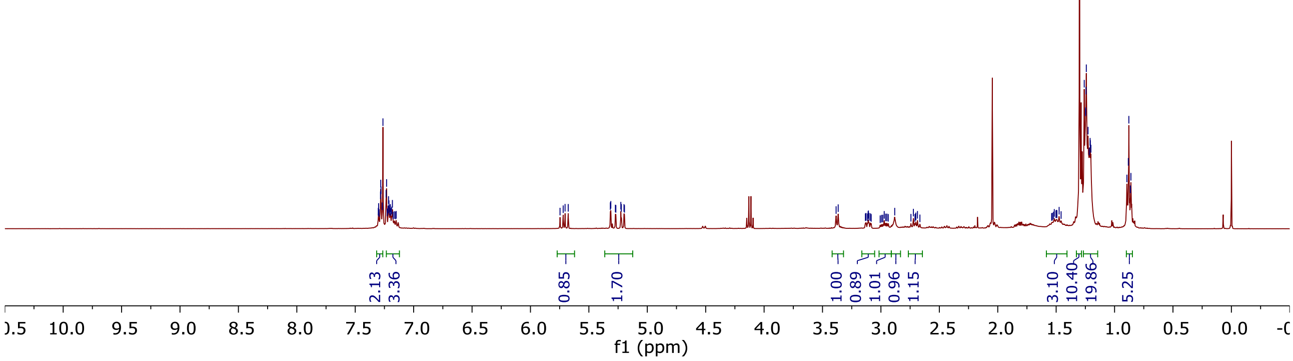

(^{1}H NMR, 400 MHz, $CDCl_3$)

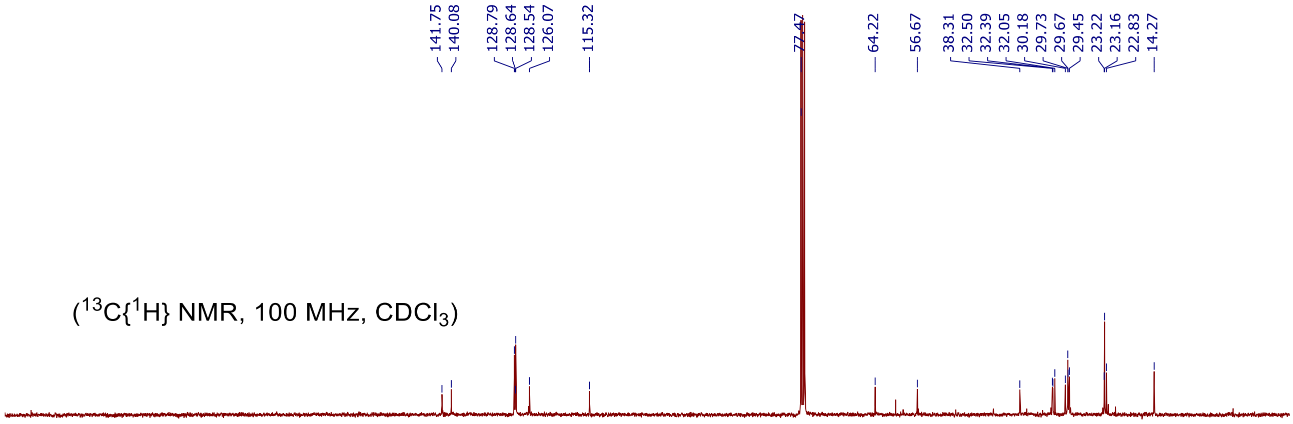

(^{13}C{^{1}H} NMR, 100 MHz, $CDCl_3$)

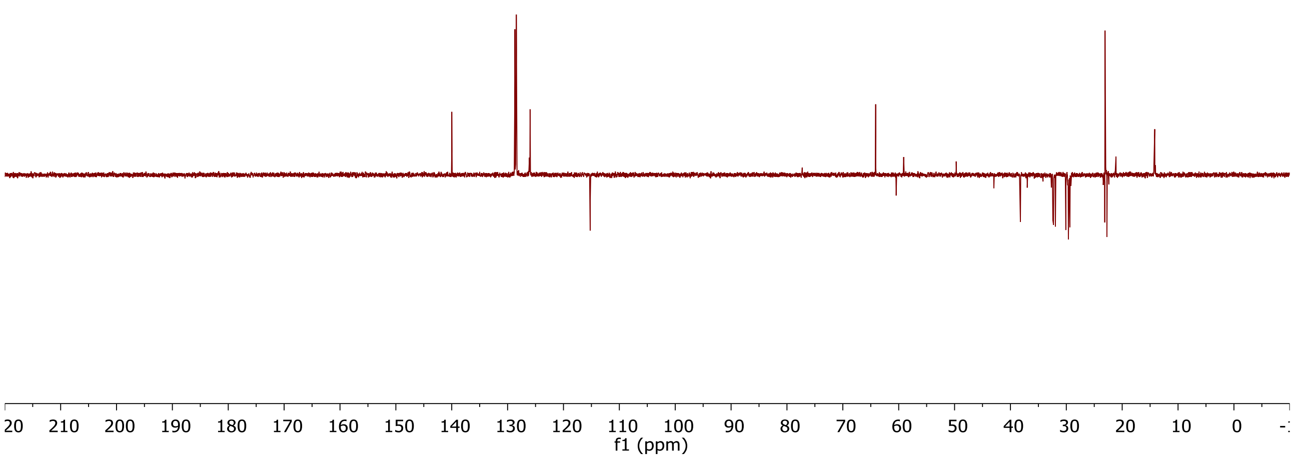

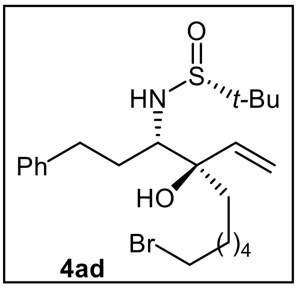

4ad

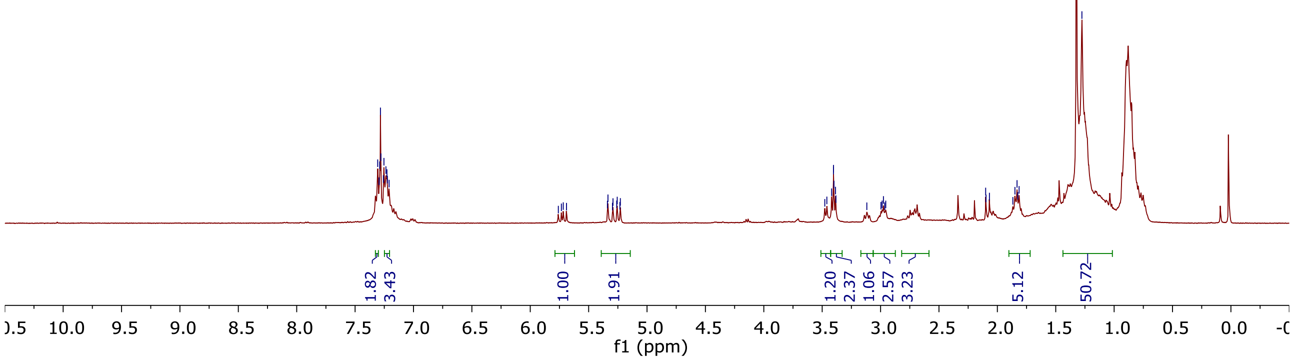

(^{1}H NMR, 400 MHz, $CDCl_3$)

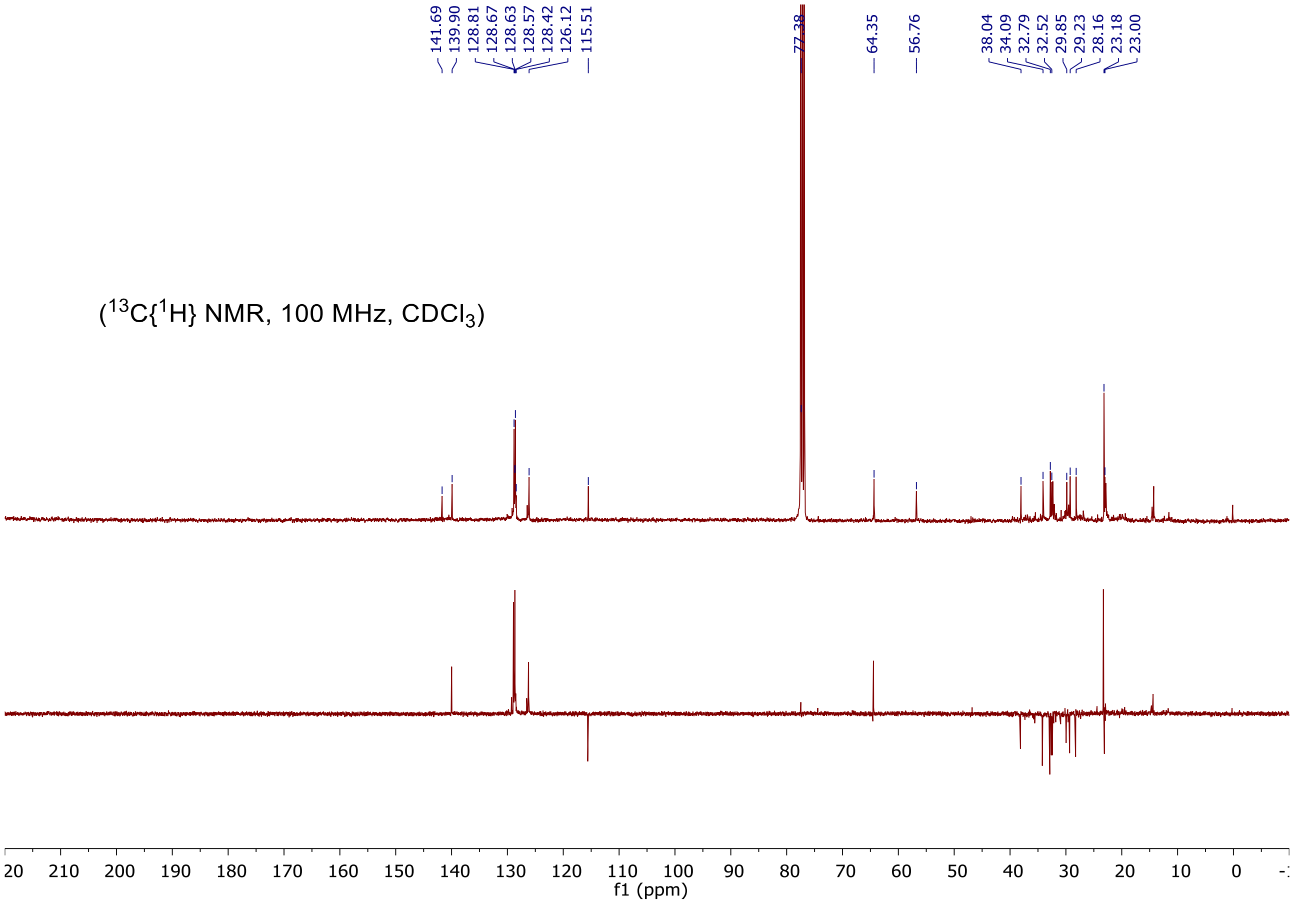

($^{13}C\{^1H\}$ NMR, 100 MHz, $CDCl_3$)

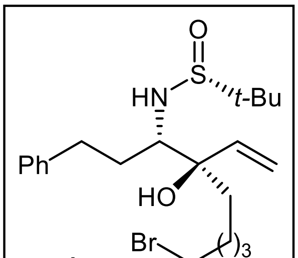

4ae

(¹H NMR, 400 MHz, $CDCl_3$)

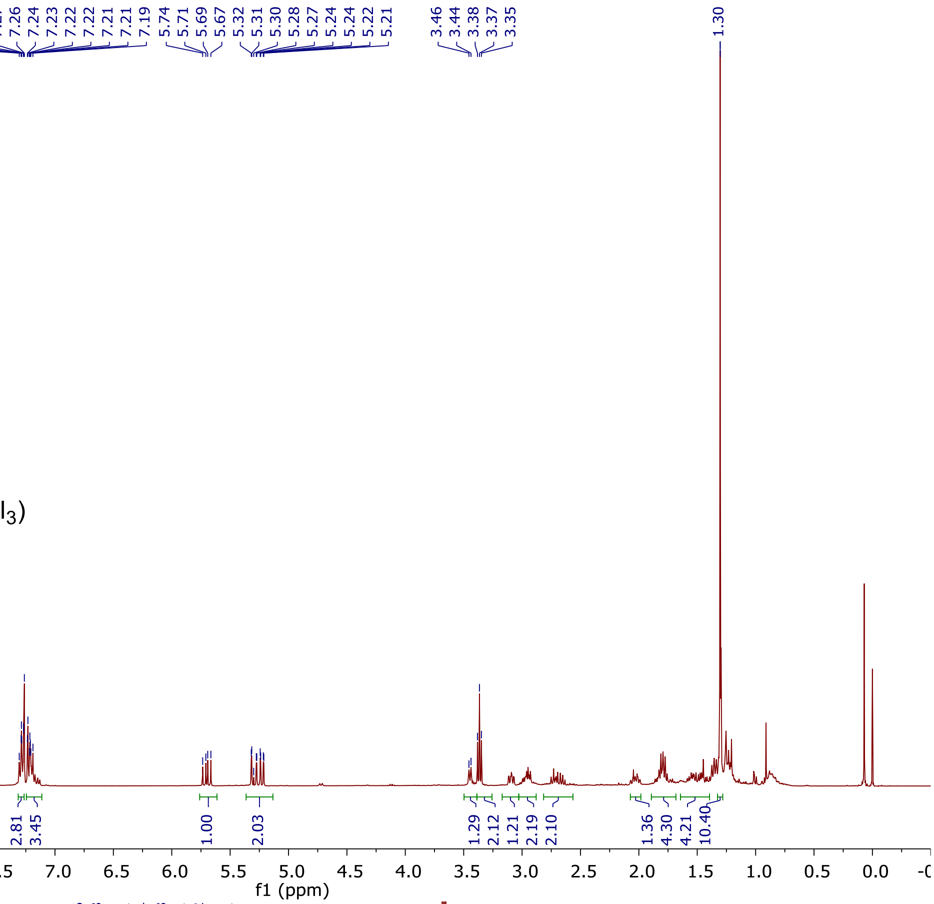

($^{13}C\{^1H\}$ NMR, 100 MHz, $CDCl_3$)

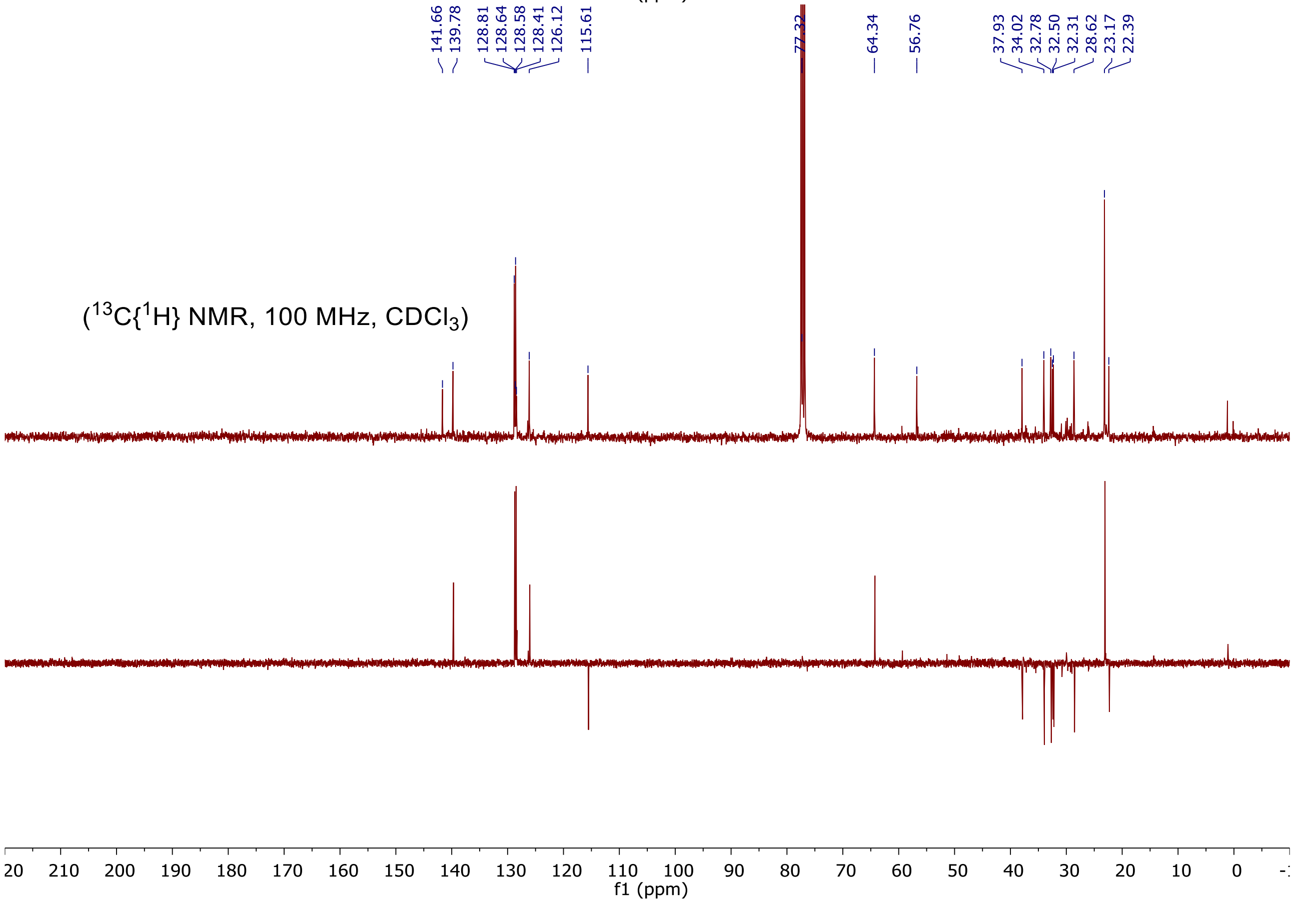

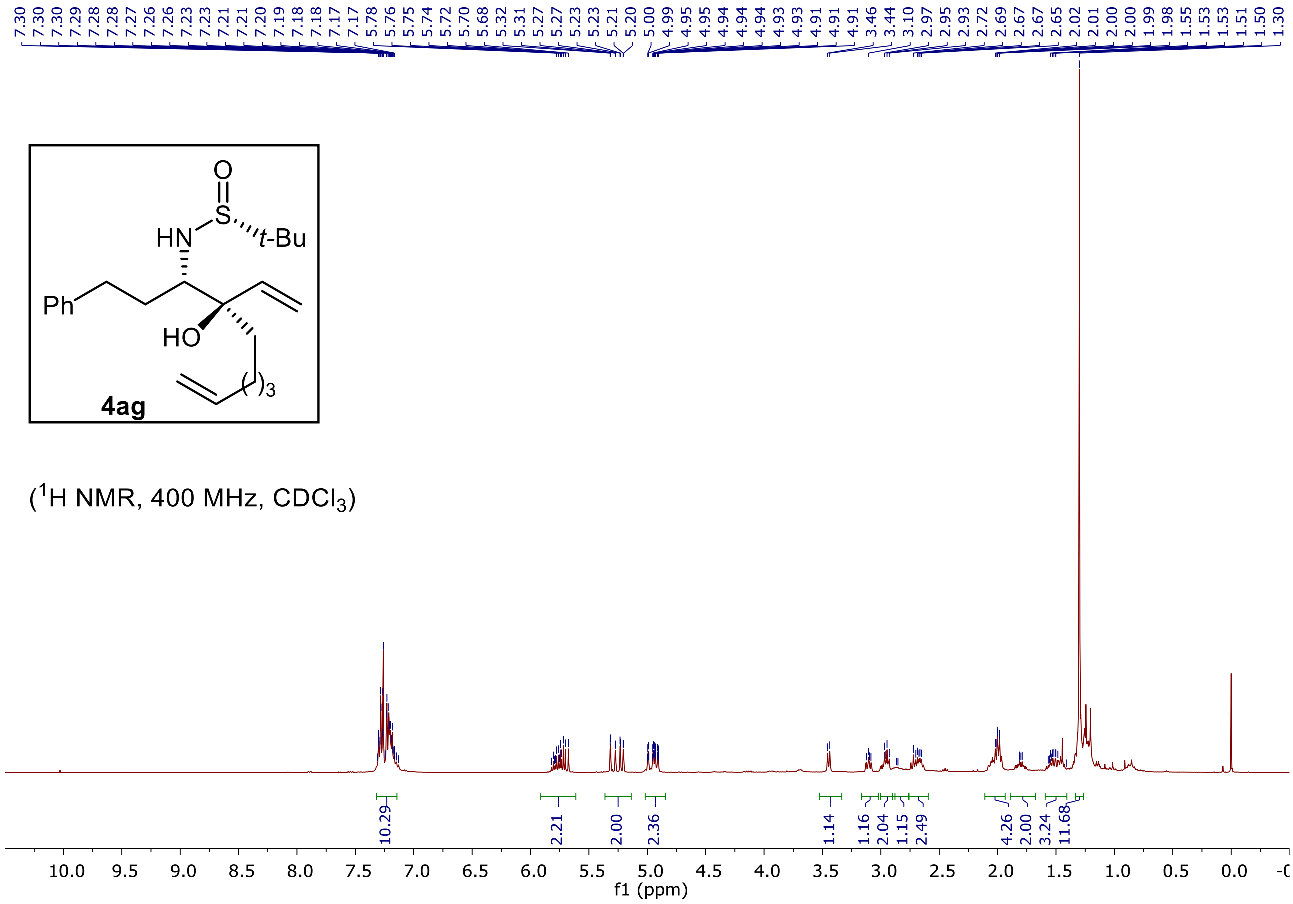

4ag

(^{1}H NMR, 400 MHz, CDCl$_3$)

(^{13}C{^{1}H} NMR, 100 MHz, CDCl$_3$)

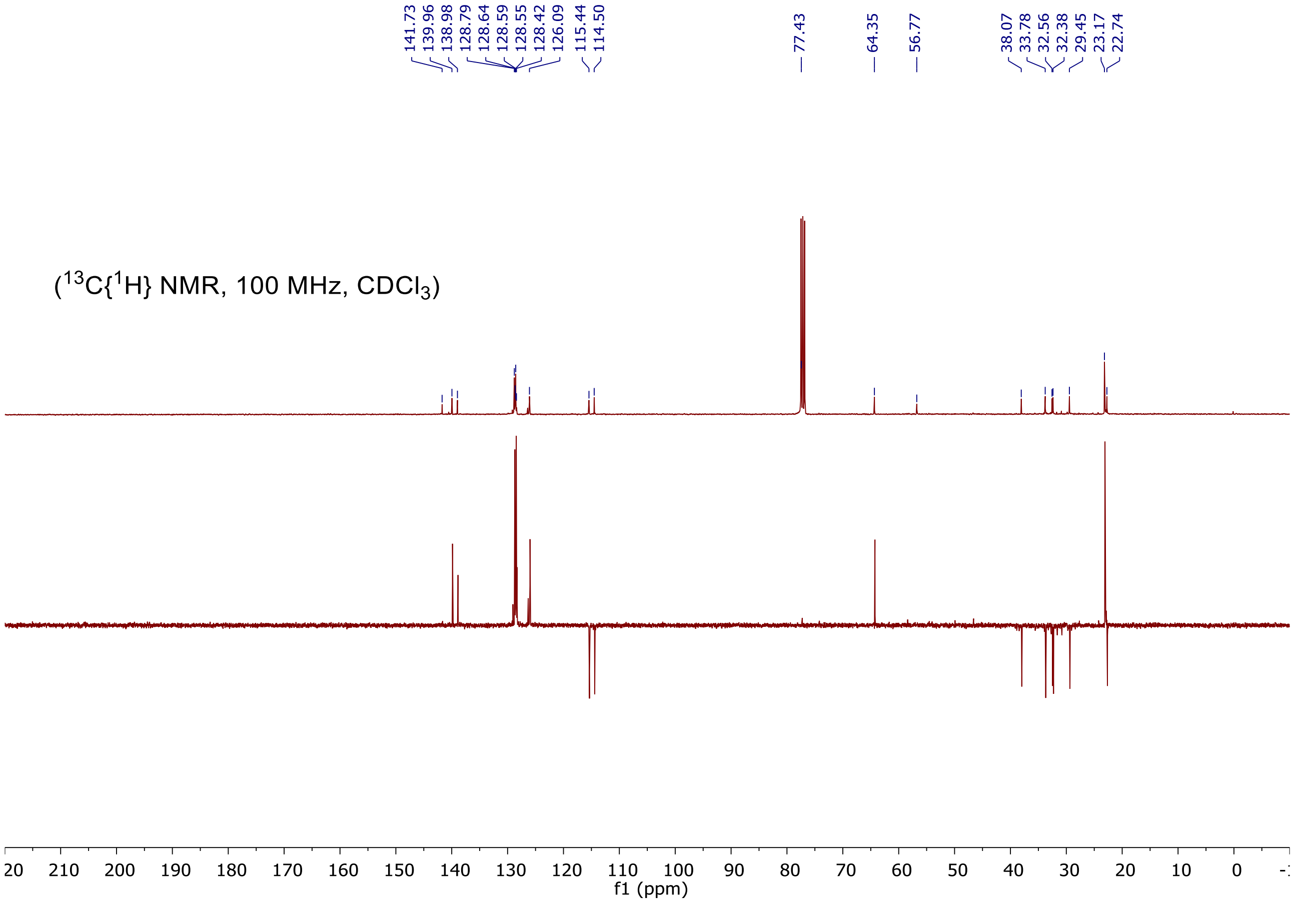

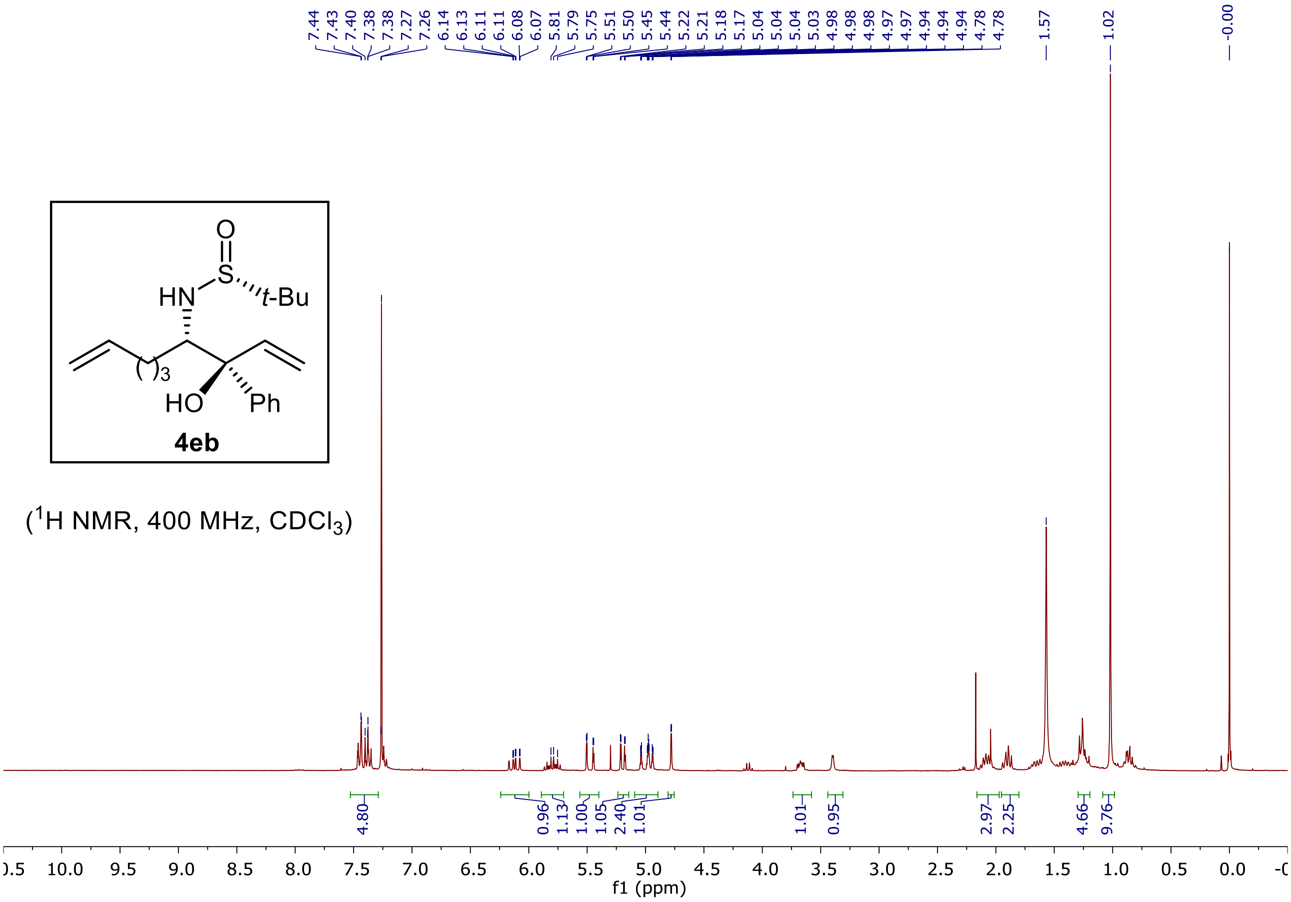
O
S
HN
t-Bu
)3
HO
Ph
4eb
(1H NMR, 400 MHz, CDCl3)
7.44 7.43 7.40 7.38 7.38 7.27 7.26 6.14 6.13 6.11 6.11 6.08 6.07 5.81 5.79 5.75 5.51 5.50 5.45 5.44 5.22 5.21 5.18 5.17 5.04 5.04 5.04 5.03 4.98 4.98 4.97 4.97 4.94 4.94 4.94 4.94 4.78 4.78
1.57
1.02
-0.00
4.80
0.96 1.13 1.00 1.05 2.40 1.01
1.01
0.95
2.97 2.25
4.66
9.76
f1 (ppm)

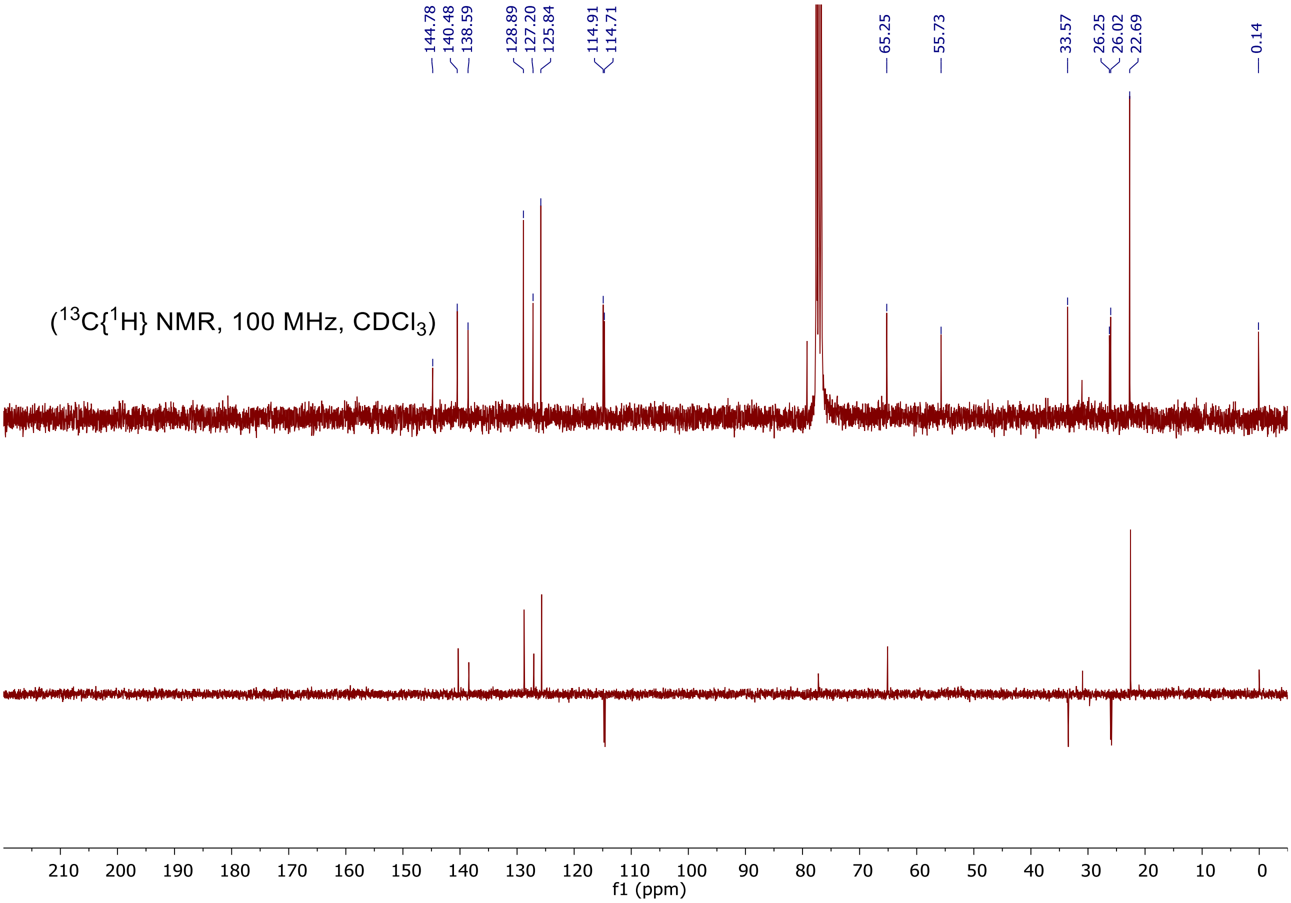
(13C{1H} NMR, 100 MHz, CDCl3)
144.78 140.48 138.59
128.89 127.20 125.84
114.91 114.71
65.25
55.73
33.57
26.25 26.02 22.69
0.14
f1 (ppm)

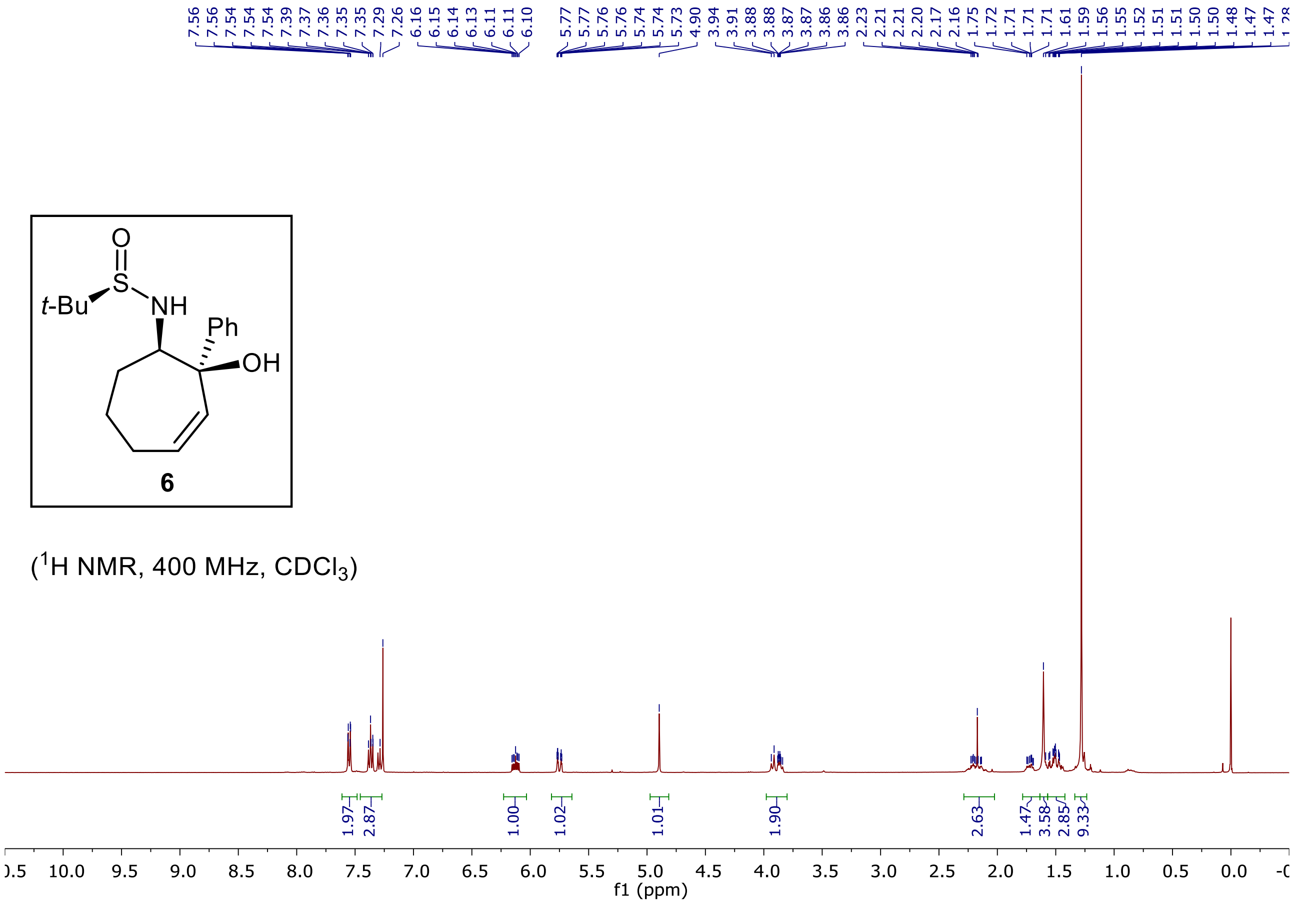
7.56 7.56 7.54 7.54 7.54 7.39 7.37 7.36 7.35 7.35 7.29 7.26 6.16 6.15 6.14 6.13 6.11 6.11 6.10 5.77 5.77 5.76 5.76 5.74 5.74 5.73 4.90 3.94 3.91 3.88 3.88 3.87 3.87 3.86 3.86 2.23 2.21 2.21 2.20 2.17 2.16 1.75 1.72 1.71 1.71 1.71 1.61 1.59 1.56 1.55 1.52 1.51 1.51 1.50 1.48 1.47 1.47
O
S
t-Bu
NH
Ph
OH
6
(¹H NMR, 400 MHz, CDCl₃)
1.97 2.87 1.00 1.02 1.01 1.90 2.63 1.47 3.58 2.85 9.33
10.0 9.5 9.0 8.5 8.0 7.5 7.0 6.5 6.0 5.5 5.0 4.5 4.0 3.5 3.0 2.5 2.0 1.5 1.0 0.5 0.0
f1 (ppm)

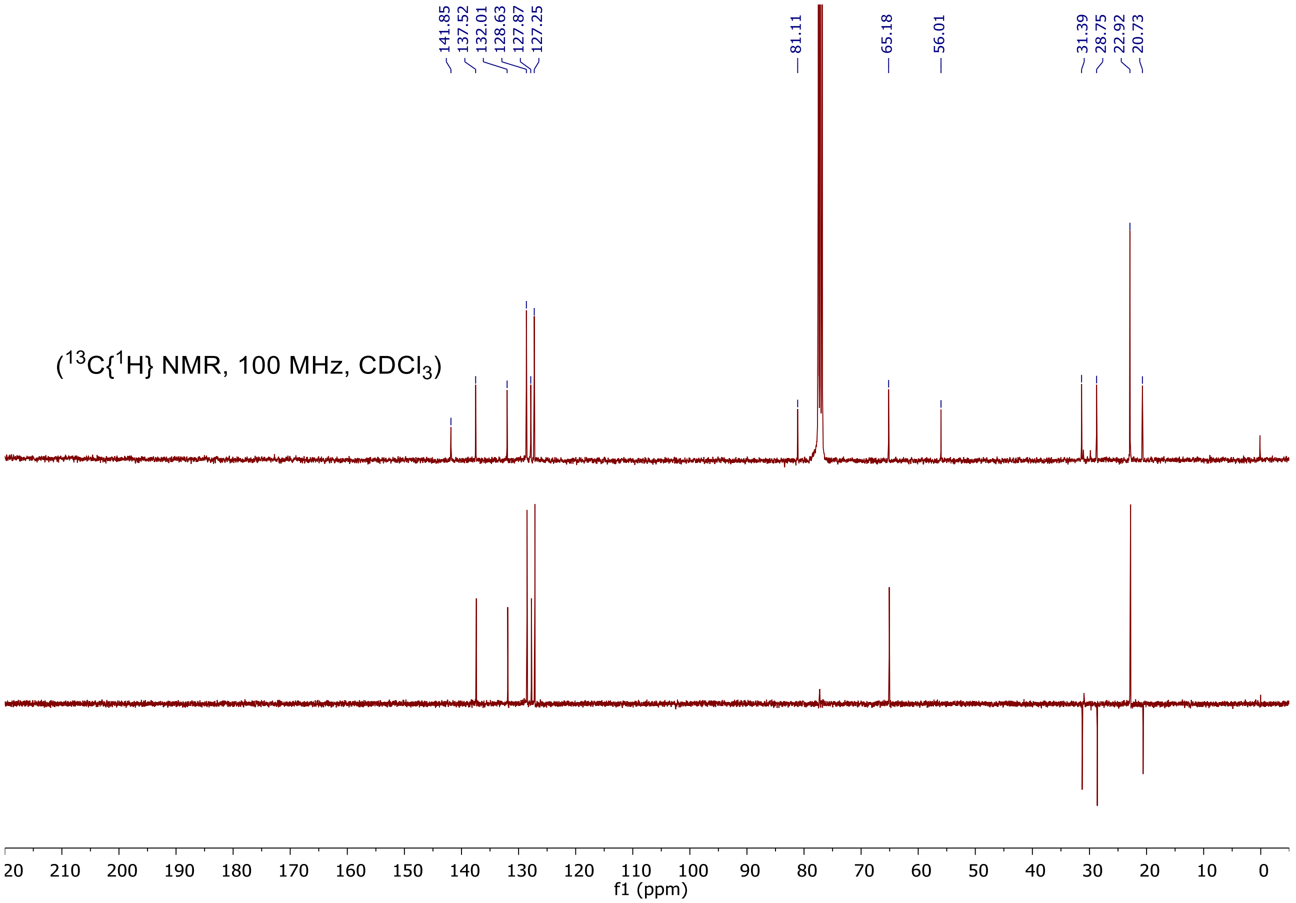
141.85 137.52 132.01 128.63 127.87 127.25
81.11
65.18
56.01
31.39 28.75 22.92 20.73
(¹³C{¹H} NMR, 100 MHz, CDCl₃)
210 200 190 180 170 160 150 140 130 120 110 100 90 80 70 60 50 40 30 20 10 0
f1 (ppm)

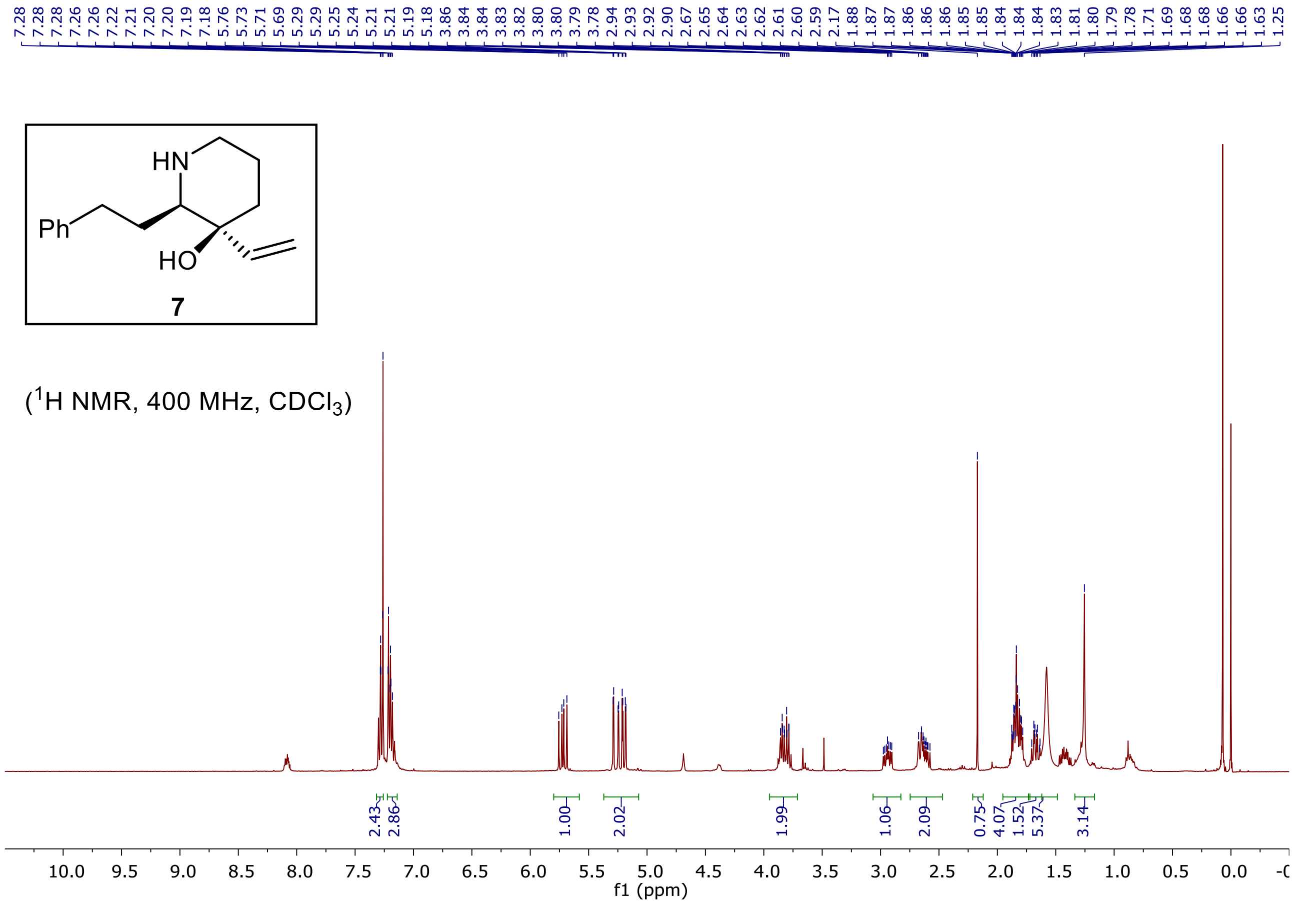
HN
Ph
HO
7
(1H NMR, 400 MHz, CDCl3)
f1 (ppm)

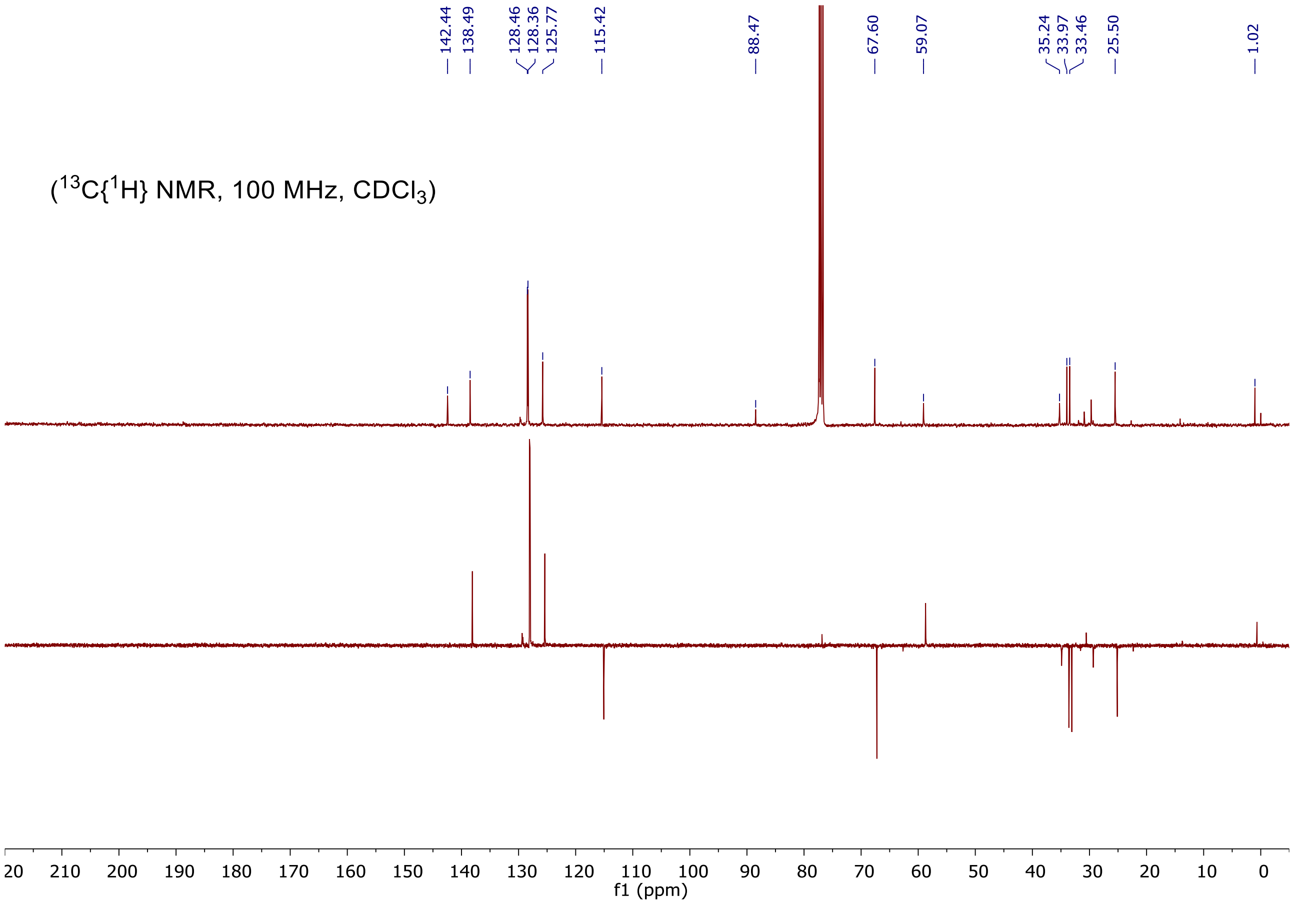
(13C{1H} NMR, 100 MHz, CDCl3)
f1 (ppm)

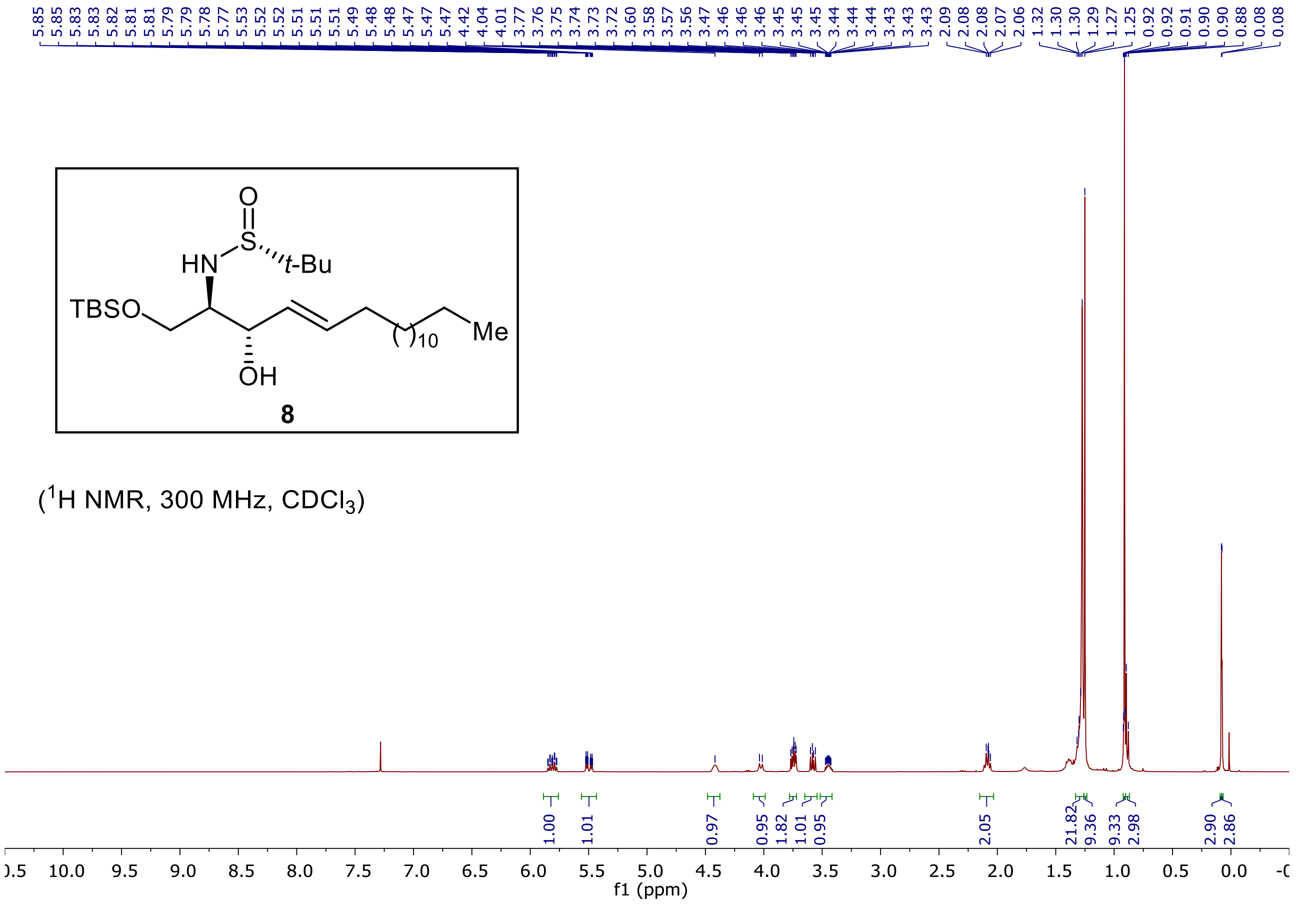

TBSO
HN
S
O
t-Bu
OH
( )10
Me
8
(¹H NMR, 300 MHz, CDCl3)
f1 (ppm)

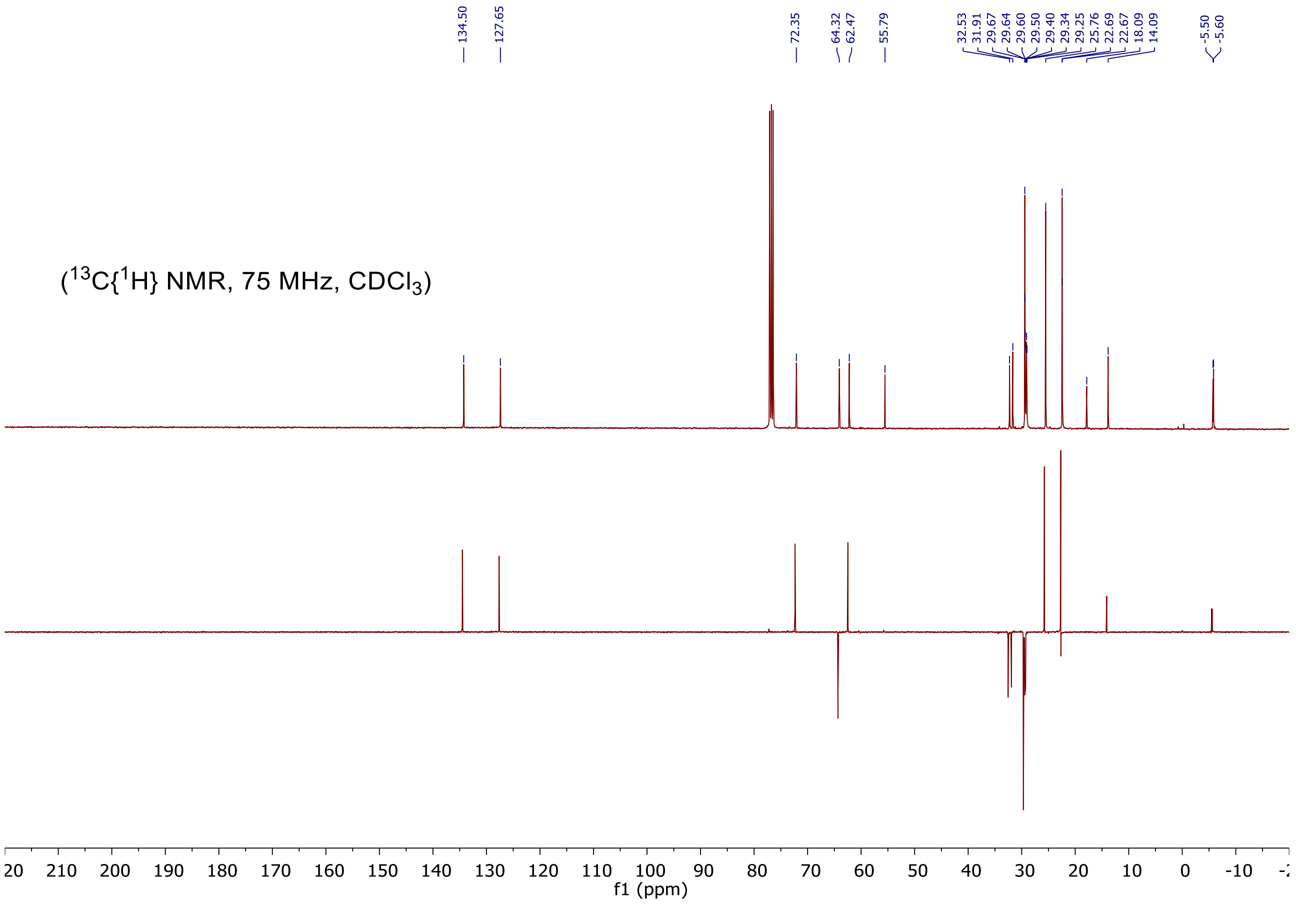

(¹³C{¹H} NMR, 75 MHz, CDCl3)
f1 (ppm)

X-ray structure of compound 3ab

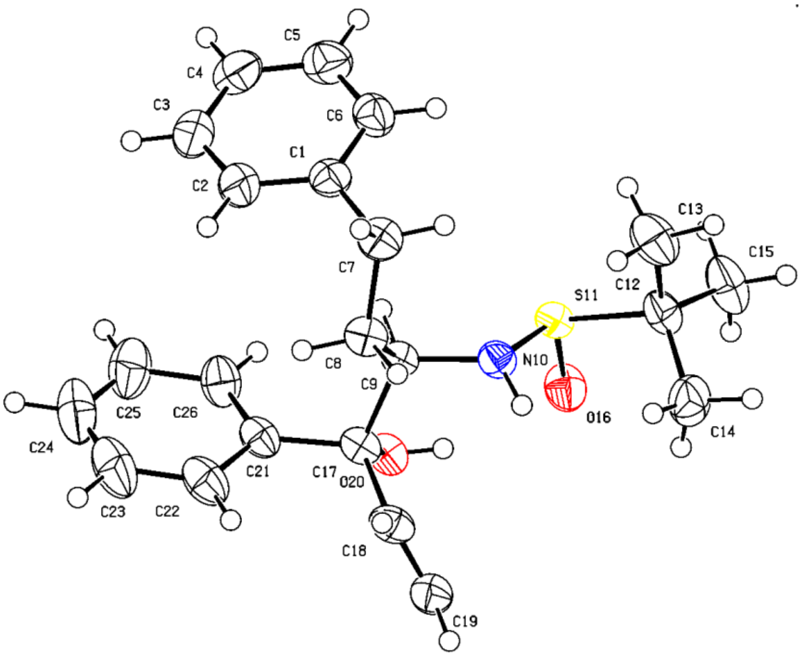

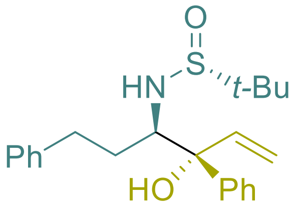

3ab

Figure S1. A displacement ellipsoid plot of **3ab** as determined by X-ray analysis. Non-H atoms are represented by 50% probability ellipsoids and H atoms are shown as small circles of arbitrary size.

Crystal data (excluding structure factors deposited at the Cambridge Crystallographic Data Centre as supplementary publication number CCDC 2309871): C22H29NO2S, M = 371.52; monoclinic, a = 5.76090(10) Å, b = 19.3873(3) Å, c = 9.4645(2) Å; V = 1041.87(3) Å^3; space group P2(1); Z = 4; D_c = 1.184 Mg m^{-3}; λ = 1.54184 Å; μ = 1.487 mm^{-1}; F(000) = 400; T = -73±2ºC. Data collection was performed on an Agilent SuperNova Atlas Dual Source. The diffraction frames were integrated using the program CrysAlisPro.[1] The structure was solved by direct methods[2] and refined to all 4014 unique F_o^2 by full matrix least squares.[2] An empirical absorption correction was performed using spherical harmonics.[3] Most of the hydrogen atoms were placed at idealised positions and all are refined as rigid atoms. Final $wR2$=0.0611 for all data and 243 parameters; R1=0.0219 for 3989 $F_o>4\sigma(F_o)$.

[1] CrysAlisPro 1.171.41.123a (Rigaku OD, 2022).

[2] SHELXL-2014/7 (Sheldrick, 2014).

[3] CrysAlisPro 1.171.41.123a (Rigaku OD, 2022).

X-ray structure of compound 3ai

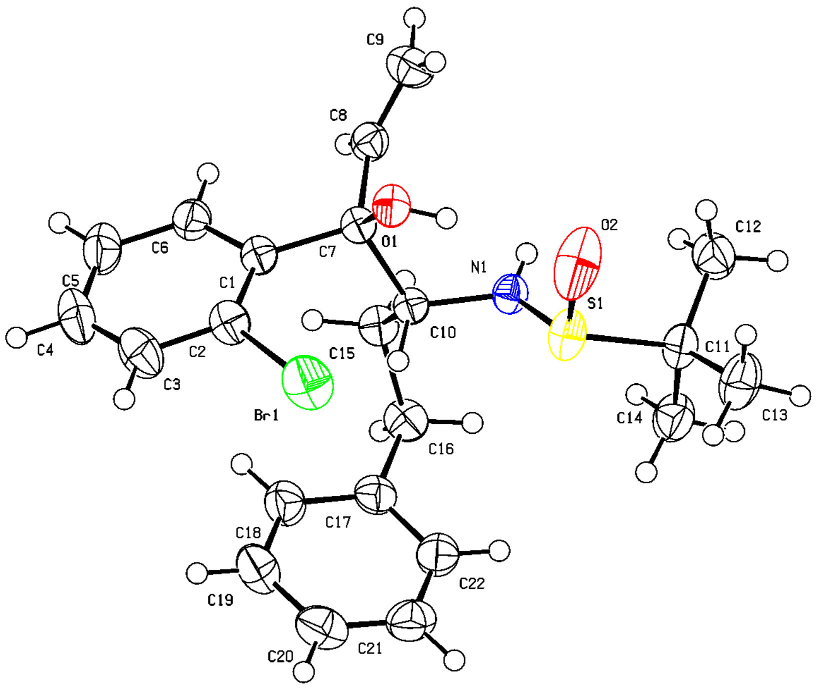

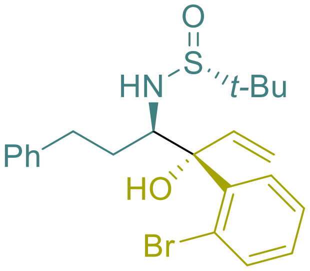

3ai

Figure S1. A displacement ellipsoid plot of **3ai** as determined by X-ray analysis. Non-H atoms are represented by 50% probability ellipsoids and H atoms are shown as small circles of arbitrary size.

Crystal data (excluding structure factors deposited at the Cambridge Crystallographic Data Centre as supplementary publication number CCDC 2309873): C22H28BrNO2S, M = 450.42; monoclinic, a = 10.3043(2) Å, b = 9.4193(2) Å, c = 11.16080(18) Å; V = 1069.18(4) Å^3; space group P2(1); Z = 4; D_c = 1.399 Mg m^{-3}; λ = 1.54184 Å; μ = 3.656 mm^{-1}; F(000) = 468; T = -30±2ºC. Data collection was performed on an Agilent SuperNova Atlas Dual Source. The diffraction frames were integrated using the program CrysAlisPro.[1] The structure was solved by direct methods[3] and refined to all 3967 unique F_o^2 by full matrix least squares.[2] An empirical absorption correction was performed using spherical harmonics.[3] Most of the hydrogen atoms were placed at idealised positions and all were refined as rigid atoms. Final $wR2$=0.0701for all data and 251 parameters; R1=0.0260 for 3967 $F_o>4\sigma(F_o)$.

[1] CrysAlisPro 1.171.41.123a (Rigaku OD, 2022).

[2] SHELXL-2014/7 (Sheldrick, 2014).

[3] CrysAlisPro 1.171.41.123a (Rigaku OD, 2022).
